# Supplementary material for: Gut Microbiome and Metabolome Alterations in Overweight or Obese Adult Population after Weight-Loss Bifidobacterium breve BBr60 Intervention: A Randomized Controlled Trial
Source: Int J Mol Sci. 2024 Oct 10;25(20):10871. doi: 10.3390/ijms252010871 (PMC11507383; doi:10.3390/ijms252010871)
Supplement: Supplementary file 1 [file ijms-25-10871-s001.zip › ijms-3223309-supplementary.pdf]

Table S1. Differential fecal metabolites before and after a 12-week BBr60 intervention on serum metabolism

**Name**	**MS2 score**	**mz**	**type**	**Formula**	**HMDB**	**CAS**	**KEGG ID**	**MEAN BBr60-before**	**MEAN BBr60-after**	**VIP**	**P-VALUE**	**FOLD CHANGE**
EDTA	3.99	293.098	POS	C10H16N2O8	HMDB0015109	62-33-9	C00284	0.1459	22.2848	1.7273	0.0002	152.7790
Asparagine	3.99	133.0607	POS	C4H8N2O3	HMDB0000168	70-47-3	C00152	0.0271	0.0472	1.2708	0.0002	1.7461
1,7-Dimethylxanthine	3.99	179.0575	NEG	C7H8N4O2	HMDB0001860	611-59-6	C13747	1.6303	1.0231	1.0869	0.0296	0.6276
Theobromine	3.98	179.0575	NEG	C7H8N4O2	HMDB0002825	83-67-0	C07480	1.6303	1.0231	1.0869	0.0296	0.6276
gamma-Glutamylglutamine	3.98	276.119	POS	C10H17N3O6	HMDB0011738	10148-81-9	C05283	0.0103	0.0411	1.6214	0.0000	3.9973
Citrulline	3.98	176.1029	POS	C6H13N3O3	HMDB0000904	372-75-8	C00327	0.0800	0.1292	1.0126	0.0063	1.6157
4-Acetamidobutyric acid	3.98	144.0667	NEG	C6H11NO3	HMDB0003681	3025-96-5	C02946	0.0556	0.0452	1.0599	0.0207	0.8142
Levulinic acid	3.98	115.0401	NEG	C5H8O3	HMDB0000720	123-76-2		0.5218	0.3510	1.6908	0.0000	0.6726
Serine	3.98	106.0498	POS	C3H7NO3	HMDB0000187	56-45-1	C00065	0.0172	0.0393	1.4500	0.0003	2.2894
Theophylline	3.97	179.0575	NEG	C7H8N4O2	HMDB0001889	58-55-9	C07130	1.6303	1.0231	1.0869	0.0296	0.6276
Glutamate	3.97	148.0605	POS	C5H9NO4	HMDB0000148	56-86-0	C00025	0.0616	0.1412	1.4087	0.0000	2.2914
Fumaric acid	3.97	115.0039	NEG	C4H4O4	HMDB0000134	110-17-8	C00122	0.0615	0.0396	1.3706	0.0002	0.6446
N-Acetylcarnosine	3.97	269.1244	POS	C11H16N4O4	HMDB0012881	56353-15-2		0.0074	0.0053	1.3823	0.0025	0.7146
Pantothenic acid	3.97	218.1035	NEG	C9H17NO5	HMDB0000210	79-83-4	C00864	0.2833	0.2181	1.0975	0.0023	0.7698
9-Oxo-10(E),12(E)-octadecadienoic acid	3.97	293.2121	NEG	C18H30O3	HMDB0004669	54232-58-5	C14766	0.3124	0.1732	1.1096	0.0025	0.5545
13(S)-HODE	3.97	295.228	NEG	C18H32O3	HMDB0004667	29623-28-7	C14762	0.8103	0.5687	1.2453	0.0010	0.7019
10,13-dimethyl-17-oxo-3-sulfooxy-1,2,3,4,7,8,9,11,12,14,15,16-dodecahydrocyclopenta[a]phenanthrene	3.97	367.1587	NEG	C19H28O5S	HMDB0001032	651-48-9	C04555	124.9684	92.4725	1.0703	0.0070	0.7400
gamma-Glutamyllysine	3.96	276.1555	POS	C11H21N3O5	HMDB0029154			0.0050	0.0075	1.3496	0.0004	1.5130
trans-Aconitic acid	3.96	173.0094	NEG	C6H6O6	HMDB0000958	4023-65-8	C02341	0.3090	1.2701	1.9518	0.0000	4.1105
cis-Aconitic acid	3.96	173.0094	NEG	C6H6O6	HMDB0000072	4023-65-8	C00417	0.3090	1.2701	1.9518	0.0000	4.1105
Taurine	3.96	124.0075	NEG	C2H7NO3S	HMDB0000251	107-35-7	C00245	17.8607	13.2360	1.2490	0.0120	0.7411
Betaine	3.96	118.086	POS	C5H11NO2	HMDB0000043	107-43-7	C00719	4.2156	5.0657	1.1453	0.0012	1.2016

3-Amino-4-methylpentanoic acid	3.96	130.0875	NEG	C6H13NO2	HMDB0245808	5699-54-7		9.3672	6.9147	1.2880	0.0012	0.7382
Isoleucine	3.96	130.0875	NEG	C6H13NO2	HMDB0000172	73-32-5	C00407	9.3672	6.9147	1.2880	0.0012	0.7382
Inosine	3.96	269.0881	POS	C10H12N4O5	HMDB0000195	58-63-9	C00294	0.0353	0.0193	1.2586	0.0080	0.5463
1-Methylinosine	3.96	283.1035	POS	C11H14N4O5	HMDB0002721	2140-73-0		0.0254	0.0294	1.0441	0.0025	1.1580
Taurochenodeoxycholic acid	3.96	498.29	NEG	C26H45NO6S	HMDB0000951	516-35-8	C05465	0.9548	0.5889	1.0037	0.0243	0.6168
10-Undecenoic acid	3.96	183.1392	NEG	C11H20O2	HMDB0033724	1333-28-4	C13910	0.7678	0.4904	1.1294	0.0003	0.6387
3-Hydroxylauric acid	3.96	197.1549	NEG	C12H24O3	HMDB0000387	45162-48-9		1.2942	0.8744	1.2303	0.0003	0.6757
Denticetic-acid	3.95	197.1549	NEG	C12H22O2	HMDB0000529	2430-94-6		1.2942	0.8744	1.2303	0.0003	0.6757
Homoserine	3.95	118.0511	NEG	C4H9NO3	HMDB0000719	672-15-1	C00263	0.6086	0.8146	1.3136	0.0319	1.3384
Ergothioneine	3.95	230.0956	POS	C9H15N3O2S	HMDB0003045	497-30-3	C05570	0.0076	0.0147	1.1391	0.0023	1.9385
Uric acid	3.95	167.0213	NEG	C5H4N4O3	HMDB0000289	69-93-2	C00366	0.5376	0.9378	1.1786	0.0004	1.7444
3-Hydroxybutyrylcarnitine (Car(4:0-O))	3.95	248.1491	POS	C11H21NO5	HMDB0013127	1469900-92-2		0.0410	0.0228	1.3803	0.0096	0.5562
5-Methyl DL-glutamate	3.95	162.0761	POS	C6H11NO4	HMDB0061715	14487-45-7		0.0362	0.0243	1.5163	0.0000	0.6707
Tryptophan	3.95	205.0969	POS	C11H12N2O2	HMDB0030396	73-22-3	C00078	0.8632	1.0785	1.0407	0.0027	1.2493
Pseudouridine	3.95	243.0625	NEG	C9H12N2O6	HMDB0000767	1445-07-4	C02067	1.1204	0.9240	1.2271	0.0048	0.8247
N,N-Diethyl-2-aminoethanol	3.95	100.112	POS	C6H15NO	HMDB0033971	100-37-8		0.0113	0.0486	1.6022	0.0000	4.3214
2'-O-methylcytidine	3.95	258.1083	POS	C10H15N3O5	HMDB0242132	2140-72-9		0.0052	0.0060	1.3661	0.0004	1.1381
Nordeoxycholic acid	3.95	377.2704	NEG	C23H38O4	HMDB0304947	53608-86-9		0.0858	0.0590	1.4689	0.0001	0.6876
Creatinine	3.95	112.0519	NEG	C4H7N3O	HMDB0000562	60-27-5	C00791	0.2551	0.2046	1.3780	0.0026	0.8018
5,6-Dihydrouridine	3.95	247.0923	POS	C9H14N2O6	HMDB0000497	5627-05-4		0.0061	0.0074	1.1537	0.0030	1.2068
3-Hydroxydecanoic acid	3.95	187.1341	NEG	C10H20O3	HMDB0002203	19526-23-9		0.7726	0.5047	1.0849	0.0088	0.6532
Valproic acid	3.95	143.1079	NEG	C8H16O2	HMDB0001877	99-66-1	C07185	0.5646	0.4778	1.0015	0.0162	0.8463
3-Methyl-2-oxovaleric acid	3.95	129.0558	NEG	C6H10O3	HMDB0000491	1460-34-0	C03465	135.4522	104.8729	1.3475	0.0009	0.7742
2-Ketocaproic acid	3.95	129.0558	NEG	C6H10O3	HMDB0001864	2492-75-3	C00902	135.4522	104.8729	1.3475	0.0009	0.7742
4-Methylquinolin-2-ol	3.95	158.0613	NEG	C10H9NO		607-66-9		2.0079	1.1625	1.5989	0.0007	0.5790
3'-O-methylcytidine	3.94	258.1083	POS	C10H15N3O5	HMDB0304396	20594-00-7		0.0052	0.0060	1.3661	0.0004	1.1381

Caprylic acid	3.94	143.1079	NEG	C8H16O2	HMDB0000482	124-07-2	C06423	0.5646	0.4778	1.0015	0.0162	0.8463
Ketoleucine	3.94	129.0558	NEG	C6H10O3	HMDB0000695	816-66-0	C00233	135.4522	104.8729	1.3475	0.0009	0.7742
beta-Alanine	3.94	88.0405	NEG	C3H7NO2	HMDB0000056	107-95-9	C00099	2.9443	2.3777	1.2763	0.0006	0.8076
Valine	3.94	116.0718	NEG	C5H11NO2	HMDB0000883	72-18-4	C00183	6.5520	5.1346	1.3824	0.0022	0.7837
Imidazol-1-yl-acetic acid	3.94	127.0502	POS	C5H6N2O2	HMDB0029736	22884-10-2		0.1044	0.4868	1.3705	0.0001	4.6651
N-Acetyl-beta-alanine	3.94	130.0511	NEG	C5H9NO3	HMDB0061880	3025-95-4	C01073	0.1374	0.0690	1.1619	0.0004	0.5024
Pyruvate	3.94	87.0088	NEG	C3H4O3	HMDB0000243	127-17-3	C00022	136.6179	99.5917	1.2218	0.0001	0.7290
Lysine	3.93	147.1128	POS	C6H14N2O2	HMDB0000182	56-87-1	C00047	0.6402	0.8554	1.5248	0.0004	1.3361
3-methylcytidine	3.93	258.1086	POS	C10H15N3O5	HMDB0000982	2140-61-6		0.0037	0.0045	1.0190	0.0176	1.2219
4-Hydroxyphenylacetic acid	3.93	151.0402	NEG	C8H8O3	HMDB0000020	156-38-7	C00642	0.0456	0.0324	1.0519	0.0207	0.7100
Leu-Leu	3.93	243.1714	NEG	C12H24N2O3	HMDB0028933	3303-31-9	C11332	0.0898	0.0497	1.5008	0.0009	0.5541
Ile-Leu	3.93	243.1714	NEG	C12H24N2O3	HMDB0028911	26462-22-6		0.0898	0.0497	1.5008	0.0009	0.5541
2-Methylbutyrylglycine	3.93	158.0824	NEG	C7H13NO3	HMDB0000339	52320-67-9		0.1059	0.0649	1.4933	0.0034	0.6130
2-Hydroxyoctanoic acid	3.93	159.1028	NEG	C8H16O3	HMDB0000711	617-73-2		0.8397	0.5650	1.1681	0.0019	0.6729
Citraconic acid	3.93	129.0196	NEG	C5H6O4	HMDB0000634	498-23-7	C02226	0.5250	0.3037	1.3855	0.0001	0.5785
Acetoin	3.93	87.0453	NEG	C4H8O2	HMDB0003243	513-86-0	C00466	0.2368	0.1847	1.3055	0.0021	0.7802
Embelin	3.93	293.176	NEG	C17H26O4	HMDB0251767	550-24-3	C10342	3.0092	2.4341	1.0208	0.0045	0.8089
Pelargonic acid	3.93	157.1236	NEG	C9H18O2	HMDB0000847	112-05-0	C01601	3.1850	2.4304	1.3917	0.0004	0.7631
Isoquinoline	3.93	130.065	POS	C9H7N	HMDB0034244	119-65-3	C06323	0.0253	0.0147	1.6346	0.0005	0.5802
alpha-Linolenic acid	3.93	277.2177	NEG	C18H30O2	HMDB0001388	463-40-1	C06427	13.7038	9.9151	1.1062	0.0004	0.7235
PC(16:0/18:1(9Z))	3.93	742.5737	POS	C42H82NO8P	HMDB0007972		C13875	0.2324	0.2927	1.0119	0.0088	1.2592
Terephthalic-Acid	3.92	165.0196	NEG	C8H6O4	HMDB0002428	100-21-0	C06337	0.1668	0.1252	1.2914	0.0096	0.7506
Azelaic acid	3.92	187.0979	NEG	C9H16O4	HMDB0000784	123-99-9	C08261	0.0856	0.0554	1.6354	0.0007	0.6471
LPC(17:0/0:0)	3.92	510.3555	POS	C25H52NO7P	HMDB0012108	50930-23-9		0.4062	0.6044	1.7623	0.0000	1.4878
3-(3-Hydroxyphenyl)propanoic acid	3.92	165.0559	NEG	C9H10O3	HMDB0000375	621-54-5	C11457	0.1607	0.1215	1.0292	0.0296	0.7565
trans-2-Octenoic acid	3.92	141.0923	NEG	C8H14O2	HMDB0001568	1871-67-6		0.5244	0.3713	1.3082	0.0019	0.7081

alpha-Ketoisovaleric acid	3.92	115.0402	NEG	C5H8O3	HMDB0000019	759-05-7	C00141	42.4085	32.3576	1.3527	0.0012	0.7630
5,6-Dihydroxyindole	3.92	148.0406	NEG	C8H7NO2	HMDB0004058	3131-52-0	C05578	0.0998	0.0642	1.3638	0.0004	0.6438
Capric acid	3.92	171.1392	NEG	C10H20O2	HMDB0000511	334-48-5	C01571	1.1614	0.8561	1.2560	0.0005	0.7372
Undecanoic acid	3.92	185.1549	NEG	C11H22O2	HMDB0000947	112-37-8	C17715	0.3067	0.2019	1.4993	0.0000	0.6584
Taurohyodeoxycholic acid	3.91	498.29	NEG	C26H45NO6S	HMDB0247202	386523		0.9548	0.5889	1.0037	0.0243	0.6168
gamma-Linolenic acid	3.91	277.2177	NEG	C18H30O2	HMDB0003073	506-26-3	C06426	13.7038	9.9151	1.1062	0.0004	0.7235
Phosphorylcholine	3.91	184.0733	POS	C5H15NO4P	HMDB0001565	3616-04-4	C00588	0.0231	0.0394	1.0650	0.0018	1.7078
O-Acetylserine	3.91	146.046	NEG	C5H9NO4	HMDB0003011	5147-00-2	C00979	0.7925	0.6283	1.4320	0.0199	0.7928
N-Acetyltryptophan	3.91	245.0932	NEG	C13H14N2O3	HMDB0013713	1218-34-4	C03137	0.0592	0.0373	1.5387	0.0008	0.6294
N2,N2-Dimethylguanosine	3.91	312.1302	POS	C12H17N5O5	HMDB0004824	2140-67-2		0.0370	0.0436	1.2414	0.0009	1.1781
Leu-Ile	3.9	243.1714	NEG	C12H24N2O3	HMDB0028932	36077-41-5		0.0898	0.0497	1.5008	0.0009	0.5541
Citric acid	3.9	191.0201	NEG	C6H8O7	HMDB0000094	77-92-9	C00158	0.1067	1.2718	1.2267	0.0263	11.9227
Malonic acid	3.9	103.0039	NEG	C3H4O4	HMDB0000691	141-82-2	C00383	0.3063	0.2356	1.0794	0.0234	0.7691
N-Methyl-L-asparagine	3.9	127.0515	NEG	C5H10N2O3		7175-34-0		0.0422	0.7850	1.8533	0.0001	18.5964
LPC(16:0)	3.9	496.3392	POS	C24H50NO7P	HMDB0010382	17364-16-8		33.4970	50.4719	2.2038	0.0000	1.5068
Benzoic acid	3.9	121.0296	NEG	C7H6O2	HMDB0001870	65-85-0	C00180	1.4314	1.0492	1.6095	0.0000	0.7330
Tauroursodeoxycholic acid	3.89	498.29	NEG	C26H45NO6S	HMDB0000874	14605-22-2	C16868	0.9548	0.5889	1.0037	0.0243	0.6168
MeAIB	3.89	118.0861	POS	C5H11NO2	HMDB0002141	2566-34-9		3.5884	3.9415	1.1111	0.0169	1.0984
Glycolate	3.89	75.0089	NEG	C2H4O3	HMDB0000115	79-14-1	C00160	0.4281	0.3502	1.2746	0.0010	0.8179
Allantoin	3.89	157.0369	NEG	C4H6N4O3	HMDB0000462	97-59-6	C01551	0.4235	0.2284	1.9105	0.0000	0.5393
N-Lactoylphenylalanine	3.89	236.0931	NEG	C12H15NO4	HMDB0062175	183241-73-8		0.0765	0.0426	1.3783	0.0029	0.5578
4-Ethoxybenzoic acid	3.89	165.0558	NEG	C9H10O3		619-86-3		1.9589	1.2518	1.6838	0.0000	0.6390
3-Hydroxypyruvic acid	3.88	103.0039	NEG	C3H4O4	HMDB0001352	1113-60-6	C00168	0.3063	0.2356	1.0794	0.0234	0.7691
N,N-Dimethylarginine (ADMA)	3.88	203.1502	POS	C8H18N4O2	HMDB0001539	30315-93-6	C03626	0.5911	0.7649	1.2938	0.0004	1.2939
1-O-Hexadecyl-sn-glycero-3-phosphocholine (LPC(O-16:0/0:0))	3.88	482.3602	POS	C24H52NO6P	HMDB0243890	52691-62-0	C13903	0.5968	0.9721	2.0769	0.0000	1.6288
Isobutyrylglycine	3.88	144.0667	NEG	C6H11NO3	HMDB0000730	15926-18-8		0.0592	0.0369	1.5254	0.0000	0.6229

PC(P-16:0/0:0)	3.88	480.3449	POS	C24H50NO6P	HMDB0010407	97802-53-4		0.5429	0.8781	2.0163	0.0000	1.6175
Kynurenic acid	3.88	188.0355	NEG	C10H7NO3	HMDB0000715	492-27-3	C01717	0.4068	0.2684	1.5652	0.0000	0.6598
N-Acetylalanine	3.88	130.0512	NEG	C5H9NO3	HMDB0255053	139146-66-0		0.2640	0.2162	1.2892	0.0155	0.8190
p-Toluquinone	3.87	121.0296	NEG	C7H6O2		553-97-9		1.4314	1.0492	1.6095	0.0000	0.7330
Pipecolamide	3.87	129.1022	POS	C6H12N2O		19889-77-1	C19809	0.0148	0.0217	1.0043	0.0120	1.4603
Ribothymidine	3.87	257.078	NEG	C10H14N2O6	HMDB0000884	1463-10-1		0.3518	0.2887	1.5284	0.0005	0.8206
Arabinono-1,4-lactone	3.87	147.03	NEG	C5H8O5	HMDB0001900	51532-86-6	C01114	0.4794	0.2138	1.7316	0.0000	0.4459
Paracetamol (Drug)	3.87	152.0705	POS	C8H9NO2	HMDB0001859	103-90-2	C06804	0.0070	0.0063	1.1926	0.0001	0.8987
N-Lactoyl-Phenylalanine	3.86	236.0931	NEG	C12H15NO4	HMDB0062175	183241-73-8		0.0765	0.0426	1.3783	0.0029	0.5578
Oxalic acid	3.86	88.9882	NEG	C2H2O4	HMDB0002329	144-62-7	C00209	0.1071	0.0881	1.0542	0.0076	0.8226
Formylmethionine	3.86	176.0388	NEG	C6H11NO3S	HMDB0001015	4289-98-9	C03145	0.2790	0.2341	1.1371	0.0131	0.8392
Car(18:1)	3.86	426.358	POS	C25H47NO4	HMDB0013337	13962-05-5		0.4375	0.5229	1.2723	0.0125	1.1952
Ganoderic acid DM	3.86	467.3158	NEG	C30H44O4		173075-45-1		0.1352	0.1069	1.0483	0.0050	0.7907
Valeric acid	3.86	101.0609	NEG	C5H10O2	HMDB0000892	109-52-4	C00803	0.5934	0.4726	1.2941	0.0043	0.7964
Isovaleric acid	3.85	101.0609	NEG	C5H10O2	HMDB0000718	503-74-2	C08262	0.5934	0.4726	1.2941	0.0043	0.7964
Aspartate	3.85	132.0305	NEG	C4H7NO4	HMDB0000191	56-84-8	C00049	0.0477	0.0958	1.1056	0.0013	2.0092
Phenylacetic acid	3.85	135.0453	NEG	C8H8O2	HMDB0000209	103-82-2	C07086	0.0817	0.0570	1.6319	0.0010	0.6982
12-Hydroxydodecanoic acid	3.85	215.1654	NEG	C12H24O3	HMDB0002059	505-95-3	C08317	0.3578	0.2569	1.2195	0.0014	0.7180
Pyridoxine	3.84	152.0705	POS	C8H11NO3	HMDB0000239	65-23-6	C00314	0.0070	0.0063	1.1926	0.0001	0.8987
Malic acid	3.84	133.0145	NEG	C4H6O5	HMDB0000744	6915-15-7	C00149	0.2590	0.3855	1.1390	0.0011	1.4886
Arachidonoylcarnitine (Car(20:4))	3.84	448.3421	POS	C27H45NO4	HMDB0006455	36816-11-2		0.0220	0.0291	1.3450	0.0073	1.3249
5-Methylcytidine	3.83	258.1086	POS	C10H15N3O5	HMDB0000982	2140-61-6		0.0037	0.0045	1.0190	0.0176	1.2219
Isocitric acid	3.83	191.0201	NEG	C6H8O7	HMDB0000193	320-77-4	C00311	0.1067	1.2718	1.2267	0.0263	11.9227
Lauramine oxide	3.83	230.2477	POS	C14H31NO		1643-20-5		0.2168	0.3495	2.2222	0.0000	1.6118
2,2-Dimethylsuccinic acid	3.83	145.0507	NEG	C6H10O4	HMDB0002074	597-43-3		0.3861	0.3049	1.3859	0.0002	0.7898
Lumichrome	3.83	241.0722	NEG	C12H10N4O2	HMDB0254199	1086-80-2	C01727	0.2955	0.1928	1.2553	0.0073	0.6526

Phthalide	3.83	135.044	POS	C8H6O2	HMDB0032469	87-41-2	C18611	0.0124	0.0067	1.8407	0.0001	0.5356
3-Hydroxyphenylacetic acid	3.82	151.0402	NEG	C8H8O3	HMDB0000440	621-37-4	C05593	0.0456	0.0324	1.0519	0.0207	0.7100
3-Methyluridine	3.82	257.078	NEG	C10H14N2O6	HMDB0004813	2140-69-4		0.3518	0.2887	1.5284	0.0005	0.8206
Methylmalonic acid	3.82	117.0195	NEG	C4H6O4	HMDB0000202	516-05-2	C02170	0.1344	0.0998	1.3284	0.0169	0.7425
Monomethyl phthalate	3.82	179.0348	NEG	C9H8O4	HMDB0002130	4376-18-5		0.2337	0.1730	1.2402	0.0092	0.7405
Taurodeoxycholic acid	3.81	498.29	NEG	C26H45NO6S	HMDB0000896	516-50-7	C05463	0.9548	0.5889	1.0037	0.0243	0.6168
6-Hydroxynorleucine	3.81	146.0824	NEG	C6H13NO3		6033-32-5		0.1741	0.1265	1.5512	0.0000	0.7269
3-Methyl-1H-pyrazole-4-carbaldehyde	3.81	111.0551	POS	C5H6N2O		112758-40-4		0.5123	0.2676	1.6109	0.0005	0.5223
Bisindolylmaleimide I	3.81	411.1852	NEG	C25H24N4O2	HMDB0249269	133052-90-1	C11238	1.3747	1.0283	1.0701	0.0110	0.7480
3-Methoxyphenylacetic acid	3.8	165.0559	NEG	C9H10O3	HMDB0059969	1798-09-0		0.1607	0.1215	1.0292	0.0296	0.7565
10-Hydroxydecanoic acid	3.79	187.1341	NEG	C10H20O3	HMDB0244272	1679-53-4	C02774	0.7726	0.5047	1.0849	0.0088	0.6532
2-Phenyllactic acid	3.79	165.0558	NEG	C9H10O3	HMDB0142137	515-30-0		1.9589	1.2518	1.6838	0.0000	0.6390
Butanoic acid	3.78	87.0453	NEG	C4H8O2	HMDB0000039	107-92-6	C00246	0.2368	0.1847	1.3055	0.0021	0.7802
Ethyl 3-hydroxybenzoate	3.78	165.0558	NEG	C9H10O3		7781-98-8		1.9589	1.2518	1.6838	0.0000	0.6390
Glu-Arg	3.78	304.1616	POS	C11H21N5O5	HMDB0028813	7219-59-2		0.0068	0.0137	1.3855	0.0008	2.0116
Gluconic acid	3.78	195.0512	NEG	C6H12O7	HMDB0000625	526-95-4	C00257	0.0769	0.1675	1.2972	0.0043	2.1795
4-Isopropylbenzoic acid	3.78	163.0766	NEG	C10H12O2	HMDB0035268	536-66-3	C06578	0.1002	0.0546	1.3373	0.0008	0.5454
Ethyl 4-hydroxybenzoate	3.77	165.0558	NEG	C9H10O3	HMDB0032573	120-47-8	D01647	1.9589	1.2518	1.6838	0.0000	0.6390
Xanthoxylin	3.77	195.0663	NEG	C10H12O4	HMDB0029645	90-24-4	C10726	0.2379	0.0713	1.4521	0.0001	0.2998
Diacetyl	3.77	85.0296	NEG	C4H6O2	HMDB0003407	431-03-8	C00741	1.0297	0.5984	1.5425	0.0000	0.5811
Diglycine	3.76	131.0465	NEG	C4H8N2O3	HMDB0011733	556-50-3	C02037	0.1770	0.2570	1.0892	0.0058	1.4522
Isoxanthopterin	3.76	180.0492	POS	C6H5N5O2	HMDB0000704	529-69-1	C03975	0.3006	0.3378	1.0907	0.0070	1.1236
5-Oxooctanoic acid	3.76	157.0871	NEG	C8H14O3		3637-14-7		0.5312	0.3248	1.7555	0.0000	0.6114
Docebenone	3.75	325.1846	NEG	C21H26O3	HMDB0247732	80809-81-0	C01349	18.8127	14.2110	1.1772	0.0100	0.7554
Pi-Methylimidazoleacetic acid (hydrochloride)	3.74	141.0659	POS	C6H8N2O2	HMDB0004988	4200-48-0		0.0055	0.0122	1.8622	0.0001	2.2029
1,3,7-Trimethyluric acid	3.74	211.0826	POS	C8H10N4O3	HMDB0002123	5415-44-1	C16361	0.0018	0.0039	1.2528	0.0000	2.1960

Isatin	3.74	146.025	NEG	C8H5NO2	HMDB0061933	1186480-61-4	C11129	0.1756	0.0846	1.3696	0.0045	0.4818
Caplamin	3.73	130.0875	NEG	C6H13NO2	HMDB0001901	60-32-2	C02378	9.3672	6.9147	1.2880	0.0012	0.7382
4-(dimethylamino)butanoate	3.73	130.0875	NEG	C6H13NO2		693-11-8		9.3672	6.9147	1.2880	0.0012	0.7382
Desaminotyrosine	3.73	165.0558	NEG	C9H10O3	HMDB0002199	501-97-3	C01744	1.9589	1.2518	1.6838	0.0000	0.6390
3-Hydroxyacetophenone	3.73	135.0453	NEG	C8H8O2		121-71-1		0.0817	0.0570	1.6319	0.0010	0.6982
4-Hydroxyphenylacetaldehyde	3.73	135.0453	NEG	C8H8O2	HMDB0003767	7339-87-9	C03765	0.0817	0.0570	1.6319	0.0010	0.6982
Phosphate	3.73	96.9698	NEG	H3O4P	HMDB0001429	7664-38-2	C00009	0.8104	1.1962	1.6067	0.0012	1.4760
7-Ketodeoxycholic acid	3.73	405.2651	NEG	C24H38O5	HMDB0000391	911-40-0	C04643	0.1550	0.1136	1.3231	0.0022	0.7327
3-Oxocholic acid	3.73	405.2651	NEG	C24H38O5	HMDB0000502	2304-89-4		0.1550	0.1136	1.3231	0.0022	0.7327
Quillaic acid	3.73	485.3263	NEG	C30H46O5		631-01-6	C08972	0.0711	0.0505	1.4811	0.0008	0.7094
PC(17:0/17:0)	3.73	744.5894	POS	C42H84NO8P		70897-27-7		0.6935	0.8309	1.0155	0.0496	1.1982
PC(16:0/18:0)	3.73	744.5894	POS	C42H84NO8P	HMDB0007970	59403-51-9		0.6935	0.8309	1.0155	0.0496	1.1982
PC(18:0/16:0)	3.73	744.5894	POS	C42H84NO8P	HMDB0008034	59403-53-1		0.6935	0.8309	1.0155	0.0496	1.1982
Bilirubin	3.73	583.2568	NEG	C33H36N4O6	HMDB0000054	635-65-4	C00486	0.7109	1.6438	1.1947	0.0003	2.3124
3-(2-Hydroxyphenyl)propanoic acid	3.72	165.0559	NEG	C9H10O3	HMDB0033752	495-78-3	C01198	0.1607	0.1215	1.0292	0.0296	0.7565
3-Hydroxyoctanoic acid	3.72	141.0923	NEG	C8H16O3	HMDB0001954	14292-27-4	C20793	0.5244	0.3713	1.3082	0.0019	0.7081
4'-Hydroxyacetophenone	3.72	135.0453	NEG	C8H8O2		99-93-4	C10700	0.0817	0.0570	1.6319	0.0010	0.6982
(1-Hydroxycyclohexyl)acetic acid	3.72	157.0871	NEG	C8H14O3		14399-63-4		0.5312	0.3248	1.7555	0.0000	0.6114
Pyruvaldehyde	3.72	71.014	NEG	C3H4O2	HMDB0001167	78-98-8	C00546	3.0346	2.1329	1.8162	0.0000	0.7029
Palmitoylcarnitine (Car(16:0))	3.72	400.3421	POS	C23H45NO4	HMDB0000222	2364-67-2	C02990	0.2844	0.3900	1.5907	0.0000	1.3713
Dihydrouracil	3.72	95.0252	NEG	C4H6N2O2	HMDB0000076	504-07-4	C00429	0.1872	0.1322	1.2879	0.0022	0.7061
Royal jelly acid	3.72	185.1184	NEG	C10H18O3	HMDB0244269	14113-05-4		1.5121	0.8376	1.2775	0.0006	0.5539
Phthalic acid	3.71	165.0196	NEG	C8H6O4	HMDB0002107	88-99-3	C01606	0.1668	0.1252	1.2914	0.0096	0.7506
Tetrahydrocorticosterone	3.71	349.2386	NEG	C21H34O4	HMDB0000268	68-42-8	C05476	0.1150	0.0882	1.2387	0.0004	0.7667
AKBA	3.71	511.3417	NEG	C32H48O5		67416-61-9		0.0401	0.0328	1.1056	0.0120	0.8175
5-Hydroxyvalproic acid	3.69	159.1028	NEG	C8H16O3	HMDB0013898	53660-23-4	C16650	0.8397	0.5650	1.1681	0.0019	0.6729

Bisindolylmaleimide VIII (acetate)	3.65	397.1693	NEG	C24H22N4O2	HMDB0257261	125313-65-7		0.4154	0.3192	1.1119	0.0115	0.7685
PC(10:0/10:0)	3.54	548.3641	POS	C28H56NO8P	HMDB0244070	3436-44-0		0.0038	0.0152	1.6539	0.0007	4.0202
7,3',4',5'-Tetrahydroxyflavone	2.75	287.055	POS	C15H10O6				0.0007	0.0346	1.7951	0.0001	48.8561
3-(3-Hydroxypropyl)-3,5-dihydro-4H-pyrimido[5,4-b]indol-4-one	2.75	244.1081	POS	C13H13N3O2				0.0089	0.0061	1.4733	0.0018	0.6914
1-Octadecyl-2-acetyl-sn-glycero-3-phosphocholine	2.75	552.4025	POS	C28H58NO7P				0.0976	0.1233	1.3870	0.0007	1.2630
[(Aminocarbonyl)amino](phenyl)acetic acid	2.75	193.0619	NEG	C9H10N2O3				0.0465	0.0340	1.3998	0.0001	0.7308
cis-7-Hexadecenoic acid	2.75	253.2173	NEG	C16H30O2	HMDB0002186	2416-19-5		0.1926	0.1400	1.0253	0.0070	0.7271
Dihexadecyldimethylammonium cation	2.75	494.5659	POS	C34H72N				0.0068	0.0104	1.6269	0.0000	1.5174
HYDROCOTARNINE	2.75	222.1125	POS	C12H15NO3	HMDB0033701	550-10-7	C13534	0.4738	0.5463	1.1784	0.0026	1.1531
N-Methylvaline	2.75	132.1019	POS	C6H13NO2	HMDB0061716			0.0209	0.0425	1.5805	0.0052	2.0345
Ethyldodecanoate	2.75	227.2016	NEG	C14H28O2	HMDB0033788	106-33-2		0.1590	0.1333	1.0354	0.0162	0.8384
PC(38:6)	2.74	806.5692	POS	C46H80NO8P				0.0439	0.0564	1.3346	0.0050	1.2833
PC(42:4)	2.74	848.6523	POS	C50H92NO8P				0.1488	0.1813	1.3836	0.0014	1.2184
N-Methyl-L-prolinamide	2.74	129.1023	POS	C6H12N2O				0.0097	0.0684	1.2781	0.0067	7.0558
(3-Ethyl-2-imino-2,3-dihydro-1H-benzimidazol-1-yl)acetic acid	2.74	220.1079	POS	C11H13N3O2				0.0562	0.0454	1.3465	0.0007	0.8074
His-Trp	2.74	342.1559	POS	C17H19N5O3	HMDB0028896	23403-90-9		0.0604	0.0345	1.6475	0.0017	0.5710
2-Methylbenzamide oxime	2.74	151.0866	POS	C8H10N2O	HMDB0341238			0.0143	0.0083	1.9256	0.0000	0.5767
N-Methylpropionamide	2.74	88.0757	POS	C4H9NO				0.0825	0.4499	1.5945	0.0000	5.4568
Glycerophospho-N-palmitoylethanolamine	2.74	454.2931	POS	C21H44NO7P				0.1678	0.2262	1.6325	0.0000	1.3476
1-O-Hexadecyl-2-O-(2E-butenoyl)-sn-glyceryl-3-phosphocholine	2.74	550.3865	POS	C28H56NO7P				0.1252	0.1547	1.1137	0.0100	1.2361
Tetraethylammonium cation	2.74	130.159	POS	C8H20N				0.0093	0.0192	1.3987	0.0000	2.0681
Tetrapropylammonium cation	2.74	186.2215	POS	C12H28N				0.0128	0.0251	2.3115	0.0000	1.9709
Tetradecylamine	2.74	214.2529	POS	C14H31N	HMDB0258887			0.0033	0.0053	1.9288	0.0000	1.5971
1-Hexadecylamine	2.74	242.2841	POS	C16H35N	HMDB0243891			0.0472	0.0783	2.2215	0.0000	1.6599
9,11-Methane-epoxyprostaglandin F1.alpha.	2.74	351.2541	NEG	C21H36O4				0.1602	0.1201	1.1617	0.0019	0.7496
3-Cyclohexyl-2-hydroxypropanoic acid	2.74	171.1028	NEG	C9H16O3				2.0128	1.3672	1.4558	0.0002	0.6793

11-Hydroxyundecanoic acid	2.74	201.1497	NEG	C11H22O3				0.1203	0.0719	1.3542	0.0009	0.5978
Tetraoctylammonium cation	2.74	466.5343	POS	C32H68N				0.0039	0.0065	1.9450	0.0000	1.6491
Dicyclohexyl(hydroxy)acetic acid	2.74	239.1654	NEG	C14H24O3				0.3080	0.2102	1.2163	0.0010	0.6822
(+)-trans-Chrysanthemic acid	2.74	167.1079	NEG	C10H16O2				0.5497	0.3942	1.2693	0.0035	0.7170
2-Cyano-L-phenylalanine	2.74	191.0814	POS	C10H10N2O2				0.0510	0.0246	1.7171	0.0000	0.4834
Dodeca-2(E),4(E)-dienoic acid	2.74	195.1393	NEG	C12H20O2	HMDB0340679			2.9595	2.0419	1.7924	0.0000	0.6900
2,6-Di-tert-butyl-4-(4-morpholinylmethyl)phenol	2.74	306.2426	POS	C19H31NO2				0.1219	1.9295	1.5933	0.0000	15.8232
Nootkatone	2.74	219.1742	POS	C15H22O				0.0522	0.7256	1.8037	0.0001	13.8972
1,5-Naphthalenediamine	2.74	159.0916	POS	C10H10N2	HMDB0244231		C19463	0.0302	0.0381	1.3319	0.0005	1.2640
butabarbital	2.74	211.1089	NEG	C10H16N2O3	HMDB0014382	125-40-6	C07827	0.1428	0.1133	1.4197	0.0191	0.7936
4-Nitrobenzoic acid	2.74	166.0145	NEG	C7H5NO4	HMDB0246532		C18625	0.9814	0.7466	1.3379	0.0110	0.7608
9-HPODE	2.74	311.2231	NEG	C18H32O4	HMDB0062434	29774-12-7	C14827	0.2966	0.1820	1.7983	0.0000	0.6134
6-Pentyl-2H-pyran-2-one	2.74	165.0922	NEG	C10H14O2	HMDB0031085	27593-23-3		2.7491	1.8153	1.2862	0.0026	0.6603
Chlorothalonil-4-hydroxy	2.74	244.9084	NEG	C8HCl3N2O	HMDB0240624	28343-61-5		0.3698	0.2768	1.2162	0.0016	0.7486
LPC(22:6)	2.74	568.3394	POS	C30H50NO7P				0.5460	0.6345	1.2361	0.0162	1.1619
PC(38:5)	2.73	790.5738	POS	C46H82NO8P				0.1789	0.2360	1.3891	0.0120	1.3195
1-Amino-1-cyclobutanecarboxylic acid	2.73	114.0562	NEG	C5H9NO2				3.6298	2.5287	1.3582	0.0002	0.6966
4-Methyl-1H-pyrazole-3-carboxylic acid	2.73	125.0358	NEG	C5H6N2O2				0.0474	0.4155	1.8716	0.0001	8.7698
7H-[1,2,4]Triazolo[4,3-b][1,2,4]triazole-3,7-diamine	2.73	140.068	POS	C3H5N7				0.2652	0.3302	1.4850	0.0005	1.2451
2-Hydroxy-6-methylquinoline-3-carbaldehyde	2.73	188.0704	POS	C11H9NO2				1.2113	1.5695	1.0391	0.0022	1.2957
1-(4-Hydroxyphenyl)-2-methylaminoethanone	2.73	164.0719	NEG	C9H11NO2				5.4730	4.0990	1.5693	0.0002	0.7489
1-O-Octadecyl-sn-glyceryl-3-phosphorylcholine	2.73	510.3914	POS	C26H56NO6P	HMDB0011149	74430-89-0	C04317	0.0834	0.1141	1.6735	0.0003	1.3675
2-(4-Oxo-4H-quinazolin-3-yl)-propanoic acid	2.73	219.0762	POS	C11H10N2O3				0.0057	0.0023	1.5849	0.0001	0.4097
2-((3aR,4S,7R,7aS)-1,3-Dioxohexahydro-1H-4,7-methanoisoindol-2(3H)-yl)propanoic acid	2.73	236.093	NEG	C12H15NO4				0.0888	0.0491	1.4263	0.0004	0.5526
4-(4-Piperidinyl)morpholine	2.73	171.1491	POS	C9H18N2O				0.0243	0.0364	1.1073	0.0000	1.4940
3-Amino-4-ethylbenzenesulfonic acid	2.73	200.0389	NEG	C8H11NO3S				0.0783	0.0556	1.5671	0.0003	0.7099

(3-Methylbutyl)(6-methylheptan-2-yl)amine	2.73	200.2371	POS	C13H29N				0.0019	0.0047	2.0656	0.0000	2.4628
Ciprostene	2.73	363.2543	NEG	C22H36O4				0.4214	0.3094	1.4546	0.0008	0.7343
5-Hydroxy-2-(hydroxymethyl)pyridine	2.73	124.0405	NEG	C6H7NO2				0.1717	0.1209	1.0110	0.0067	0.7038
(12Z)-9,10,11-Trihydroxyoctadec-12-enoic acid	2.73	329.2336	NEG	C18H34O5				0.7307	0.2874	1.8834	0.0000	0.3933
6-Hydroxyoctadec-4-enoic acid	2.73	297.2436	NEG	C18H34O3				0.1104	0.0884	1.2042	0.0058	0.8008
1-Hexadecyl-2-(5Z,8Z,11Z,14Z-eicosatetraenoyl)-sn-glycero-3-phosphocholine	2.73	768.5891	POS	C44H82NO7P				1.4286	1.8682	1.4880	0.0009	1.3077
1-O-Hexadecyl-2-O-(5Z,8Z,11Z,14Z,17Z-eicosapentaenoyl)-sn-glyceryl-3-phosphorylcholine	2.73	766.5738	POS	C44H80NO7P	HMDB0039528	132196-28-2		1.3560	1.7624	1.3721	0.0048	1.2997
2-(3-Methylbutoxy)acetic acid	2.73	145.0871	NEG	C7H14O3				0.2132	0.1575	1.5523	0.0006	0.7388
1-O-Hexadecyl-2-O-(4Z,7Z,10Z,13Z,16Z,19Z-docosahexaenoyl)-sn-glyceryl-3-phosphorylcholine	2.73	792.5894	POS	C46H82NO7P	HMDB0013409	132213-85-5		0.5941	0.7556	1.4131	0.0061	1.2717
1-(1Z-Octadecenyl)-2-(4Z,7Z,10Z,13Z,16Z,19Z-docosahexaenoyl)-sn-glycero-3-phosphocholine	2.73	818.6047	POS	C48H84NO7P	HMDB0011262			0.2355	0.2925	1.4184	0.0216	1.2416
3.beta.,7.alpha.-Dihydroxy-5-cholestenoic acid	2.73	431.3172	NEG	C27H44O4				0.7912	0.5924	1.3978	0.0003	0.7487
N-Methyl-N-(methylsulfonyl)glycine	2.73	166.0181	NEG	C4H9NO4S				3.6399	6.7376	1.3862	0.0005	1.8510
4-(1-Pyrazolyl)benzaldehyde	2.73	173.0707	POS	C10H8N2O				0.0527	0.1039	1.7314	0.0001	1.9707
4-(4-Methyl-1-piperazinyl)butanoic acid	2.73	187.1439	POS	C9H18N2O2				0.0479	0.1604	1.8078	0.0000	3.3470
Dexrazoxane	2.73	267.1102	NEG	C11H16N4O4	HMDB0014524	24584-09-6		0.0873	0.0683	1.0508	0.0155	0.7821
4-Ethoxy-4-oxobut-2-enoic acid	2.73	143.0351	NEG	C6H8O4	HMDB0246416			1.9124	1.4389	1.6779	0.0000	0.7524
Mupirocin	2.73	499.2917	NEG	C26H44O9	HMDB0014554	12650-69-0	C11758	0.2360	0.1490	1.1214	0.0012	0.6314
2,4-dihydroxyheptadec-16-ynyl acetate	2.73	325.2387	NEG	C19H34O4	HMDB0031048	24607-06-5		0.1141	0.0918	1.1847	0.0009	0.8044
1-Octadecylamine	2.73	270.3153	POS	C18H39N	HMDB0029586	124-30-1		0.0611	0.1067	2.2302	0.0000	1.7463
D-Ribose	2.73	149.0457	NEG	C5H10O5	HMDB0000283	613-83-2	C00121	2.1911	1.1685	1.6182	0.0000	0.5333
DIHYDROJASMONIC_ACID	2.73	211.1342	NEG	C12H20O3	HMDB0033601	98674-52-3		1.2716	0.9578	1.3415	0.0001	0.7532
Pyrogallol	2.73	125.0245	NEG	C6H6O3	HMDB0013674	87-66-1	C01108	0.1503	0.1127	1.4229	0.0000	0.7494
LPC(O-18:1)	2.73	508.3759	POS	C26H54NO6P				0.2691	0.3597	1.5349	0.0006	1.3364
LPC(20:4)	2.73	544.3398	POS	C28H50NO7P	HMDB0010395			0.0252	0.0880	1.0849	0.0000	3.4859
Piperolactam A	2.72	266.0816	POS	C16H11NO3	HMDB0033060	112501-42-5		0.0007	0.0028	1.6627	0.0088	4.2217
1-Pyrrolidinecarboximidamide	2.72	114.1025	POS	C5H11N3				0.0081	0.0229	1.0939	0.0045	2.8479

L-Isoserine	2.72	104.0355	NEG	C3H7NO3				0.4608	0.7958	1.4685	0.0000	1.7270
N-[4-(Pyridin-2-yl)-1,3-thiazol-2-yl]cyclopentanecarboxamide	2.72	272.0867	NEG	C14H15N3OS				0.0244	0.0680	1.3683	0.0003	2.7913
2-Amino-5,5,5-trifluoropentanoic acid	2.72	170.0436	NEG	C5H8F3NO2				0.3941	0.3324	1.1205	0.0131	0.8434
(2Z)-2-Hexadecenoic acid	2.72	253.2177	NEG	C16H30O2				0.1353	0.0982	1.0675	0.0029	0.7259
Resorufin	2.72	212.0351	NEG	C12H7NO3	HMDB0257166			0.0118	0.0074	1.5572	0.0332	0.6235
4-Methyloxazole-5-carboxylic acid	2.72	126.0198	NEG	C5H5NO3				0.5462	0.3841	1.3219	0.0001	0.7032
Atropic acid	2.72	147.0454	NEG	C9H8O2				0.2547	0.1909	1.5483	0.0002	0.7494
5-Methyl-1H-pyrazole-3-carbaldehyde	2.72	109.0408	NEG	C5H6N2O				1.3216	0.7188	1.5853	0.0002	0.5439
4-Hydroxytamoxifen	2.72	386.2121	NEG	C26H29NO2	HMDB0060530		C05011	0.0368	0.0697	1.1570	0.0000	1.8950
Hexadecanedioic acid, 3,3,14,14-tetramethyl-	2.72	341.27	NEG	C20H38O4	HMDB0254402			0.1759	0.1217	1.2614	0.0034	0.6918
9S,15S-Dihydroxy-5Z,13E-prostadienoic acid	2.72	337.2387	NEG	C20H34O4				0.1865	0.1321	1.4943	0.0005	0.7086
8-Methoxy-4-oxo-1,4-dihydroquinoline-2-carboxylic acid	2.72	218.0459	NEG	C11H9NO4	HMDB0060426		C05830	0.1127	0.0705	1.1627	0.0029	0.6253
1-Hexadecyl-2-(9Z-octadecenoyl)-sn-glycero-3-phosphocholine	2.72	746.6043	POS	C42H84NO7P				0.3940	0.4420	1.0190	0.0345	1.1217
3-(Aminocarbonyl)benzoic acid	2.72	164.0355	NEG	C8H7NO3				0.2041	0.1376	1.2464	0.0207	0.6739
4,5-DIDEMETHYLSIMMONDSIN	2.72	344.1352	NEG	C15H23NO8	HMDB0041207	135074-86-1		0.1294	0.0899	1.6869	0.0000	0.6950
LPC(O-22:1)	2.72	564.4389	POS	C30H62NO6P				0.0147	0.0203	1.1010	0.0035	1.3861
Eicoseneoylcarnitine (Car(20:1))	2.72	454.3896	POS	C27H51NO4				0.0070	0.0105	1.5670	0.0018	1.5049
Piperazine-N,N'-bis(2-hydroxypropanesulfonic acid)	2.71	363.0898	POS	C10H22N2O8S2				0.0310	0.0211	1.1496	0.0061	0.6821
DL-2-Methylglutamic acid	2.71	160.0617	NEG	C6H11NO4				0.4135	0.3302	1.4115	0.0026	0.7987
1-Methylhydantoin	2.71	115.0503	POS	C4H6N2O2	HMDB0003646	616-04-6	C02565	0.0017	0.0512	1.7596	0.0006	29.5578
1-Oleoyl-2-palmitoyl-sn-glycero-3-phosphocholine	2.71	760.5832	POS	C42H82NO8P	HMDB0008100			0.2173	0.3453	1.5203	0.0041	1.5888
15S-Hydroperoxy-11Z,13E-eicosadienoic acid	2.71	339.2545	NEG	C20H36O4				0.1814	0.1329	1.3306	0.0008	0.7323
Urushiol I	2.71	319.2645	NEG	C21H36O2				0.0923	0.0720	1.1165	0.0207	0.7801
Trihydroxycholestanoic acid	2.71	449.3281	NEG	C27H46O5	HMDB0000601	547-98-8		0.3869	0.2722	1.2260	0.0008	0.7036
3-Dodecyloxypropylamine	2.71	244.2632	POS	C15H33NO				0.0167	0.0250	2.1903	0.0000	1.4960
2,5-Dimethyl-4-pyrimidinamine	2.71	124.0868	POS	C6H9N3				0.0156	0.0082	1.3946	0.0004	0.5289

(3E,5E)-2-(4-Methoxy-4-oxobutyl)nona-3,5-dienoic acid	2.71	253.1447	NEG	C14H22O4				0.1219	0.0842	1.4136	0.0002	0.6911
5.alpha.-Pregnan-3.alpha.,17-diol-20-one 3-sulfate	2.71	413.2009	NEG	C21H34O6S				6.2815	4.1888	1.2531	0.0007	0.6668
2-Methylpropanamine	2.71	74.0964	POS	C4H11N	HMDB0034198	78-81-9	C02787	0.8213	2.8073	1.9195	0.0000	3.4182
(S)-3,4-Dihydroxybutyric acid (lithium hydrate)	2.71	119.0351	NEG	C4H8O4	HMDB0000337	51267-44-8		2.0820	1.7992	1.0981	0.0008	0.8642
4-Hydroxyglutamic acid	2.71	144.0305	NEG	C5H9NO5	HMDB0002273	2485-33-8	C05947	0.2934	0.1568	1.7596	0.0001	0.5346
Isovaleramide	2.71	102.0911	POS	C5H11NO		541-46-8	D04637	0.0703	0.3402	1.9361	0.0000	4.8380
PI(16:0/18:1)	2.71	835.5325	NEG	C43H81O13P	HMDB0009783	50730-13-7	C13888	1.3080	0.8495	1.2309	0.0001	0.6494
2-Hydroxy-1,3-dimethyl-9H-thioxanthen-9-one	2.7	255.0478	NEG	C15H12O2S				0.0099	0.2159	1.7395	0.0009	21.8835
Pro-Pro	2.7	213.1235	POS	C10H16N2O3	HMDB0011180	20488-28-2		0.0263	0.0103	1.7156	0.0000	0.3913
2-Amino-4,4,4-trifluorobutanoic acid	2.7	156.0278	NEG	C4H6F3NO2				0.2087	0.2660	1.0045	0.0001	1.2742
His-Thr	2.7	257.1243	POS	C10H16N4O4	HMDB0028895	142879-28-5		0.0059	0.0039	1.2940	0.0084	0.6724
Pro-Asp	2.7	229.0832	NEG	C9H14N2O5				0.2088	0.1264	1.1112	0.0243	0.6052
2-(4-Amino-1-piperidinyl)acetamide	2.7	158.1288	POS	C7H15N3O				0.0892	0.0657	1.4143	0.0002	0.7366
5-((1E,3E)-Hepta-1,3-dien-1-yl)-1,2,3-trihydroxycyclopentanecarboxylic acid	2.7	255.1245	NEG	C13H20O5				0.4267	0.3265	1.2557	0.0035	0.7650
N-Methyldioctylamine	2.7	256.2998	POS	C17H37N				0.0012	0.0025	1.0545	0.0000	2.0712
Palmitoyleicosapentaenoyl phosphatidylcholine	2.7	780.5519	POS	C44H78NO8P				1.1171	1.4173	1.3171	0.0016	1.2687
(2-{[2-docosanamido-3-hydroxyoctadec-4-en-1-yl phosphonato]oxy}ethyl)trimethylazanium	2.7	845.6711	NEG	C45H91N2O6P	HMDB0012103	94359-12-3		0.1806	0.1325	1.0173	0.0011	0.7336
PC(36:5)	2.7	780.552	POS	C44H78NO8P	HMDB0007890			0.0233	0.0346	1.5275	0.0088	1.4867
PC(38:7)	2.7	804.5517	POS	C46H78NO8P				0.0299	0.0350	1.1977	0.0010	1.1701
Thr-Glu	2.69	247.0937	NEG	C9H16N2O6				0.0045	0.5221	1.8044	0.0003	115.0797
2-(Naphthalen-1-yl)-1H-1,3-benzodiazole	2.69	245.1065	POS	C17H12N2				0.0586	0.0403	1.0953	0.0058	0.6868
Diisopropyl_sulfide	2.69	119.0893	POS	C6H14S	HMDB0029579	625-80-9		0.2354	0.2773	1.1049	0.0043	1.1780
Arg-Gly-Asp	2.69	347.1682	POS	C12H22N6O6	HMDB0248572			0.0031	0.0144	1.7615	0.0000	4.5933
cis-4-Hydroxycyclohexanecarboxylic acid	2.69	143.0715	NEG	C7H12O3				0.1245	0.0909	1.5404	0.0000	0.7304
1-Hexadecyl-2-(8Z,11Z,14Z-eicosatrienoyl)-sn-glycero-3-phosphocholine	2.69	770.6029	POS	C44H84NO7P				0.4667	0.5601	1.1561	0.0131	1.2002
9-Hydroxyrisperidone	2.69	425.2009	NEG	C23H27FN4O3	HMDB0015396	144598-75-4		0.4104	0.2686	1.0012	0.0234	0.6543

5-Isopropyl-5-methylhydantoin	2.68	157.0971	POS	C7H12N2O2				0.0186	0.0103	1.7251	0.0000	0.5530
2-Amino-3-(1H-pyrazol-1-yl)propanoic acid	2.68	156.0767	POS	C6H9N3O2	HMDB0034267	2734-48-7		0.0567	0.2488	1.1989	0.0000	4.3891
1,11-Undecanedicarboxylic acid	2.68	243.1604	NEG	C13H24O4	HMDB0002327	505-52-2		0.0755	0.0491	1.2173	0.0183	0.6502
1,2-Distearoyl-sn-glycero-3-phospho-(1'-rac-glycerol)	2.68	777.5683	NEG	C42H83O10P				0.0798	0.0588	1.0502	0.0092	0.7366
Meclizine	2.68	391.1944	POS	C25H27ClN2	HMDB0014875	569-65-3	C07116	0.0013	0.0045	1.7491	0.0000	3.4337
LPC(O-20:0)	2.68	538.4245	POS	C28H60NO6P				0.0141	0.0180	1.3844	0.0055	1.2778
2-(Chloromethyl)-5,6-dimethylthieno[2,3-d]pyrimidin-4-ol	2.67	227.0052	NEG	C9H9ClN2OS				0.9343	0.5936	1.2128	0.0041	0.6354
Valdecoxib	2.67	315.0798	POS	C16H14N2O3S	HMDB0005033	181695-72-7		0.0972	2.9250	1.7492	0.0004	30.0900
4-Methyl-3-(trifluoromethyl)-1H-pyrazol-5-amine	2.67	166.0579	POS	C5H6F3N3				0.0099	0.0146	1.1106	0.0088	1.4813
5-Aminopentanal	2.67	102.0913	POS	C5H11NO	HMDB0012815		C12455	0.0632	0.0323	1.7956	0.0001	0.5118
1-Behenoyl-2-hydroxy-sn-glycero-3-phosphocholine	2.67	580.4334	POS	C30H62NO7P	HMDB0010398		C04230	0.0257	0.0326	1.1004	0.0100	1.2705
D-SEDOHEPTULOSE	2.67	209.0677	NEG	C7H14O7	HMDB0254327			0.2496	0.3844	1.5043	0.0000	1.5401
6-Thioinosine	2.67	283.0519	NEG	C10H12N4O4S				0.8465	0.6978	1.2809	0.0155	0.8243
3-Hydroxy-2-[(Z)-oct-2-enyl]pentanedioic acid	2.67	257.1396	NEG	C13H22O5				0.3467	0.2718	1.3890	0.0011	0.7839
Thelephoric acid	2.67	351.0125	NEG	C18H8O8	HMDB0030552	479-64-1		0.1144	0.4182	1.7879	0.0000	3.6565
Suberohydroxamic acid	2.66	203.1039	NEG	C8H16N2O4				0.0068	0.0834	1.8289	0.0000	12.2151
2-(Aminomethyl)-4-chlorophenol	2.66	156.023	NEG	C7H8ClNO				0.0074	0.3241	1.6983	0.0026	43.7294
2-Oct-7-enylpentanedioic acid	2.66	241.1447	NEG	C13H22O4				0.0509	0.0355	1.3149	0.0006	0.6969
Arachidonic sulfonic acid	2.66	339.2	NEG	C19H32O3S				11.0525	8.7894	1.0246	0.0176	0.7952
3-Hydroxyglutaric acid	2.65	147.0301	NEG	C5H8O5	HMDB0000428	638-18-6		0.2315	0.1354	1.1123	0.0014	0.5849
4-(Pentafluorosulfanyl)aniline	2.65	220.0201	POS	C6H6F5NS				0.0063	0.0110	1.6758	0.0004	1.7470
5-(2-Fluorobenzyl)-1,3-thiazol-2-amine	2.65	209.0532	POS	C10H9FN2S				0.0026	0.1873	1.8675	0.0000	72.7102
2-(1-Methyl-1H-pyrazol-4-yl)-1H-benzimidazole	2.65	197.0822	NEG	C11H10N4				0.0297	0.0228	1.1869	0.0169	0.7663
Phaclofen	2.65	248.0236	NEG	C9H13ClNO3P	HMDB0256382			0.4849	0.2015	1.3170	0.0000	0.4155
Hymexazole	2.65	100.0392	POS	C4H5NO2				0.0184	0.0116	1.2230	0.0001	0.6305
5-Bromo-4-fluoro-2-hydroxybenzoic acid	2.65	232.9251	NEG	C7H4BrFO3				0.0720	0.0556	1.2038	0.0014	0.7720

Artocarpin	2.65	437.1937	POS	C26H28O6	HMDB0030849	7608-44-8		0.0033	0.0040	1.0960	0.0050	1.2316
FA 18:3+2O	2.65	309.2073	NEG	C18H30O4	HMDB0246689			0.1625	0.1139	1.4826	0.0001	0.7008
LPC(19:0)	2.65	538.3861	POS	C27H56NO7P		108273-88-7		0.0552	0.0787	1.7263	0.0000	1.4273
N6-(1-Iminoethyl)-L-lysine	2.64	188.1394	POS	C8H17N3O2	HMDB0249982			0.0291	0.0161	1.1608	0.0371	0.5543
(.+/-.)-Nuarimol	2.64	315.0676	POS	C17H12ClFN2O				0.0545	3.6656	1.7596	0.0004	67.2445
4-Chloro-6-propylamino-2-methylthiopyrimidine	2.64	218.051	POS	C8H12ClN3S				0.0021	0.0052	1.4104	0.0080	2.4509
2-Naphthalenecarboxamide, N-[2-(4-oxo-1-phenyl-1,3,8-triazaspiro[4.5]dec-8-yl)ethyl]-	2.64	427.2165	NEG	C26H28N4O2				0.4676	0.3253	1.2628	0.0035	0.6956
2-({2-[(2-Carboxyphenyl)sulfanyl]ethyl}sulfanyl)benzoic acid	2.63	333.0237	NEG	C16H14O4S2				0.0093	0.3911	1.9030	0.0000	42.0222
2-Cyano-N-(3,4-difluorophenyl)acetamide	2.63	197.0532	POS	C9H6F2N2O				0.0151	0.3132	1.6352	0.0004	20.8060
(2E)-4-Hydroxybut-2-enoic acid	2.63	101.0245	NEG	C4H6O3	HMDB0003381	24587-49-3		2.9869	2.1834	1.7074	0.0000	0.7310
o-Nitrobenzoic acid	2.63	166.0146	NEG	C7H5NO4	HMDB0245255		C16234	0.6214	0.3142	1.5740	0.0000	0.5055
4-{[4-(Acetyloxy)benzoyl]amino}benzoic acid	2.63	298.07	NEG	C16H13NO5				1.2709	0.6970	1.6140	0.0019	0.5484
4-Amino-2-oxo-1,2-dihydropyrimidine-5-carboxylic acid	2.63	154.026	NEG	C5H5N3O3	HMDB0246202			2.4242	1.7342	1.5685	0.0023	0.7153
Heptadecanoyl_carnitine	2.63	414.3578	POS	C24H47NO4	HMDB0006210	106182-29-0		0.0075	0.0098	1.1803	0.0006	1.3186
PC(40:9)	2.63	828.5493	POS	C48H78NO8P				0.0498	0.0686	1.8256	0.0000	1.3774
LPA(0:0/18:2(9Z,12Z))	2.62	435.2506	POS	C21H39O7P	HMDB0007852			0.0016	0.0026	1.2907	0.0000	1.6391
4-Chloro-N-(2-methoxy-5-methylphenyl)benzenesulfonamide	2.62	310.0328	NEG	C14H14ClNO3S				0.1486	0.4349	1.3740	0.0000	2.9261
1-(3-(Trifluoromethyl)phenyl)piperazine	2.62	231.1087	POS	C11H13F3N2	HMDB0244703			0.0019	0.0060	1.3016	0.0001	3.1128
5,10-Dihydro-10-phenophosphazinol 10-oxide	2.61	230.0396	NEG	C12H10NO2P				0.1718	0.1500	1.5423	0.0010	0.8734
Damnacanthal	2.61	283.0624	POS	C16H10O5	HMDB0250841			0.0334	0.0223	1.1910	0.0035	0.6673
L-Arginine ethyl ester	2.61	203.1501	POS	C8H18N4O2				0.0082	0.0191	1.0907	0.0263	2.3273
6-Fluoro-2-(trifluoromethyl)-4-quinolinol	2.61	230.0253	NEG	C10H5F4NO				0.2574	0.1335	1.7365	0.0000	0.5186
monolinolein	2.61	393.24	POS	C21H38O4	HMDB0242115			0.0008	0.0016	1.6129	0.0000	2.0054
3,5-Dihydroxyphenylglycine	2.61	182.046	NEG	C8H9NO4	HMDB0244908			0.1339	0.0931	1.1476	0.0006	0.6951
PC(18:1)	2.6	518.3219	POS	C26H50NO8P		56391-91-4		1.1562	1.5014	1.6164	0.0000	1.2986
3-{[(4-Fluorophenyl)sulfanyl]methyl}-1-benzofuran-2-carboxylic acid	2.6	301.0352	NEG	C16H11FO3S				0.0016	0.0070	1.7827	0.0000	4.5002

2,2,5,5-Tetramethyl-2,5-dihydro-1H-imidazole-4-thiol	2.6	159.0957	POS	C7H14N2S				0.0064	0.0245	1.4461	0.0125	3.8096
N-Acetylglycylglycine	2.6	173.057	NEG	C6H10N2O4				0.2297	1.5720	1.4738	0.0034	6.8442
1-(2,4-Dihydroxyphenyl)-2-(4-nitrophenyl)ethanone	2.6	272.0542	NEG	C14H11NO5				0.1568	0.0960	1.3387	0.0084	0.6125
5-(4-Acetoxy-3-oxo-1-butynyl)-2,2'-bithiophene	2.6	291.0122	POS	C14H10O3S2	HMDB0038459	1222-83-9		0.0143	0.0906	1.0339	0.0061	6.3488
Alloimperatorin	2.6	271.0925	POS	C16H14O4	HMDB0301848		C09053	0.0372	0.0453	1.2266	0.0430	1.2157
4,4-Difluoro-1-phenyl-1,3-butanedione	2.59	199.0574	POS	C10H8F2O2				0.0022	0.0031	1.1053	0.0092	1.4112
Prolyl-Histidine	2.59	253.128	POS	C11H16N4O3	HMDB0029019			0.0043	0.0118	1.7210	0.0004	2.7532
Gln-Glu	2.58	274.1049	NEG	C10H17N3O6				0.0270	0.0535	1.4850	0.0000	1.9832
4-Methyl-N-(4-methyl-2-pyridinyl)benzenesulfonamide	2.58	263.0874	POS	C13H14N2O2S				0.0080	0.0421	1.1770	0.0006	5.2563
Vanilpyruvic_acid	2.58	211.0593	POS	C10H10O5	HMDB0011714	1081-71-6		0.0005	0.0016	1.2620	0.0345	3.0384
1,6-Dihydroxy-3,7-dimethoxy-2-(2-hydroxy-3-methyl-3-butenyl)-8-(3-methyl-2-butenyl)-xanthone	2.58	441.1887	POS	C25H28O7	HMDB0039915			0.0158	0.0225	1.3237	0.0105	1.4288
Dihydrocitrinone	2.57	265.0692	NEG	C13H14O6				0.2530	0.1177	1.8430	0.0000	0.4654
rac-4-Sulfoxypropranolol	2.57	354.1041	NEG	C16H21NO6S				0.0176	0.0123	1.3594	0.0019	0.7009
1-Methyl-3-isobutylxanthine	2.57	223.1175	POS	C10H14N4O2	HMDB0245912		C13708	0.0066	0.0214	1.6068	0.0052	3.2307
Nodakenitin	2.57	247.0926	POS	C14H14O4	HMDB0302268		C09278	0.0019	0.0649	1.5607	0.0025	34.0713
1h-indole-3-butanoic acid	2.57	204.0979	POS	C12H13NO2	HMDB0002096	133-32-4	C11284	0.0360	0.2623	1.4780	0.0014	7.2948
6-Nitro-1,2,3-benzotriazin-4(3H)-one	2.56	191.0201	NEG	C7H4N4O3				0.9231	5.1918	1.8654	0.0000	5.6244
4-Allyl-5-(2-methylphenyl)-4H-1,2,4-triazol-3-yl hydrosulfide	2.56	232.0929	POS	C12H13N3S				0.0008	0.0219	1.7441	0.0000	25.8410
(2R)-2-(2,5-Difluorophenyl)pyrrolidine	2.56	184.0943	POS	C10H11F2N				0.7480	0.8519	1.1064	0.0080	1.1389
1H-Indazole-3-carboxamide, 1-(5-fluoropentyl)-N-8-quinolinyl-	2.56	377.1785	POS	C22H21FN4O				0.0009	0.0078	1.7846	0.0000	8.4432
1-Hydroxy-2-naphthoic acid	2.56	187.0421	NEG	C11H8O3	HMDB0243892		C03203	6.3007	5.1829	1.2556	0.0225	0.8226
PC(22:4(7Z,10Z,13Z,16Z)/P-18:1(9Z))	2.56	820.6224	POS	C48H86NO7P	HMDB0008655		C00157	0.0529	0.0628	1.0516	0.0191	1.1868
DG(18:1(9Z)/18:4(6Z,9Z,12Z,15Z)/0:0)	2.56	615.4966	POS	C39H66O5	HMDB0007222			0.0085	0.0107	1.1065	0.0023	1.2576
5,6,7,8-Tetrahydrothieno[2,3-b]quinolin-4-amine	2.55	205.0819	POS	C11H12N2S				0.0023	0.0557	1.8061	0.0001	24.0541
3-Cyano-4,7-dimethylcoumarin	2.55	200.0682	POS	C12H9NO2				0.0176	0.0913	1.6528	0.0000	5.1939
Methanone, (4-ethyl-1-naphthalenyl)(5-hydroxy-1-pentyl-1H-indol-3-yl)-	2.55	386.2075	POS	C26H27NO2				0.0040	0.0235	1.8796	0.0000	5.8346

threo-Syringoylglycerol	2.55	245.0996	POS	C11H16O6	HMDB0031237	121748-11-6	0.0276	0.1126	1.5750	0.0076	4.0854
Methyl 1-hydroxy-2-naphthoate	2.55	201.0578	NEG	C12H10O3			0.5083	0.3677	1.0087	0.0253	0.7235
5-(4-Aminophenyl)-4-phenyl-2,4-dihydro-3H-1,2,4-triazole-3-thione	2.55	267.0735	NEG	C14H12N4S			0.8834	0.5230	1.4124	0.0073	0.5920
N-(2-Thienylmethyl)-7H-purin-6-amine	2.55	232.0637	POS	C10H9N5S			0.0059	0.0030	1.0026	0.0332	0.5116
N-Docosanoyltaurine	2.55	446.3362	NEG	C24H49NO4S			2.1531	1.6724	1.2828	0.0020	0.7768
3-(5-Amino-3-methyl-1H-pyrazol-1-yl)propanenitrile	2.55	151.0964	POS	C7H10N4			0.0606	0.0725	1.0276	0.0155	1.1959
16,16-Dimethylprostaglandin A2	2.55	361.2363	NEG	C22H34O4			0.5492	0.4102	1.5140	0.0002	0.7469
Triptophenolide	2.55	311.1689	NEG	C20H24O3			20.8088	15.5062	1.1919	0.0125	0.7452
8-Geranyl-7-hydroxycoumarin	2.55	297.1532	NEG	C19H22O3			8.0499	5.8361	1.2205	0.0110	0.7250
Pyrazolam	2.54	354.0305	POS	C16H12BrN5			0.0029	0.0164	1.7889	0.0000	5.5971
2,5-Dimethyl-3,4-diphenyl-2,4-cyclopentadien-1-one	2.54	261.1306	POS	C19H16O			0.0189	0.0228	1.5390	0.0001	1.2045
Triciribine	2.54	321.1311	POS	C13H16N6O4			0.0103	0.0142	1.4401	0.0014	1.3784
1,6-Anhydro-.beta.-D-glucose	2.54	161.0457	NEG	C6H10O5	HMDB0000640	498-07-7	3.5341	2.6041	1.8274	0.0000	0.7368
2-Bromo-4,6-dichlorophenol	2.53	238.8704	NEG	C6H3BrCl2O			0.2020	0.1073	1.6982	0.0002	0.5311
4-(1,3-Dithiolan-2-yl)aniline	2.53	198.0381	POS	C9H11NS2			0.0049	0.0215	1.7061	0.0002	4.3731
5-(2-methoxyethylamino)-3H-1,3,4-thiadiazole-2-thione	2.53	192.0244	POS	C5H9N3OS2			0.0095	0.0171	1.3345	0.0000	1.8040
6-Hydrazino-3-pyridazinecarboxamide	2.53	154.0743	POS	C5H7N5O			0.0313	0.1036	1.5708	0.0002	3.3090
Benz[a]anthracen-1-ol, 8-methoxy-3-methyl-	2.53	289.1258	POS	C20H16O2			0.0092	0.0606	1.6912	0.0002	6.5627
8beta-Angeloyloxy-15-hydroxy-1alpha,10R-dimethoxy-3-oxo-11(13)-germacren-12,6alpha-olide	2.53	425.2149	POS	C22H32O8	HMDB0039013		0.0054	0.0067	1.2548	0.0027	1.2456
Gln-Arg	2.53	301.1592	NEG	C11H22N6O4			0.1706	0.1211	1.2435	0.0041	0.7098
1-(1H-Indol-3-yl)-2-(4-morpholinyl)-2-oxoethanone	2.53	257.0965	NEG	C14H14N2O3			0.0750	0.0173	1.1648	0.0000	0.2300
5-Amino-2-(5-amino-1,3-benzoxazol-2-yl)phenol	2.53	240.075	NEG	C13H11N3O2			0.1491	0.0701	1.3727	0.0032	0.4703
3-Hydroxy-3-(methoxycarbonyl)pentanedioic acid	2.53	205.0355	NEG	C7H10O7			0.2946	0.1766	1.1760	0.0162	0.5994
1-Allyl-6-hydroxy-2,4(1H,3H)-pyrimidinedione	2.52	167.0439	NEG	C7H8N2O3			0.2496	0.2287	1.3257	0.0207	0.9164
Endothal dimethyl ester	2.52	215.089	POS	C10H14O5			0.0130	0.0328	1.4197	0.0037	2.5181
1H-Pyrrole-2,5-dione, 1-methyl-3-(1-methyl-1H-indol-3-yl)-4-(pentylamino)-	2.52	326.1904	POS	C19H23N3O2			0.0008	0.0030	1.3968	0.0067	3.7372

Arachidyl_carnitine	2.52	456.4052	POS	C27H53NO4	HMDB0006460			0.0032	0.0062	1.6965	0.0001	1.9776
N-[4-Nitro-3-(trifluoromethyl)phenyl]acetamide	2.51	249.0458	POS	C9H7F3N2O3				0.0004	0.0090	1.5327	0.0000	20.9763
5-(Trifluoromethyl)-2(1H)-pyridinone	2.51	164.0294	POS	C6H4F3NO				0.0442	0.0763	1.4073	0.0003	1.7275
Ikarugamycin	2.51	477.282	NEG	C29H38N2O4				0.5121	0.4187	1.0301	0.0462	0.8175
1-(4-Chlorophenyl)-2-[(5-methyl-4H-1,2,4-triazol-3-yl)sulfanyl]ethanone	2.51	266.0179	NEG	C11H10ClN3OS				45.6486	24.3673	1.4970	0.0002	0.5338
Acetyl tributyl citrate	2.51	403.2329	POS	C20H34O8	HMDB0034159	77-90-7		0.0251	0.0358	1.8525	0.0001	1.4243
Desisopropyldisopyramide	2.5	298.1872	POS	C18H23N3O	HMDB0061024			0.0100	0.0122	1.1077	0.0004	1.2207
2-(3-Carboxy-4-chlorophenyl)-1,3-dioxo-2,3-dihydro-1H-isoindole-5-carboxylic acid	2.5	343.9952	NEG	C16H8ClNO6				0.7024	3.4891	1.5622	0.0004	4.9673
Altenuene	2.5	291.0837	NEG	C15H16O6				0.9607	96.4211	1.8097	0.0000	100.3638
Fenarimol	2.5	331.0449	POS	C17H12Cl2N2O	HMDB0040599		C11226	0.1247	5.8648	1.8508	0.0000	47.0418
2-(4-Morpholinyl)-4H-pyrimido[2,1-a]isoquinolin-4-one	2.5	282.1195	POS	C16H15N3O2				0.1194	0.1360	1.3666	0.0076	1.1393
3H-Benzimidazol-5-yl-[4-[(5-cyclopropyl-1H-pyrazol-3-yl)amino]pyrimidin-2-yl]amine	2.5	333.1522	POS	C17H16N8				0.0030	0.0285	1.8016	0.0000	9.6226
1-O-Hexadecyl-2-O-(N-methylcarbamoyl)-sn-glyceryl-3-phosphorylcholine	2.5	539.39	POS	C26H55N2O7P				0.0162	0.0223	1.5394	0.0000	1.3757
10-Methylbenzo[g]pteridine-2,4(3H,10H)-dione	2.5	227.054	NEG	C11H8N4O2				0.1015	0.0655	1.0093	0.0063	0.6450
5-(4-Methylpiperazin-1-yl)-5-oxopentanoic acid	2.5	215.1388	POS	C10H18N2O3				0.0259	0.0491	1.3138	0.0010	1.8966
Risedronic_acid	2.5	284.0091	POS	C7H11NO7P2	HMDB0015022	105462-24-6	C08233	0.0470	0.0959	1.4864	0.0000	2.0389
3-(3-Hydroxy-5-methylphenoxy)-5-methylbenzene-1,2-diol	2.49	245.0782	NEG	C14H14O4				0.0828	0.0694	1.2170	0.0285	0.8383
2-[1-(Aminomethyl)cyclopentyl]acetic acid	2.49	158.1174	POS	C8H15NO2				0.0143	0.0262	1.6321	0.0000	1.8309
Heptanedioic acid, 1-(2-cyclopentylidenehydrazide)	2.48	241.1547	POS	C12H20N2O3				0.0591	0.0699	1.0011	0.0023	1.1820
N-Stearoyltaurine	2.48	390.2735	NEG	C20H41NO4S	HMDB0240595	63155-80-6		0.0998	0.0827	1.0082	0.0430	0.8287
2-Methyl-3-[(tetrahydro-2-furanylcarbonyl)amino]benzoic acid	2.47	248.0929	NEG	C13H15NO4				0.1299	0.0653	1.6991	0.0004	0.5026
7-Hydroxy-6-methoxy-3-methyl-5-(propan-2-ylidene)furo[2,3,4-de]chromen-2(5H)-one	2.46	273.0732	NEG	C15H14O5				0.0033	0.4256	1.8325	0.0001	129.2923
N-.alpha.-(tert-Butoxycarbonyl)-L-proline	2.46	214.1085	NEG	C10H17NO4				0.3170	0.1442	1.6933	0.0000	0.4549
PC(20:2(11Z,14Z)/18:3(9Z,12Z,15Z))	2.46	808.5828	POS	C46H82NO8P	HMDB0008338			0.2044	0.2504	1.5516	0.0001	1.2255
PC(18:0/18:3(6Z,9Z,12Z))	2.46	784.5819	POS	C44H82NO8P	HMDB0008040			8.2381	10.0409	1.5115	0.0037	1.2188
4-[5-(7-Hydroxy-5,5,8a-trimethyl-2-methylidene-3,4,4a,6,7,8	2.46	407.2806	NEG	C24H40O5				0.3210	0.2533	1.0032	0.0084	0.7891

-hexahydro-1H-naphthalen-1-yl)-3-methylpentoxy]-4-oxobutanoic acid												
PC(20:2(11Z,14Z)/20:5(5Z,8Z,11Z,14Z,17Z))	2.45	832.5835	POS	C48H82NO8P	HMDB0008347			0.1092	0.1318	1.5846	0.0014	1.2075
Xanthophyll	2.45	568.427	POS	C40H56O2	HMDB0303013			0.2186	0.2463	1.1529	0.0125	1.1267
Fluazifop	2.44	328.0821	POS	C15H12F3NO4	HMDB0252322		C18527	0.0002	0.0173	1.7854	0.0000	97.4928
Harderoporphyrinogen	2.44	615.315	POS	C35H42N4O6	HMDB0002160	42607-18-1		0.0016	0.0070	1.2240	0.0008	4.3326
2-Chloro-4,5-dimethoxybenzoic acid	2.44	215.0145	NEG	C9H9ClO4				1.4076	0.7434	1.7546	0.0000	0.5281
Oxfendazole	2.44	314.0558	NEG	C15H13N3O3S	HMDB0031812	53716-50-0		0.0449	0.0235	1.4186	0.0003	0.5235
(E)-5-[(1S,4aR,8aR)-2-Formyl-5,5,8a-trimethyl-1,4,4a,6	2.43	375.2212	NEG	C22H32O5				0.9087	0.6705	1.4469	0.0001	0.7378
,7,8-hexahydronaphthalen-1-yl]-3-(acetyloxymethyl)pent-2-enoic acid												
Mebendazole	2.43	294.0912	NEG	C16H13N3O3	HMDB0014781	31431-39-7		0.0015	0.1637	1.5344	0.0001	110.1578
PC(P-18:1(9Z)/0:0)	2.43	506.3601	POS	C26H52NO6P	HMDB0010408		C04230	0.0510	0.0744	1.5851	0.0000	1.4587
Amphetamine	2.42	158.0924	POS	C9H13N	HMDB0014328	300-62-9	C07514	0.0021	0.0161	1.8967	0.0000	7.6982
DL-2,4-Diaminobutyric acid	2.41	119.0814	POS	C4H10N2O2	HMDB0002362	305-62-4		0.2060	0.5651	1.6350	0.0006	2.7438
Nilutamide	2.41	318.0681	POS	C12H10F3N3O4	HMDB0014803	63612-50-0	C08164	0.0012	0.0455	1.7869	0.0002	37.9659
LysoPC(22:1(13Z))	2.41	578.4181	POS	C30H60NO7P	HMDB0010399		C04230	0.0075	0.0175	1.1576	0.0000	2.3402
5-(Tetradecyloxy)-2-furoic acid	2.4	323.2232	NEG	C19H32O4				0.0814	0.0625	1.3249	0.0002	0.7683
PC(18:1(9Z)/18:3(9Z,12Z,15Z))	2.39	782.5693	POS	C44H80NO8P	HMDB0008074			0.1121	0.1678	1.7622	0.0017	1.4975
3-[(1E,3E)-Hepta-1,3-dienyl]pentanedioic acid	2.39	225.1133	NEG	C12H18O4				0.4214	0.2877	1.5211	0.0000	0.6827
PC(22:5(7Z,10Z,13Z,16Z,19Z)/20:4(8Z,11Z,14Z,17Z))	2.39	856.5825	POS	C50H82NO8P	HMDB0008707		C00157	0.0428	0.0537	1.5473	0.0012	1.2541
(2-aminoethoxy)[2-[hexadec-9-enoyloxy]-3-[octadeca-1.11-dien-1-yloxy]propoxy]phosphinic acid	2.38	698.5133	NEG	C39H74NO7P	HMDB0011405			5.6665	3.3509	1.2582	0.0001	0.5913
L-Erythrulose	2.38	119.0351	NEG	C4H8O4	HMDB0006293	533-50-6	C02045	0.7026	0.3097	1.5987	0.0000	0.4407
1-Hydroxypyrene	2.37	219.0829	POS	C16H10O	HMDB0013139	5315-79-7	C14519	0.0143	0.0105	1.3737	0.0014	0.7313
2-(2,6-Dihydroxy-4-methoxycarbonylbenzoyl)-3-hydroxybenzoic acid	2.37	331.0504	NEG	C16H12O8				0.0844	0.0671	1.7637	0.0002	0.7957
LysoPC(16:1(9Z))	2.37	494.3247	POS	C24H48NO7P	HMDB0010383	76790-27-7	C04230	0.9020	1.1658	1.1970	0.0014	1.2925
9,10-Dihydrojasmonic acid	2.37	211.1341	NEG	C12H20O3				0.2760	0.1759	1.4631	0.0005	0.6372
PC(20:2(11Z,14Z)/18:1(9Z))	2.37	812.6161	POS	C46H86NO8P	HMDB0008334			0.0380	0.0820	1.1530	0.0400	2.1557

PC(20:3(5Z,8Z,11Z)/14:0)	2.37	756.5536	POS	C42H78NO8P	HMDB0008361			1.4406	1.7179	1.0914	0.0263	1.1925
PC(20:3(5Z,8Z,11Z)/P-18:0)	2.37	796.62	POS	C46H86NO7P	HMDB0008391			0.8882	0.9847	1.1206	0.0462	1.1087
3-cis-Hydroxy-b,e-Caroten-3'-one	2.37	551.4241	POS	C40H54O	HMDB0002890	143167-27-5		0.2437	0.2936	1.3268	0.0142	1.2048
Niflumic acid	2.36	281.0545	NEG	C13H9F3N2O2	HMDB0015573	4394-00-7	C13698	2.5902	2.1587	1.2373	0.0285	0.8334
(1-Amino-3-methyl-1,3-dihydrobenzoimidazol-2-ylidene)cyanoacetic acid ethyl ester	2.35	259.1153	POS	C13H14N4O2				0.0173	0.0639	1.5926	0.0004	3.6941
PC(14:0/P-18:1(9Z))	2.35	716.5566	POS	C40H78NO7P	HMDB0007898		C00157	0.0141	0.0201	1.0880	0.0067	1.4274
Oseltamivir acid	2.34	283.1663	NEG	C14H24N2O4				0.0479	0.0228	1.1262	0.0061	0.4767
2-(Dipentylamino)-2-(hydroxymethyl)-1,3-propanediol	2.34	262.2375	POS	C14H31NO3				0.0260	0.0097	2.2056	0.0000	0.3717
honokiol	2.34	267.1439	POS	C18H18O2	HMDB0253212		C10630	0.0049	0.0221	1.6557	0.0003	4.5003
PC(22:4(7Z,10Z,13Z,16Z)/P-18:0)	2.34	822.636	POS	C48H88NO7P	HMDB0008653			0.2409	0.2819	1.3202	0.0063	1.1703
2,6-Cyclolycopene-1,5-diol	2.33	570.44	POS	C40H58O2	HMDB0034902	223744-29-4		0.0448	0.0714	1.7477	0.0000	1.5958
1-(10Z-Heptadecenoyl)-sn-glycero-3-phospho-(1'-rac-glycerol)	2.33	495.2793	NEG	C23H45O9P				0.2583	0.1861	1.1487	0.0009	0.7203
Demethoxycurcumin	2.33	337.1113	NEG	C20H18O5	HMDB0033801	22608-11-3		0.1033	0.0630	1.7270	0.0001	0.6095
4-tert-Butyl-N-(4-nitrophenyl)benzamide	2.32	299.139	POS	C17H18N2O3				0.0057	0.0138	1.3893	0.0002	2.4475
Nitrotyrosine	2.31	225.0495	NEG	C9H10N2O5	HMDB0001904	3604-79-3		0.0081	0.0230	1.4411	0.0004	2.8514
(3-Oxo-2-piperazinyl)acetic acid	2.31	159.0764	POS	C6H10N2O3				0.0022	0.0063	1.6683	0.0000	2.8460
3-Amino-5,6-dimethyl-2-pyrazinecarboxylic acid	2.3	168.0767	POS	C7H9N3O2				0.0160	0.2098	1.7861	0.0000	13.1501
3-Hydroxy-6-methylheptyl 2-O-.beta.-D-glucopyranosyl-.beta.-D-glucopyranoside	2.3	469.2271	NEG	C20H38O12				0.0911	0.0609	1.3582	0.0010	0.6685
PI(16:1(9Z)/18:1(9Z))	2.29	835.533	POS	C43H79O13P	HMDB0009801		C00626	0.0120	0.0083	1.0295	0.0007	0.6931
1-Anilino-3-(3,6-dibromo-9H-carbazol-9-yl)-2-propanol	2.26	472.9858	POS	C21H18Br2N2O				0.0009	0.0051	1.6599	0.0001	5.6173
2-Hexylbutanedioic acid	2.26	201.1135	NEG	C10H18O4				0.0566	0.0446	1.2593	0.0162	0.7870
N-(4-Methylphenyl)-N'-(6-methyl-2-pyridinyl)thiourea	2.26	256.0912	NEG	C14H15N3S				0.0162	0.0076	1.0058	0.0002	0.4703
Okaramine D	2.26	581.2409	NEG	C33H34N4O6				0.0653	0.0554	1.2480	0.0207	0.8484
CPA(18:2(9Z,12Z)/0:0)	2.26	417.2397	POS	C21H37O6P	HMDB0007007			0.0007	0.0013	1.3364	0.0000	1.7447
Eplerenone	2.26	415.2116	POS	C24H30O6	HMDB0014838	107724-20-9	C12512	0.0024	0.0034	1.3433	0.0005	1.3857
Erythronolactone	2.26	117.0195	NEG	C4H6O4	HMDB0000349	17675-99-9		0.8519	0.5941	1.6444	0.0005	0.6973

Nitrofurazone	2.26	197.0316	NEG	C6H6N4O4	HMDB0014480	59-87-0	C08042	178.9130	128.7669	1.5809	0.0045	0.7197
3-Oxocyclobutanecarboxylic acid	2.25	113.0246	NEG	C5H6O3				2.8816	2.1514	1.6191	0.0000	0.7466
N-Acetylglucosaminylasparagine	2.25	334.1258	NEG	C12H21N3O8				0.1064	0.8729	1.2232	0.0000	8.2031
5-[(2-Chlorophenoxy)methyl]-4-methyl-4H-1,2,4-triazole-3-thiol	2.25	254.0159	NEG	C10H10ClN3OS				0.0813	0.0442	1.0355	0.0462	0.5441
(E)-2-Cyano-1-(tert-pentyl)guanidine	2.25	155.1291	POS	C7H14N4				0.0237	0.0170	1.2025	0.0004	0.7180
(2S)-2-[(2R,3S,7R,8R,8aS)-2,3,4'-Trihydroxy-4,4,7,8a-tetramethyl -6'-oxospiro[2,3,4a,5,6,7-hexahydro-1H-naphthalene-8,2 '-3,8-dihydrofuro[2,3-e]isoindole]-7'-yl]-3-methylbutanoic acid	2.25	502.2799	POS	C28H39NO7				0.0018	0.0066	1.5923	0.0000	3.7573
4-(4-Morpholinyl)-1,3,5-triazin-2-ylamine	2.25	182.1036	POS	C7H11N5O				0.0220	0.0193	1.0154	0.0253	0.8745
(R)-N-[1-[4-(2,4-Dimethoxybenzyl)-5-phenethyl-4H-1 ,2,4-triazol-3-yl]-2-(1H-indol-3-yl)ethyl]picolinamide	2.25	587.2766	POS	C35H34N6O3	HMDB0253735			0.0171	0.0692	1.3798	0.0000	4.0574
N-Cyclohexyl-N'-(tetrahydro-2-furanylmethyl)urea	2.25	227.1753	POS	C12H22N2O2				0.0279	0.0354	1.0349	0.0067	1.2696
Methoxyfenozide	2.25	369.2122	POS	C22H28N2O3	HMDB0254547		C18525	0.0009	0.0037	1.8972	0.0000	4.0353
Putative Phenylalanine conjugated chenodeoxycholic acid	2.25	540.3663	POS	C33H49NO5	HMDB0242391			0.0294	0.0391	1.4977	0.0001	1.3310
ISOPALMITIC ACID	2.25	255.233	NEG	C16H32O2	HMDB0031068	4669-02-7		2.7204	2.2933	1.1648	0.0169	0.8430
Lormetazepam	2.24	335.0378	POS	C16H12Cl2N2O2	HMDB0041919	848-75-9		0.0024	0.1252	1.8651	0.0000	51.9290
4-(3-Methyl-5-oxo-4,5-dihydro-1H-pyrazol-1-yl)benzoic acid	2.24	217.0618	NEG	C11H10N2O3				0.1519	0.0787	1.5716	0.0001	0.5179
N1-[3-(Benzyloxy)benzyl]-1H-tetrazole-1,5-diamine	2.24	297.1477	POS	C15H16N6O				0.0138	0.0296	1.0341	0.0002	2.1389
13,14-dihydro-PGE1	2.24	357.2651	POS	C20H36O5	HMDB0002689	19313-28-1		0.0021	0.0013	1.0351	0.0319	0.6353
2-{[(2-Oxo-2H-chromen-3-yl)carbonyl]amino}benzoic acid	2.23	310.0705	POS	C17H11NO5				0.0003	0.0064	1.1223	0.0009	19.7568
N-Phenyl-N'-(3-pyridinylmethyl)thiourea	2.23	242.076	NEG	C13H13N3S				0.0046	0.0035	1.3401	0.0026	0.7640
5-[2-Chloro-5-(trifluoromethyl)anilino]-5-oxopentanoic acid	2.23	308.0311	NEG	C12H11ClF3NO3				0.1735	0.1399	1.0919	0.0125	0.8063
Emodin-8-Beta-D-Glucoside	2.23	471.0695	POS	C21H20O10	HMDB0303798			0.0005	0.0007	1.0113	0.0216	1.4513
Streptozocin	2.23	266.0976	POS	C8H15N3O7	HMDB0014572	18883-66-4	C07313	0.1757	0.1961	1.0875	0.0088	1.1160
Bromhexine	2.22	375.0056	POS	C14H20Br2N2				0.0009	0.0020	1.1905	0.0162	2.2883
Taxifolin	2.22	303.0504	NEG	C15H12O7	HMDB0303943		C01617	0.0464	0.0258	1.0864	0.0025	0.5568

(2-aminoethoxy)[2-[octadec-9-enoyloxy]-3-[octadeca-1 .11-dien-1-yloxy]propoxy]phosphinic acid	2.22	726.5441	NEG	C41H78NO7P	HMDB0011408			7.0918	3.9526	1.4517	0.0000	0.5573
SM_C16:1	2.22	716.5776	POS	C40H80N2O6P	HMDB0029216			0.0525	0.0629	1.3411	0.0234	1.1970
1-[6-Chloro-5-(trifluoromethoxy)-1H-benzimidazol-2-yl]-1H-pyrazole-4-carboxylic acid	2.21	347.0127	POS	C12H6ClF3N4O3				0.0047	0.0424	1.5336	0.0004	9.0471
N-Acetylsulfamethoxazole	2.21	296.0659	POS	C12H13N3O4S	HMDB0013854	21312-10-7	C13061	0.0077	0.0275	1.6085	0.0000	3.5617
Neohancoside D	2.21	547.1694	NEG	C23H32O15				0.2546	0.1692	1.6890	0.0000	0.6647
2-[(2-Fluorophenyl)amino]acetohydrazide	2.21	182.0762	NEG	C8H10FN3O				0.2422	0.1958	1.4134	0.0009	0.8086
2-amino-3-({hydroxy[2-(icosanoyloxy)-3-[octadec-11-enoyloxy]propoxy]phosphoryl}oxy)propanoic acid	2.21	816.5764	NEG	C44H84NO10P	HMDB0112392			49.7330	41.5500	1.0207	0.0076	0.8355
2,4-DIHYDROXYPTERIDINE	2.21	163.0251	NEG	C6H4N4O2	HMDB0254198		C03212	0.0803	0.0491	1.6755	0.0003	0.6109
S-Sulforaphene	2.21	176.1393	POS	C6H9NOS2	HMDB0031573	2404-46-8		0.0262	0.0373	1.4247	0.0000	1.4214
Convallatoxin	2.21	551.2867	POS	C29H42O10	HMDB0034362	5822-57-1		0.0015	0.0043	1.6086	0.0000	2.7876
7-Hydroxy-3-(4-methoxyphenoxy)-2-(trifluoromethyl)-4H-chromen-4-one	2.2	351.0497	NEG	C17H11F3O5				0.0781	0.1084	1.0087	0.0332	1.3874
Fenazaflor	2.2	374.9922	POS	C15H7Cl2F3N2O2				0.0010	0.0033	1.2375	0.0020	3.1772
3-[[(5-Methyl-1,3,4-thiadiazol-2-yl)thio]methyl]coumarilic acid	2.2	307.0195	POS	C13H10N2O3S2				0.0015	0.0021	1.2011	0.0005	1.4269
trans-Epoxysuccinyl-L-leucylamido(4-guanidino)butane	2.2	358.2072	POS	C15H27N5O5				0.0073	0.0143	1.3438	0.0088	1.9561
Aurintricarboxylic acid	2.2	421.0604	NEG	C22H14O9	HMDB0248731			1.2942	0.7617	1.0406	0.0115	0.5885
Fenhexamid	2.2	300.0576	NEG	C14H17Cl2NO2	HMDB0252201		C18593	0.0982	0.0683	1.6845	0.0000	0.6963
(22E,24R)-Stigmasta-4,22-diene-3,6-dione	2.2	425.3456	POS	C29H44O2	HMDB0038656	50868-51-4		0.0993	0.1203	1.0422	0.0058	1.2112
arctiin	2.19	533.2051	NEG	C27H34O11	HMDB0301809	20362-31-6	C16915	0.0119	0.0067	1.5110	0.0029	0.5628
Linalool_oxide_D_3-[apiosyl-(1->6)-glucoside]	2.19	465.2306	POS	C21H36O11	HMDB0031367			0.0002	0.0010	1.3944	0.0003	4.1115
Benzophenone-4	2.19	307.0299	NEG	C14H12O6S	HMDB0258614			0.6498	0.4897	1.2839	0.0019	0.7536
Sulprophos sulfoxide	2.18	339.0342	POS	C12H19O3PS3				0.0033	0.0118	1.7272	0.0008	3.5650
Lys-His	2.18	284.1704	POS	C12H21N5O3				0.0040	0.0180	1.6065	0.0005	4.5473
Benazepril	2.18	423.1957	NEG	C24H28N2O5	HMDB0014682	86541-75-5	C06843	0.0198	0.0125	1.6286	0.0032	0.6349
Ethyl 3-(trifluoromethyl)-1H-pyrazole-5-carboxylate	2.17	207.0366	NEG	C7H7F3N2O2				0.0039	0.0249	1.7853	0.0000	6.3695

Chicoric acid	2.17	473.0747	NEG	C22H18O12	HMDB0002375	70831-56-0	C10437	0.0954	0.0761	1.7862	0.0012	0.7978
Dopamine 4-.beta.-D-glucuronide	2.17	328.1075	NEG	C14H19NO8				2.0903	1.6784	1.1036	0.0006	0.8029
N-(4-Chlorobenzyl)-9H-purin-6-amine	2.17	258.0566	NEG	C12H10ClN5				0.1594	0.1257	1.1944	0.0125	0.7888
apigetrin	2.17	431.1007	NEG	C21H20O10	HMDB0037340	578-74-5	C04608	0.0167	0.0113	1.3430	0.0183	0.6780
3-Hydroxystigmast-5-en-7-one	2.17	429.3772	POS	C29H48O2	HMDB0034422	2034-74-4		0.0231	0.0384	1.7929	0.0000	1.6586
5-Azacytidine	2.16	243.0753	NEG	C8H12N4O5				0.0044	0.0029	1.1571	0.0015	0.6630
His-Tyr	2.16	319.1365	POS	C15H18N4O4	HMDB0028897	35979-00-1		0.0066	0.0190	1.5918	0.0005	2.8743
1,7-Dioxa-4,10-diazacyclododecane	2.15	175.143	POS	C8H18N2O2				0.0983	0.1728	1.4391	0.0009	1.7574
4-Benzyloxy-3-iodo-5-methoxybenzaldehyde	2.15	369.0005	POS	C15H13IO3				0.0004	0.0092	1.8438	0.0000	21.1761
Cirsimaritin	2.15	313.0738	NEG	C17H14O6	HMDB0250276		C17785	0.0477	0.0021	1.5220	0.0002	0.0447
{4-[(Phenoxyacetyl)amino]phenyl}acetic acid	2.15	284.091	NEG	C16H15NO4				0.0512	0.0857	1.1604	0.0000	1.6754
Gallomyricitrin	2.14	615.1033	NEG	C28H24O16				0.0322	0.0209	1.0792	0.0125	0.6511
3-(3-Nitrophenyl)-5-(2-phenylethyl)-1,2,4-oxadiazole	2.13	296.1051	POS	C16H13N3O3				0.0003	0.0353	1.7705	0.0008	136.7837
Epalrestat	2.13	320.0387	POS	C15H13NO3S2				0.0069	0.0257	1.0653	0.0010	3.7461
Sulfamethoxazole	2.13	252.0466	NEG	C10H11N3O3S	HMDB0015150	723-46-6	C07315	0.0574	0.0434	1.2355	0.0016	0.7561
Gentamicin	2.13	478.3285	POS	C21H43N5O7	HMDB0014936	1403-66-3		0.0191	0.0276	1.1715	0.0010	1.4418
2-(2-Bromo-4,5-dimethoxyphenyl)-6,7-dimethoxy-1-naphthol	2.12	417.0387	NEG	C20H19BrO5				0.0222	0.0311	1.3017	0.0026	1.3998
Methyl 2-hydroxy-3,5-dinitrobenzoate	2.12	241.0122	NEG	C8H6N2O7				0.0730	0.0503	1.0322	0.0285	0.6882
liriodenine	2.12	298.0526	POS	C17H9NO3	HMDB0301844		C09567	0.0057	0.0098	1.4154	0.0000	1.7240
Florfenicol	2.12	358.0105	POS	C12H14Cl2FNO4S	HMDB0252314			0.0006	0.0498	1.7621	0.0000	87.6896
4-Bromo-1-(4-morpholinosulfonylphenyl)pyrazole	2.11	371.9975	POS	C13H14BrN3O3S				0.0126	0.0493	1.0807	0.0061	3.9242
Phe-His	2.11	303.1417	POS	C15H18N4O3	HMDB0028997	33367-37-2		0.0074	0.0317	1.6372	0.0003	4.2809
Fusarochromanone	2.11	293.1528	POS	C15H20N2O4	HMDB0033514	104653-89-6		0.0007	0.0010	1.0724	0.0052	1.4436
4H-1,2,4-Triazole-3-sulfonamide	2.1	146.9968	NEG	C2H4N4O2S				0.1278	0.0905	1.0564	0.0014	0.7081
(1S)-2-Amino-1-(4-chlorophenyl)-1-[4-(1H-pyrazol-4-yl)phenyl]ethanol	2.09	312.094	NEG	C17H16ClN3O				0.0151	0.0554	1.6614	0.0000	3.6627
5-Hydroxy-2-{[(4-nitrophenoxy)acetyl]amino}benzoic acid	2.09	331.061	NEG	C15H12N2O7				0.2130	0.1666	1.3830	0.0001	0.7823

Propyl gallate	2.08	211.0589	NEG	C10H12O5	HMDB0033835	121-79-9	C11155	0.4927	0.3972	1.2520	0.0030	0.8062
1-(4-Chlorophenyl)-3,5-dimethyl-1H-pyrazole-4-carboxylic acid	2.07	249.0471	NEG	C12H11ClN2O2				0.1661	0.1434	1.6143	0.0029	0.8636
5'-Hydroxytenoxicam	2.07	354.0257	POS	C13H11N3O5S2	HMDB0014098			0.0002	0.0012	1.4309	0.0001	5.9237
Corynanthin	2.07	372.223	POS	C21H26N2O3	HMDB0250478			0.0043	0.0316	1.8590	0.0000	7.3315
Serine-Cholic Acid	2.07	460.3116	POS	C27H45NO7	HMDB0242388			0.0074	0.0422	1.8295	0.0003	5.7446
4-Imidazolidineheptanoic acid, 3-[(2-cyclohexyl-2-hydroxyethyl)amino]-2,5-dioxo-1-(phenylmethyl)-	2.06	460.2752	POS	C25H37N3O5	HMDB0249489			0.0022	0.0099	1.7947	0.0001	4.5073
Swertiamarin	2.06	419.1145	NEG	C16H22O10	HMDB0258644			0.0531	0.0308	1.6616	0.0001	0.5795
Lanceoloside A	2.05	391.1082	NEG	C19H20O9				0.0452	0.0303	1.4762	0.0176	0.6694
(1,3-Benzothiazol-2-ylsulfonyl)acetic acid	2.05	255.9713	NEG	C9H7NO4S2				0.0462	0.1367	1.6718	0.0000	2.9549
Mebeverine	2.05	430.2649	POS	C25H35NO5	HMDB0254388			0.0052	0.0320	1.8773	0.0000	6.1598
Procaterol	2.04	291.1665	POS	C16H22N2O3	HMDB0015453	72332-33-3		0.0013	0.0032	1.2041	0.0076	2.4440
Aspochalasin D	2.02	402.2696	POS	C24H35NO4				0.0061	0.0456	1.7046	0.0003	7.4286
gamma-Amino-gamma-cyanobutanoate	1.99	129.0659	POS	C5H8N2O2				0.1760	1.9425	1.7232	0.0005	11.0393
N-Acetylneuraminate	1.95	308.0989	NEG	C11H19NO9	HMDB0000230	131-48-6	C00270	9.5199	7.4239	1.5576	0.0120	0.7798
(R)-S-Lactoylglutathione	1.94	416.053	NEG	C13H21N3O8S				0.2005	0.1595	1.3829	0.0008	0.7955
M324T174	1.94	324.094	NEG	C9H15NO8				0.9489	0.6848	1.7067	0.0000	0.7217
(2S)-2-Isopropyl-3-oxosuccinate	1.9	173.0458	NEG	C7H10O5				0.1678	0.0336	1.1312	0.0030	0.2003
M211T175	1.89	211.0014	NEG	C5H11O8P				0.0467	0.0363	1.1837	0.0169	0.7785
M243T127	1.88	243.018	NEG	C6H12O8S				0.0529	0.0373	1.0143	0.0032	0.7052
Ethanolamine phosphate	1.87	200.0333	NEG	C2H8NO4P	HMDB0000224	1071-23-4	C00346	0.0513	0.0740	1.1804	0.0015	1.4424
4-Guanidinobutanal	1.87	130.0974	POS	C5H11N3O				0.6471	0.4422	1.4796	0.0001	0.6834
5-Hydroxyisourate	1.87	165.0055	NEG	C5H4N4O4				1.2624	0.9622	1.3773	0.0096	0.7622
M513T53	1.87	513.4167	NEG	C28H54O4				0.1922	0.1290	1.0584	0.0022	0.6711
Sebacic acid	1.85	201.1133	NEG	C10H18O4	HMDB0000792	111-20-6	C08277	0.0929	0.0585	1.4837	0.0001	0.6297
UDP-N-acetyl-D-mannosaminouronate	1.85	637.0809	NEG	C17H25N3O18P2				0.0294	0.0220	1.5044	0.0110	0.7488
UDP-3-ketoglucose	1.84	580.0593	NEG	C15H22N2O17P2				0.1050	0.0781	1.3976	0.0002	0.7438

Formamidopyrimidine nucleoside triphosphate	1.82	579.9702	POS	C10H18N5O15P3				0.2265	0.2529	1.3583	0.0048	1.1166
5-Hydroxy-2-oxo-4-ureido-2,5-dihydro-1H-imidazole-5-carboxylate	1.81	201.0257	NEG	C5H6N4O5				0.0110	0.0344	1.2029	0.0073	3.1366
M255T209	1.81	254.9827	POS	C6H8O10S				0.2396	0.2756	1.7016	0.0001	1.1504
M87T134	1.81	87.009	NEG	C3H4O3				0.2851	0.2237	1.4722	0.0263	0.7844
P1,P4-Bis(5'-uridyl) tetraphosphate	1.79	849.0102	NEG	C18H26N4O23P4				0.0067	0.0040	1.4563	0.0021	0.5973
M213T111_1	1.79	213.0173	NEG	C7H12O5				1.5255	0.7737	1.6588	0.0000	0.5072
M246T207	1.78	246.0324	POS	C5H8O8S				0.0092	0.0161	1.3286	0.0003	1.7599
1-(5'-Phosphoribosyl)-5-formamido-4-imidazolecarboxamide	1.77	349.0552	POS	C10H15N4O9P				0.0004	0.0491	1.5945	0.0002	124.5226
L-2-Amino-3-oxobutanoic acid	1.77	98.0249	NEG	C4H7NO3				3.8062	2.4008	1.5337	0.0274	0.6308
M282T176	1.77	282.083	NEG	C9H14O9				0.0517	0.4077	1.5759	0.0027	7.8857
3-Sulfino-L-alanine	1.76	212.0292	NEG	C3H7NO4S				0.0027	0.0111	1.6803	0.0010	4.1720
M505T253	1.76	505.0407	NEG	C10H16N4O12P2				0.0033	0.1013	1.7335	0.0005	30.4497
2,5-Dioxopiperazine	1.75	95.0252	NEG	C4H6N2O2				0.1216	0.0686	1.6472	0.0007	0.5639
M209T209	1.75	209.0044	NEG	C7H8O6				0.4632	0.3857	1.0376	0.0253	0.8327
UDP	1.74	403.0002	NEG	C9H14N2O12P2	HMDB0000295	58-98-0	C00015	0.1421	0.2435	1.5974	0.0007	1.7134
2-Aminobut-2-enoate	1.74	137.9964	NEG	C4H7NO2				0.0680	0.0748	1.0833	0.0385	1.0991
D-Glucosaminate-6-phosphate	1.73	334.05	NEG	C6H14NO9P				0.1598	0.1138	1.1508	0.0002	0.7121
M642T230	1.73	641.9981	NEG	C18H24NO18PS				0.0051	0.0035	1.3099	0.0115	0.6801
3-Sulfinylpyruvate	1.71	172.9581	NEG	C3H4O5S				0.1846	0.1354	1.1759	0.0115	0.7335
N-Feruloylglycine	1.71	272.0539	NEG	C12H13NO5				0.7219	0.4292	1.6334	0.0035	0.5945
M202T112	1.71	202.0382	NEG	C7H6O6				0.7396	0.5973	1.2925	0.0043	0.8075
4-Carboxy-4-hydroxy-2-oxoadipate	1.7	256.966	NEG	C7H8O8				0.0980	0.2918	1.6622	0.0000	2.9763
2-Hydroxy-2,4-pentadienoate	1.7	113.0246	NEG	C5H6O3				1.4424	0.8689	1.5494	0.0001	0.6024
M149T198	1.69	149.0459	NEG	C5H10O5				0.1065	0.0691	1.7049	0.0023	0.6489
GDP-glucose	1.65	586.0666	NEG	C16H25N5O16P2				0.0066	0.0054	1.1068	0.0120	0.8102
M708T231	1.65	707.9698	NEG	C15H24N5O19P3				0.0034	0.0059	1.5651	0.0006	1.7097

Pyrimidine 5'-nucleotide	1.64	309.0683	NEG	C9H14N2O7P				0.1933	0.1726	1.3439	0.0191	0.8931
Methylimidazole acetaldehyde	1.62	125.0709	POS	C6H8N2O				0.0803	0.0486	1.0203	0.0063	0.6053
Nicotinamide D-ribonucleotide	1.61	315.0419	NEG	C11H15N2O8P				0.8215	2.2330	1.6431	0.0003	2.7180
M347T238	1.61	347.0035	NEG	C10H14O11				0.2507	1.6914	1.1515	0.0027	6.7456
M643T213	1.6	642.9811	NEG	C15H22N2O20P2S				0.0605	0.0457	1.0848	0.0012	0.7555
M401T210	1.58	401.0426	NEG	C12H20O14S				0.1179	0.0937	1.0040	0.0050	0.7942
M228T85_2	1.58	228.0283	NEG	C10H9NO4				0.4754	0.2592	1.7461	0.0000	0.5452
Imidazole-4-acetate	1.57	144.0768	POS	C5H6N2O2	HMDB0002024	645-65-8	C02835	0.0101	0.0611	1.5168	0.0007	6.0318
N2-Succinyl-L-arginine	1.53	255.1079	NEG	C10H18N4O5				0.2664	0.1717	1.6194	0.0004	0.6445
M455T209	1.52	455.0141	NEG	C12H19O15P				1.5328	1.1971	1.4283	0.0045	0.7810
M573T85	1.5	573.395	NEG	C30H53O9				0.1471	0.1027	1.4728	0.0004	0.6981
N5-Phenyl-L-glutamine	1.47	221.0933	NEG	C11H14N2O3				0.0475	0.0196	1.4839	0.0000	0.4118
M132T202	1.47	132.0669	NEG	C5H11NO3				0.0162	0.0123	1.3173	0.0035	0.7575
M186T205	1.46	186.0177	NEG	C5H11NO4				0.0079	0.0188	1.6719	0.0014	2.3812
D-Glycerate	1.45	144.9847	POS	C3H6O4				0.0563	0.0653	1.0386	0.0243	1.1603
M213T89_1	1.44	213.0175	NEG	C7H12O5				4.1252	2.4241	1.7024	0.0001	0.5876
3-Cyano-L-alanine	1.43	153.0061	POS	C4H6N2O2				0.0001	0.0016	1.7432	0.0001	13.2861
3-Indoleacetonitrile	1.42	177.0407	NEG	C10H8N2				1.1754	0.6620	1.5662	0.0000	0.5632
M577T46	1.33	577.3752	NEG	C33H54O8				0.6573	0.4633	1.0507	0.0055	0.7048
2-Aminomalonate semialdehyde	1.31	84.0053	NEG	C3H5NO3				0.0394	0.0313	1.2951	0.0358	0.7944
(3S)-2-Oxo-3-phenylbutanoate	1.31	199.0381	NEG	C10H10O3				0.2366	0.2061	1.1330	0.0319	0.8713
4-Hydroxy-L-glutamate	1.3	179.0666	NEG	C5H9NO5				0.1004	0.0741	1.6074	0.0000	0.7377
Lithocholic acid	1.29	435.312	NEG	C24H40O3	HMDB0000761	434-13-9	C03990	1.0748	0.7851	1.1703	0.0037	0.7305
4-Aminobutyraldehyde	1.28	175.1439	POS	C4H9NO				0.0044	0.0214	1.4060	0.0006	4.8835
N-Succinyl-L-glutamate 5-semialdehyde	1.26	290.0885	NEG	C9H13NO6				0.0127	0.0080	1.3366	0.0052	0.6301
Cellobionate	1.25	357.1095	NEG	C12H22O12				0.0371	0.0225	1.4635	0.0000	0.6063

2-Chlorobenzoate	1.25	172.0205	NEG	C7H5ClO2			0.0186	0.0114	1.7386	0.0001	0.6117
M243T162_1	1.24	243.0625	NEG	C9H9NO6			0.0507	0.0411	1.1084	0.0041	0.8099
Oxamate	1.19	109.9919	NEG	C2H3NO3	471-47-6	C01444	0.0180	0.0137	1.3437	0.0319	0.7611
M305T170	1.12	305.0392	NEG	C10H14N2O7S			0.0293	0.0229	1.1316	0.0043	0.7822

Table S2. KEGG Enrichment data matrix before and after a 12-week BBr60 intervention on serum metabolism

**Pathway**	**Description**	**Compounds.(dem)**	**Percent**	**Rich_factor**	**p_value**	**up_nums**	**down_nums**	**DA_score**
hsa05230	Central carbon metabolism in cancer	C00152;C00065;C00025;C00122;C00407;C00078;C00183;C00022;C00158;C00049;C00149;C00311	11.76470588	0.324324324	1.21578E-11	8	4	0.333333333
hsa04974	Protein digestion and absorption	C00152;C00065;C00025;C00407;C00078;C00099;C00183;C00047;C08262;C00049;C00246	10.78431373	0.234042553	4.53213E-09	6	5	0.090909091
hsa01230	Biosynthesis of amino acids	C00152;C00327;C00065;C00025;C00407;C00263;C00078;C00233;C00183;C00022;C00047;C00141;C00979;C00158;C00049;C00311	15.68627451	0.125	1.93E-08	10	6	0.25
hsa00630	Glyoxylate and dicarboxylate metabolism	C00065;C00025;C00417;C00022;C00158;C00160;C00168;C00209;C00149;C00311	9.803921569	0.15625	1.35367E-06	6	4	0.2
hsa00770	Pantothenate and CoA biosynthesis	C00864;C00099;C00183;C00022;C00141;C00049;C00429	6.862745098	0.233333333	3.5023E-06	1	6	-0.714285714
hsa00020	Citrate cycle (TCA cycle)	C00122;C00417;C00022;C00158;C00149;C00311	5.882352941	0.3	3.67694E-06	4	2	0.333333333
hsa01200	Carbon metabolism	C00065;C00025;C00122;C00022;C00979;C00158;C00160;C00168;C00049;C00149;C00311;C00257	11.76470588	0.107142857	7.00E-06	7	5	0.166666667
hsa00290	Valine, leucine and isoleucine biosynthesis	C00407;C00233;C00183;C00022;C02226;C00141	5.882352941	0.260869565	9.05721E-06	0	6	-1
hsa00260	Glycine, serine and threonine metabolism	C00065;C00719;C00263;C00078;C00022;C00168;C00049;C00546	7.843137255	0.166666667	1.00095E-05	5	3	0.25
hsa00970	Aminoacyl-tRNA biosynthesis	C00152;C00065;C00025;C00407;C00078;C00183;C00047;C0004	7.843137255	0.153846154	1.85091E-05	6	2	0.5

		9						
hsa01210	2-Oxocarboxylic acid metabolism	C00025;C00417;C00407;C00078;C00233;C00183;C00022;C00047;C02226;C00141;C00158;C00049;C00311	12.74509804	0.090277778	1.88892E-05	7	6	0.076923077
hsa00250	Alanine, aspartate and glutamate metabolism	C00152;C00025;C00122;C00022;C00158;C00049	5.882352941	0.214285714	3.08065E-05	4	2	0.333333333
hsa04978	Mineral absorption	C00152;C00065;C00407;C00078;C00183;C00009	5.882352941	0.206896552	3.81292E-05	4	2	0.333333333
hsa02010	ABC transporters	C00065;C00025;C00245;C00719;C00407;C00294;C00183;C00047;C00049;C00009;C01606;C00121	11.76470588	0.086956522	5.89042E-05	6	6	0
hsa00410	beta-Alanine metabolism	C00864;C00099;C01073;C00383;C00049;C00429	5.882352941	0.1875	6.88042E-05	1	5	-0.666666667
hsa00360	Phenylalanine metabolism	C00122;C00022;C00642;C11457;C07086;C05593;C01198	6.862745098	0.142857143	0.000103478	0	7	-1
hsa04922	Glucagon signaling pathway	C00122;C00022;C00158;C00149;C00311	4.901960784	0.192307692	0.000253759	3	2	0.2
hsa00591	Linoleic acid metabolism	C14766;C14762;C06426;C00157;C14827	4.901960784	0.178571429	0.000365614	1	4	-0.6
hsa00232	Caffeine metabolism	C13747;C07480;C07130;C16361	3.921568627	0.181818182	0.001370458	1	3	-0.5
hsa05211	Renal cell carcinoma	C00122;C00149	1.960784314	0.666666667	0.001528787	1	1	0
hsa00220	Arginine biosynthesis	C00327;C00025;C00122;C00049	3.921568627	0.173913043	0.001630116	3	1	0.5
hsa05231	Choline metabolism in cancer	C00588;C00157;C04230	2.941176471	0.272727273	0.001673735	3	0	1
hsa00280	Valine, leucine and isoleucine degradation	C00407;C00233;C00183;C00141;C02170	4.901960784	0.119047619	0.002456367	0	5	-1
hsa00240	Pyrimidine metabolism	C02067;C00099;C00383;C02170;C00429;C00015	5.882352941	0.09375	0.003154118	1	5	-0.666666667
hsa00270	Cysteine and methionine metabolism	C00065;C00263;C00022;C00979;C03145;C00049	5.882352941	0.089552239	0.00397337	3	3	0
hsa00650	Butanoate metabolism	C00025;C00122;C00022;C00246;C00741	4.901960784	0.106382979	0.004047687	1	4	-0.6
hsa00330	Arginine and proline metabolism	C02946;C00025;C00791;C00022;C02565;C05947	5.882352941	0.086956522	0.004600999	2	4	-0.333333333
hsa00620	Pyruvate metabolism	C00122;C00022;C00149;C00546	3.921568627	0.125	0.005655607	1	3	-0.5
hsa01100	Metabolic pathways	C00152;C13747;C07480;C00327;C02946;C00065;C07130;C00025;C00122;C00864;C04555;C02341;C00417;C00245;C00719;C00407;C00294;C00263;C05570;C00366;C00078;C02067;C00791;C06423;C00233;C00099;C00183;C00022;C00047;C00642;C02226;C06427;C06337;C11457;C00141;C05578;C01571;C06426;C0058	80.39215686	0.026675342	0.005947198	25	57	-0.390243902

		8;C00979;C00158;C00383;C00180;C00160;C00168;C01717;C01114;C00209;C00049;C07086;C00314;C00149;C00311;C05593;C02170;C00246;C00257;C06578;C02378;C01744;C03765;C08972;C00486;C01198;C10700;C00546;C00429;C01606;C00157;C14827;C04317;C00121;C02565;C05947;C12455;C03203;C01617;C00270;C00346;C00015;C02835;C01444						
hsa04976	Bile secretion	C04555;C05465;C00366;C07185;C06804;C00486;C03990	6.862745098	0.072164948	0.006323254	2	5	-0.428571429
hsa04080	Neuroactive ligand-receptor interaction	C00025;C00245;C00099;C00049;C00015	4.901960784	0.094339623	0.006798125	3	2	0.2
hsa00920	Sulfur metabolism	C00065;C00245;C00263;C00979	3.921568627	0.117647059	0.007043927	2	2	0
hsa00350	Tyrosine metabolism	C00122;C00022;C00642;C05578;C05593;C03765	5.882352941	0.076923077	0.008360454	0	6	-1
hsa00564	Glycerophospholipid metabolism	C00065;C00588;C00157;C04230;C00346	4.901960784	0.089285714	0.008574818	5	0	1
hsa01240	Biosynthesis of cofactors	C00065;C00025;C00864;C00078;C06423;C00099;C00183;C00022;C00141;C00158;C00049;C00314;C00311;C00015	13.7254902	0.042682927	0.016148657	7	7	0
hsa00430	Taurine and hypotaurine metabolism	C00025;C00245;C00022	2.941176471	0.125	0.016555811	1	2	-0.333333333
hsa00785	Lipoic acid metabolism	C06423;C00233;C00022;C00141	3.921568627	0.090909091	0.017333642	0	4	-1
hsa00470	D-Amino acid metabolism	C00065;C00025;C00022;C00047;C00049	4.901960784	0.072463768	0.019999034	4	1	0.6
hsa00340	Histidine metabolism	C00025;C05570;C00049;C02835	3.921568627	0.085106383	0.021624659	4	0	1
hsa00190	Oxidative phosphorylation	C00122;C00009	1.960784314	0.166666667	0.029405942	1	1	0
hsa04742	Taste transduction	C00025;C00158;C00149	2.941176471	0.09375	0.035591953	3	0	1
hsa04071	Sphingolipid signaling pathway	C00065;C00346	1.960784314	0.133333333	0.044755505	2	0	1
hsa00030	Pentose phosphate pathway	C00022;C00257;C00121	2.941176471	0.081081081	0.05140534	1	2	-0.333333333
hsa04964	Proximal tubule bicarbonate reclamation	C00025;C00149	1.960784314	0.117647059	0.056290232	2	0	1
hsa00640	Propanoate metabolism	C00099;C02170;C00546	2.941176471	0.073170732	0.066109887	0	3	-1
hsa04723	Retrograde endocannabinoid signaling	C00025;C00157	1.960784314	0.105263158	0.068734586	2	0	1
hsa00563	Glycosylphosphatidylinositol (GPI)-anchor biosynthesis	C00346	0.980392157	0.25	0.088360496	1	0	1

hsa04068	FoxO signaling pathway	C00025	0.980392157	0.2	0.109214326	1	0	1
hsa03320	PPAR signaling pathway	C14762	0.980392157	0.2	0.109214326	0	1	-1
hsa05016	Huntington disease	C00025	0.980392157	0.166666667	0.129595694	1	0	1
hsa04930	Type II diabetes mellitus	C00022	0.980392157	0.166666667	0.129595694	0	1	-1
hsa00760	Nicotinate and nicotinamide metabolism	C00122;C00022;C00049	2.941176471	0.054545455	0.130074666	1	2	-0.333333333
hsa00061	Fatty acid biosynthesis	C06423;C01571;C00383	2.941176471	0.051724138	0.145897452	0	3	-1
hsa04720	Long-term potentiation	C00025	0.980392157	0.142857143	0.149515201	1	0	1
hsa05017	Spinocerebellar ataxia	C00025	0.980392157	0.142857143	0.149515201	1	0	1
hsa05030	Cocaine addiction	C00025	0.980392157	0.142857143	0.149515201	1	0	1
hsa05033	Nicotine addiction	C00025	0.980392157	0.142857143	0.149515201	1	0	1
hsa05200	Pathways in cancer	C00122;C00149	1.960784314	0.064516129	0.157170939	1	1	0
hsa05022	Pathways of neurodegeneration - multiple diseases	C00025;C00009	1.960784314	0.0625	0.165276204	2	0	1
hsa04724	Glutamatergic synapse	C00025	0.980392157	0.125	0.16898321	1	0	1
hsa05143	African trypanosomiasis	C00078	0.980392157	0.125	0.16898321	1	0	1
hsa04727	GABAergic synapse	C00025	0.980392157	0.111111111	0.188009857	1	0	1
hsa04730	Long-term depression	C00025	0.980392157	0.111111111	0.188009857	1	0	1
hsa04713	Circadian entrainment	C00025	0.980392157	0.111111111	0.188009857	1	0	1
hsa05031	Amphetamine addiction	C00025	0.980392157	0.111111111	0.188009857	1	0	1
hsa00600	Sphingolipid metabolism	C00065;C00346	1.960784314	0.057142857	0.189998325	2	0	1
hsa00230	Purine metabolism	C00294;C00366;C00209;C01444	3.921568627	0.03960396	0.198999621	1	3	-0.5
hsa04979	Cholesterol metabolism	C05465	0.980392157	0.1	0.206605051	0	1	-1
hsa05034	Alcoholism	C00025	0.980392157	0.1	0.206605051	1	0	1
hsa05415	Diabetic cardiomyopathy	C00022;C00546	1.960784314	0.051282051	0.223649957	0	2	-1
hsa04122	Sulfur relay system	C00979	0.980392157	0.090909091	0.224778481	0	1	-1
hsa04072	Phospholipase D signaling pathway	C00025	0.980392157	0.090909091	0.224778481	1	0	1

hsa04540	Gap junction	C00025	0.980392157	0.090909091	0.224778481	1	0	1
hsa04928	Parathyroid hormone synthesis, secretion and action	C00009	0.980392157	0.090909091	0.224778481	1	0	1
hsa04911	Insulin secretion	C00022	0.980392157	0.083333333	0.242539621	0	1	-1
hsa04721	Synaptic vesicle cycle	C00025	0.980392157	0.083333333	0.242539621	1	0	1
hsa00592	alpha-Linolenic acid metabolism	C06427;C00157	1.960784314	0.045454545	0.266274718	1	1	0
hsa05131	Shigellosis	C00407	0.980392157	0.071428571	0.276861876	0	1	-1
hsa05014	Amyotrophic lateral sclerosis	C00025	0.980392157	0.071428571	0.276861876	1	0	1
hsa00120	Primary bile acid biosynthesis	C00245;C05465	1.960784314	0.042553191	0.291900129	0	2	-1
hsa04066	HIF-1 signaling pathway	C00022	0.980392157	0.066666667	0.293440902	0	1	-1
hsa00380	Tryptophan metabolism	C00078;C01717;C05830	2.941176471	0.036144578	0.294782712	1	2	-0.333333333
hsa00982	Drug metabolism - cytochrome P450	C07185;C16650;C05011	2.941176471	0.034482759	0.320019218	1	2	-0.333333333
hsa04931	Insulin resistance	C00022	0.980392157	0.052631579	0.356076152	0	1	-1
hsa00310	Lysine degradation	C00047;C12455	1.960784314	0.035714286	0.367751976	1	1	0
hsa00910	Nitrogen metabolism	C00025	0.980392157	0.05	0.370855682	1	0	1
hsa00053	Ascorbate and aldarate metabolism	C00022;C01114	1.960784314	0.035087719	0.376013744	0	2	-1
hsa01232	Nucleotide metabolism	C00294;C00015	1.960784314	0.034482759	0.384232183	1	1	0
hsa04148	Efferocytosis	C04230	0.980392157	0.047619048	0.385299238	1	0	1
hsa04152	AMPK signaling pathway	C00022	0.980392157	0.045454545	0.399414384	0	1	-1
hsa00740	Riboflavin metabolism	C01727	0.980392157	0.041666667	0.426688859	0	1	-1
hsa00565	Ether lipid metabolism	C04317	0.980392157	0.04	0.439862488	1	0	1
hsa04024	cAMP signaling pathway	C20793	0.980392157	0.04	0.439862488	0	1	-1
hsa05012	Parkinson disease	C00009	0.980392157	0.038461538	0.452736311	1	0	1
hsa04973	Carbohydrate digestion and absorption	C00246	0.980392157	0.037037037	0.465317085	0	1	-1
hsa00750	Vitamin B6 metabolism	C00314	0.980392157	0.034482759	0.489625766	0	1	-1
hsa00780	Biotin metabolism	C00047	0.980392157	0.034482759	0.489625766	1	0	1

hsa04216	Ferroptosis	C00025	0.980392157	0.034482759	0.489625766	1	0	1
hsa01040	Biosynthesis of unsaturated fatty acids	C06427;C06426	1.960784314	0.027027027	0.508283984	0	2	-1
hsa00010	Glycolysis / Gluconeogenesis	C00022	0.980392157	0.032258065	0.512839628	0	1	-1
hsa00730	Thiamine metabolism	C00022	0.980392157	0.032258065	0.512839628	0	1	-1
hsa00400	Phenylalanine, tyrosine and tryptophan biosynthesis	C00078	0.980392157	0.028571429	0.556176111	1	0	1
hsa00480	Glutathione metabolism	C00025	0.980392157	0.026315789	0.586152398	1	0	1
hsa04977	Vitamin digestion and absorption	C00864	0.980392157	0.023809524	0.623023409	0	1	-1
hsa04726	Serotonergic synapse	C00078	0.980392157	0.023809524	0.623023409	1	0	1
hsa00900	Terpenoid backbone biosynthesis	C00022	0.980392157	0.02173913	0.656638805	0	1	-1
hsa00140	Steroid hormone biosynthesis	C04555;C05476	1.960784314	0.01980198	0.677932343	0	2	-1
hsa00071	Fatty acid degradation	C02990	0.980392157	0.02	0.687283452	1	0	1
hsa00440	Phosphonate and phosphinate metabolism	C00022	0.980392157	0.017857143	0.728243163	0	1	-1
hsa00040	Pentose and glucuronate interconversions	C00022	0.980392157	0.016949153	0.74668332	0	1	-1
hsa01212	Fatty acid metabolism	C00383;C02990	1.960784314	0.016393443	0.774657761	1	1	0
hsa00590	Arachidonic acid metabolism	C00157	0.980392157	0.012658228	0.84162737	1	0	1
hsa00524	Neomycin, kanamycin and gentamicin biosynthesis	C00025	0.980392157	0.012345679	0.848911989	1	0	1
hsa00860	Porphyrin metabolism	C00025;C00486	1.960784314	0.013513514	0.858835683	2	0	1
hsa00520	Amino sugar and nucleotide sugar metabolism	C00270	0.980392157	0.008474576	0.937021879	0	1	-1
hsa01250	Biosynthesis of nucleotide sugars	C00270	0.980392157	0.005	0.991186791	0	1	-1

DA score, Differential Abundance Score

Table S3. Differential fecal metabolites before and after a 12-week BBr60 intervention

Name	MS2 score	mz	type	Formula	HMDB	CAS	KEGG ID	MEAN BBr60-before	MEAN BBr60-after	VIP	P-VALUE	FOLD CHANGE
Cholic acid	4.0000	391.2844	POS	C24H40O5	HMDB0000619	81-25-4	C00695	0.0532	0.0237	1.6886	0.0446	0.4463

Maltose	3.9900	365.1054	POS	C12H22O11	HMDB0000163	69-79-4	C00208	0.3513	0.2193	1.2474	0.0225	0.6243
N-Acetylgalactosamine	3.9900	220.0827	NEG	C8H15NO6	HMDB0006480	14131-60-3	C05021	1.7753	0.9365	2.1698	0.0030	0.5275
Val-Val	3.9900	215.1401	NEG	C10H20N2O3	HMDB0029140	3918-94-3		8.3660	4.8582	2.2877	0.0415	0.5807
Ile-Leu	3.9900	245.1860	POS	C12H24N2O3	HMDB0028911	26462-22-6		6.2610	4.3123	2.0255	0.0332	0.6888
6-(Dimethylamino)purine	3.9900	164.0931	POS	C7H9N5	HMDB0000473	938-55-6		0.0618	0.0314	1.8235	0.0479	0.5091
12-Hydroxystearic acid	3.9900	299.2592	NEG	C18H36O3	HMDB0061706	106-14-9		74.4455	85.2251	1.3585	0.0274	1.1448
Methionine sulfoxide	3.9800	166.0532	POS	C5H11NO3S	HMDB0002005	62697-73-8	C02989	0.1009	0.0669	1.9816	0.0002	0.6635
Methionine	3.9800	150.0583	POS	C5H11NO2S	HMDB0033951	59-51-8	C01733	0.5877	0.4092	2.5163	0.0100	0.6962
Isoleucine	3.9800	130.0874	NEG	C6H13NO2	HMDB0000172	73-32-5	C00407	17.5515	9.3707	2.5394	0.0162	0.5339
Phenylalanine	3.9800	166.0862	POS	C9H11NO2	HMDB0000159	63-91-2	C00079	2.0962	1.2527	2.4078	0.0345	0.5976
Glycohyodeoxycholic acid	3.9800	448.3071	NEG	C26H43NO5	HMDB0255669	13042-33-6		8.3912	2.1521	1.9702	0.0067	0.2565
Thymine	3.9800	125.0357	NEG	C5H6N2O2	HMDB0000262	65-71-4	C00178	11.6132	5.7528	2.2004	0.0070	0.4954
Serine	3.9800	106.0498	POS	C3H7NO3	HMDB0000187	56-45-1	C00065	0.0603	0.0503	1.9588	0.0307	0.8349
N-Acetylglucosamine	3.9700	220.0827	NEG	C8H15NO6	HMDB0062641	7512-17-6	C00140	1.7753	0.9365	2.1698	0.0030	0.5275
3-Amino-4-methylpentanoic acid	3.9700	130.0874	NEG	C6H13NO2	HMDB0245808	5699-54-7		17.5515	9.3707	2.5394	0.0162	0.5339
Arg-Phe	3.9700	322.1871	POS	C15H23N5O3	HMDB0028716	2047-13-4		0.0540	0.0376	2.1496	0.0136	0.6962
Taurine	3.9700	124.0075	NEG	C2H7NO3S	HMDB0000251	107-35-7	C00245	21.6038	9.8224	1.6736	0.0063	0.4547
Tryptophan	3.9700	205.0971	POS	C11H12N2O2	HMDB0030396	73-22-3	C00078	0.3690	0.1462	2.1467	0.0446	0.3963
1-Methyl-6-oxo-1,6-dihydropyridine-3-carboxylic acid	3.9700	152.0355	NEG	C7H7NO3		3719-45-7		0.1232	0.1908	1.1561	0.0496	1.5492
Deoxyguanosine	3.9700	268.1042	POS	C10H13N5O4	HMDB0000085	961-07-9	C00330	0.0312	0.0200	1.8353	0.0446	0.6413
4-Ethoxybenzoic acid	3.9700	147.0453	NEG	C9H10O3		619-86-3		0.7528	0.3663	2.7377	0.0010	0.4866
13(S)-HODE	3.9700	295.2278	NEG	C18H32O3	HMDB0004667	29623-28-7	C14762	5.4585	2.9070	2.4281	0.0017	0.5326
Linoleic acid	3.9700	279.2330	NEG	C18H32O2	HMDB0000673	60-33-3	C01595	340.7685	153.5564	2.5076	0.0004	0.4506
Docosapentaenoic acid (DPA)	3.9700	329.2486	NEG	C22H34O2	HMDB0006528	2234-74-4	C16513	3.3747	1.5833	1.6198	0.0430	0.4692
Trehalose	3.9600	365.1054	POS	C12H22O11	HMDB0000975	99-20-7	C01083	0.3513	0.2193	1.2474	0.0225	0.6243
Leu-Ile	3.9600	245.1860	POS	C12H24N2O3	HMDB0028932	36077-41-5		6.2610	4.3123	2.0255	0.0332	0.6888

9-Ethyladenine	3.9600	164.0931	POS	C7H9N5		2715-68-6		0.0618	0.0314	1.8235	0.0479	0.5091
2-Hydroxystearic acid	3.9600	299.2592	NEG	C18H36O3	HMDB0062549	26531-80-6	C03045	74.4455	85.2251	1.3585	0.0274	1.1448
Glu-Arg	3.9600	304.1617	POS	C11H21N5O5	HMDB0028813	7219-59-2		0.0470	0.0283	1.7187	0.0345	0.6013
Threonic acid	3.9600	135.0300	NEG	C4H8O5	HMDB0062620	7306-96-9	C01620	0.4363	0.4113	1.4378	0.0332	0.9425
Glycocholic acid	3.9600	466.3164	POS	C26H43NO6	HMDB0000138	475-31-0	C01921	0.1538	0.0289	1.8981	0.0332	0.1879
Glycoursodeoxycholic acid	3.9600	450.3215	POS	C26H43NO5	HMDB0000708	64480-66-6		0.2798	0.0737	1.7713	0.0216	0.2635
Uracil	3.9600	111.0201	NEG	C4H4N2O2	HMDB0000300	66-22-8	C00106	48.7970	28.9972	2.8264	0.0029	0.5942
Aminopicoline	3.9600	109.0760	POS	C6H8N2	HMDB0246517	695-34-1		0.0438	0.0650	1.3147	0.0430	1.4852
3-Amino-4-hydroxybenzoic acid	3.9500	154.0499	POS	C7H7NO3	HMDB0304941	1571-72-8	C12115	0.0543	0.0775	1.3245	0.0105	1.4276
2-Keto-3-deoxygalactonic acid	3.9500	177.0406	NEG	C6H10O6	HMDB0001353	17510-99-5	C01216	5.0672	1.6048	2.7506	0.0005	0.3167
Pro-Phe	3.9500	263.1391	POS	C14H18N2O3	HMDB0011179	13589-02-1		0.1902	0.1178	2.6399	0.0061	0.6192
Phe-Val	3.9500	265.1548	POS	C14H20N2O3	HMDB0029008	3918-90-9		0.8931	0.5016	2.4264	0.0048	0.5616
Deoxyinosine	3.9500	251.0786	NEG	C10H12N4O4	HMDB0000071	890-38-0	C05512	36.8536	23.2784	2.3826	0.0234	0.6316
Guvacoline (hydrochloride)	3.9500	142.0863	POS	C7H11NO2		6197-39-3	C16821	0.0199	0.0327	1.6822	0.0100	1.6440
2-Ketobutyric acid	3.9500	101.0245	NEG	C4H6O3	HMDB0000005	600-18-0	C00109	2.8774	2.1203	1.2716	0.0004	0.7369
Sphingosine	3.9500	300.2897	POS	C18H37NO2	HMDB0000252	123-78-4	C00319	16.6441	7.3454	2.0589	0.0274	0.4413
NAE(18:2)	3.9500	324.2898	POS	C20H37NO2	HMDB0012252	68171-52-8		3.6243	0.8938	1.7824	0.0110	0.2466
NAE(18:3(6Z,9Z,12Z))	3.9500	322.2742	POS	C20H35NO2	HMDB0013624	150314-37-7		0.2720	0.1074	1.8234	0.0162	0.3947
Leucine	3.9400	130.0874	NEG	C6H13NO2	HMDB0062203	61-90-5	C00123	17.5515	9.3707	2.5394	0.0162	0.5339
2,5-Dimethylpyrazine	3.9400	109.0760	POS	C6H8N2	HMDB0035289	123-32-0		0.0438	0.0650	1.3147	0.0430	1.4852
2-Ethylpyrazine	3.9400	109.0760	POS	C6H8N2	HMDB0031849	13925-00-3		0.0438	0.0650	1.3147	0.0430	1.4852
2,6-Dimethylpyrazine	3.9400	109.0760	POS	C6H8N2	HMDB0035248	108-50-9		0.0438	0.0650	1.3147	0.0430	1.4852
NAE(18:3(9Z,12Z,15Z))	3.9400	322.2742	POS	C20H35NO2	HMDB0013624	57086-93-8		0.2720	0.1074	1.8234	0.0162	0.3947
Glycylleucine	3.9400	187.1088	NEG	C8H16N2O3	HMDB0000759	869-19-2	C02155	8.1926	4.7497	2.8699	0.0073	0.5798
Phe-Pro	3.9400	261.1246	NEG	C14H18N2O3	HMDB0011177	7669-65-0		0.5159	0.3060	2.7009	0.0115	0.5930
Sucralose (Xenobiotic)	3.9400	395.0075	NEG	C12H19Cl3O8	HMDB0031554	56038-13-2	C12285	7.1305	24.1409	1.9757	0.0005	3.3856

2-Methyltetrahydrofuran-3-one	3.9400	99.0452	NEG	C5H8O2	HMDB0031178	3188-00-9		1.0987	0.4557	2.4508	0.0003	0.4147
2,3-Pentanedione	3.9400	99.0452	NEG	C5H8O2	HMDB0031598	600-14-6		1.0987	0.4557	2.4508	0.0003	0.4147
3-Methyl-2-oxovaleric acid	3.9400	129.0558	NEG	C6H10O3	HMDB0000491	1460-34-0	C03465	36.2015	14.7338	2.9023	0.0000	0.4070
Hydroxytyrosol	3.9400	153.0558	NEG	C8H10O3	HMDB0005784	10597-60-1		0.4296	0.1265	1.7651	0.0191	0.2946
NAE(16:0)	3.9400	300.2899	POS	C18H37NO2	HMDB0002100	544-31-0	C16512	3.3480	1.5507	2.2726	0.0023	0.4632
3-Methylbut-2-enoic acid	3.9300	99.0452	NEG	C5H8O2	HMDB0000509	541-47-9		1.0987	0.4557	2.4508	0.0003	0.4147
Ketoleucine	3.9300	129.0558	NEG	C6H10O3	HMDB0000695	816-66-0	C00233	36.2015	14.7338	2.9023	0.0000	0.4070
2-Ketocaproic acid	3.9300	129.0558	NEG	C6H10O3	HMDB0001864	2492-75-3	C00902	36.2015	14.7338	2.9023	0.0000	0.4070
N-Methyl-L-asparagine	3.9300	147.0765	POS	C5H10N2O3		7175-34-0		0.1892	0.1006	2.0197	0.0052	0.5315
Gly-Phe	3.9300	221.0930	NEG	C11H14N2O3	HMDB0028848	721-66-4		0.3688	0.2333	2.3410	0.0307	0.6325
N-Acetylmannosamine	3.9300	222.0973	POS	C8H15NO6	HMDB0001129	3615-17-6	C00645	0.1091	0.0722	1.8594	0.0055	0.6615
2-Ethyl-2-hydroxybutyric acid	3.9300	131.0715	NEG	C6H12O3	HMDB0001975	3639-21-2		61.6185	18.0582	2.5376	0.0003	0.2931
2-Hydroxyhexanoic acid	3.9300	131.0715	NEG	C6H12O3	HMDB0001624	6064-63-7		61.6185	18.0582	2.5376	0.0003	0.2931
Thymidine	3.9300	241.0830	NEG	C10H14N2O5	HMDB0000273	50-89-5	C00214	11.3832	5.9458	2.6149	0.0039	0.5223
Arabinono-1,4-lactone	3.9300	147.0300	NEG	C5H8O5	HMDB0001900	51532-86-6	C01114	1.0633	0.5686	1.0296	0.0319	0.5347
alpha-Ketoisovaleric acid	3.9300	115.0401	NEG	C5H8O3	HMDB0000019	759-05-7	C00141	17.5087	6.9740	2.8580	0.0000	0.3983
Norleucine	3.9200	130.0874	NEG	C6H13NO2	HMDB0251526	104809-14-5	C01933	17.5515	9.3707	2.5394	0.0162	0.5339
Glutamine	3.9200	145.0619	NEG	C5H10N2O3	HMDB0000641	56-85-9	C00064	0.5875	0.3009	2.5039	0.0034	0.5122
2-Hydroxy-3-methylbutyric acid	3.9200	117.0558	NEG	C5H10O3	HMDB0000407	4026-18-0		48.1401	13.5910	2.1321	0.0016	0.2823
5-Aminosalicylic Acid	3.9100	152.0355	NEG	C7H7NO3	HMDB0014389	89-57-6	D00377|C07138	0.1232	0.1908	1.1561	0.0496	1.5492
Dethiobiotin	3.9100	213.1245	NEG	C10H18N2O3	HMDB0003581	533-48-2	C01909	0.6775	0.4134	2.6269	0.0169	0.6101
Nicotinamide riboside (NR)	3.9000	255.0976	POS	C11H15N2O5	HMDB0000855	1341-23-7	C03150	0.0849	0.1295	1.3265	0.0234	1.5247
Glyco-gamma-muricholic acid	3.9000	464.3020	NEG	C26H43NO6	HMDB0240607	32747-08-3		4.9789	0.7902	2.0783	0.0039	0.1587
Acamprosate (calcium)	3.9000	180.0339	NEG	C5H11NO4S	HMDB0014797	77337-76-9	D07058	0.7973	0.4069	1.4703	0.0199	0.5104
Indoleacetic acid	3.9000	174.0561	NEG	C10H9NO2	HMDB0000197	87-51-4	C00954	0.9157	1.7802	1.3231	0.0136	1.9442
gamma-Glutamylphenylalanine	3.9000	293.1145	NEG	C14H18N2O5	HMDB0000594	7432-24-8		0.2801	0.1621	2.3846	0.0253	0.5788

Hydroxyisocaproic acid	3.8900	131.0715	NEG	C6H12O3	HMDB0000665	498-36-2	C03264	61.6185	18.0582	2.5376	0.0003	0.2931
Indolelactic acid	3.8900	204.0666	NEG	C11H11NO3	HMDB0000671	1821-52-9	C02043	4.9035	3.8014	1.4725	0.0479	0.7752
2'-Deoxyuridine	3.8900	227.0674	NEG	C9H12N2O5	HMDB0000012	951-78-0	C00526	6.7566	4.3303	2.3856	0.0216	0.6409
Melibiose	3.8800	365.1054	POS	C12H22O11	HMDB0000048	60033-03-6	C05402	0.3513	0.2193	1.2474	0.0225	0.6243
Homocarnosine	3.8800	239.1152	NEG	C10H16N4O3	HMDB0000745	3650-73-5	C00884	0.4069	1.3205	1.3847	0.0430	3.2455
Formylmethionine	3.8800	176.0389	NEG	C6H11NO3S	HMDB0001015	4289-98-9	C03145	2.0316	1.0372	2.1440	0.0076	0.5106
Hydroxyphenyllactic acid	3.8800	181.0508	NEG	C9H10O4	HMDB0000755	306-23-0	C03672	10.7216	6.2335	2.6402	0.0006	0.5814
Catechin	3.8800	289.0719	NEG	C15H14O6	HMDB0002780	154-23-4	C06562	2.2607	1.2419	1.9801	0.0008	0.5493
2-Hydroxy-2-methylbutyric acid	3.8700	117.0558	NEG	C5H10O3	HMDB0001987	3739-30-8		48.1401	13.5910	2.1321	0.0016	0.2823
Glyco-beta-muricholic acid	3.8600	464.3020	NEG	C26H43NO6	HMDB0240607	66225-78-3		4.9789	0.7902	2.0783	0.0039	0.1587
Taurohyodeoxycholic acid	3.8600	498.2900	NEG	C26H45NO6S	HMDB0247202	386523		29.3999	45.5254	1.6126	0.0225	1.5485
Nipecotic acid	3.8500	130.0863	POS	C6H11NO2	HMDB0255618	498-95-3		0.0354	0.0595	1.4880	0.0162	1.6797
Car(18:1)	3.8500	426.3582	POS	C25H47NO4	HMDB0013337	13962-05-5		0.5189	0.2433	1.6449	0.0253	0.4690
MES	3.8500	194.0496	NEG	C6H13NO4S	HMDB0246678	4432-31-9		0.5644	1.5587	1.2647	0.0105	2.7615
Ferulate	3.8500	193.0506	NEG	C10H10O4	HMDB0000954	537-98-4	C01494	6.2274	3.1033	1.9908	0.0045	0.4983
Prunasin	3.8500	296.1131	POS	C14H17NO6	HMDB0034934	99-18-3	C00844	0.0529	0.1598	1.3629	0.0234	3.0227
3-Methyladenine	3.8400	150.0761	POS	C6H7N5	HMDB0011600	5142-23-4	C00913	0.0698	0.0488	2.0574	0.0400	0.6989
4-Hydroxyphenylpyruvic acid	3.8400	179.0350	NEG	C9H8O4	HMDB0000707	156-39-8	C01179	4.9774	2.4819	2.1105	0.0009	0.4986
Glycodeoxycholic acid	3.8300	450.3215	POS	C26H43NO5	HMDB0000631	360-65-6|16409-34-0	C05464	0.2798	0.0737	1.7713	0.0216	0.2635
2-Methylhippuric acid	3.8200	192.0668	NEG	C10H11NO3	HMDB0011723	27115-50-0		0.3295	1.1630	1.9030	0.0014	3.5300
Tosufloxacin (tosylate hydrate)	3.8200	405.1185	POS	C19H15F3N4O3		1400591-39-0	D02317	0.2490	0.0632	1.8037	0.0063	0.2537
Glycochenodeoxycholic acid	3.8100	450.3215	POS	C26H43NO5	HMDB0014642	13311-84-7	C07653	0.2798	0.0737	1.7713	0.0216	0.2635
3-Cresol	3.8000	107.0503	NEG	C7H8O	HMDB0002048	108-39-4	C01467	0.1910	0.0637	2.3918	0.0000	0.3333
2-Methylphenol	3.8000	107.0503	NEG	C7H8O	HMDB0002055	95-48-7	C01542	0.1910	0.0637	2.3918	0.0000	0.3333
Carbidopa	3.8000	209.0920	POS	C10H14N2O4	HMDB0014336	28860-95-9		0.0171	0.0222	1.9026	0.0319	1.2955
Ethyl 3-hydroxybenzoate	3.7900	165.0558	NEG	C9H10O3		7781-98-8		14.7074	6.4068	2.9806	0.0001	0.4356

2-Phenyllactic acid	3.7900	165.0558	NEG	C9H10O3	HMDB0142137	515-30-0		14.7074	6.4068	2.9806	0.0001	0.4356
Ethyl 4-hydroxybenzoate	3.7800	165.0558	NEG	C9H10O3	HMDB0032573	120-47-8	D01647	14.7074	6.4068	2.9806	0.0001	0.4356
Butyllactate	3.7800	145.0871	NEG	C7H14O3	HMDB0040254	138-22-7		0.4504	0.2115	2.2097	0.0006	0.4695
5-Oxooctanoic acid	3.7700	157.0871	NEG	C8H14O3		3637-14-7		0.6333	0.4467	1.8862	0.0274	0.7054
11-Dehydrocorticosterone	3.7700	325.1843	NEG	C21H28O4	HMDB0004029	72-23-1	C05490	21.2603	33.1523	1.3550	0.0006	1.5594
Taurodeoxycholic acid	3.7600	498.2900	NEG	C26H45NO6S	HMDB0000896	516-50-7	C05463	29.3999	45.5254	1.6126	0.0225	1.5485
Docebenone	3.7600	325.1843	NEG	C21H26O3	HMDB0247732	80809-81-0	C01349	21.2603	33.1523	1.3550	0.0006	1.5594
Monoethylglycinexylidide (MEGX)	3.7500	189.1386	POS	C12H18N2O	HMDB0060656	7728-40-7	C16561	0.0083	0.0273	1.6183	0.0234	3.2834
PC(16:0/20:4)	3.7400	782.5689	POS	C44H80NO8P	HMDB0007982	35418-58-7		0.9094	0.4111	1.2486	0.0263	0.4520
Isoferulic acid	3.7300	193.0506	NEG	C10H10O4	HMDB0000955	25522-33-2	C10470	6.2274	3.1033	1.9908	0.0045	0.4983
Desaminotyrosine	3.7300	165.0558	NEG	C9H10O3	HMDB0002199	501-97-3	C01744	14.7074	6.4068	2.9806	0.0001	0.4356
Pyruvaldehyde	3.7300	71.0139	NEG	C3H4O2	HMDB0001167	78-98-8	C00546	3.6726	1.8524	1.2163	0.0307	0.5044
Palmitoylcarnitine (Car(16:0))	3.7300	400.3424	POS	C23H45NO4	HMDB0000222	2364-67-2	C02990	0.8759	0.3913	1.4405	0.0296	0.4468
Phenylephrine	3.7300	168.1019	POS	C9H13NO2	HMDB0002182	59-42-7	C07441	0.0310	0.0886	1.5951	0.0041	2.8565
2,2,6,6-Tetramethyl-4-piperidinyl 2-methylacrylate	3.7300	226.1802	POS	C13H23NO2		31582-45-3		0.0433	0.0657	2.6390	0.0001	1.5167
3,5-Dimethoxycinnamic acid	3.7300	207.0662	NEG	C11H12O4		16909-11-8		10.0683	5.2897	1.1988	0.0063	0.5254
(1-Hydroxycyclohexyl)acetic acid	3.7200	157.0871	NEG	C8H14O3		14399-63-4		0.6333	0.4467	1.8862	0.0274	0.7054
Homovanillic acid	3.7200	181.0508	NEG	C9H10O4	HMDB0000118	306-08-1	C05582	7.5206	3.5563	1.3999	0.0176	0.4729
Triamterene	3.6600	254.1136	POS	C12H11N7	HMDB0001940	396-01-0	D00386	0.0565	0.0959	1.7931	0.0048	1.6981
Tazarotene	3.6200	352.1393	POS	C21H21NO2S	HMDB0014937	118292-40-3	C12531	0.0754	0.1663	1.7723	0.0162	2.2047
5-Hydroxytryptophan	3.6100	219.0807	NEG	C11H12N2O3	HMDB0000472	4350-09-8	C00643	0.2788	0.1452	1.8944	0.0345	0.5206
4-Hydroxyderricin	3.4900	339.1551	POS	C21H22O4		55912-03-3		0.0498	0.1382	1.7229	0.0073	2.7765
4-(N-Methylacetamido)benzoic acid	2.7500	194.0812	POS	C10H11NO3				0.0415	0.0289	1.0130	0.0285	0.6957
2,8-Diazaspiro[5.5]undecane-1,7-dione	2.7500	183.1128	POS	C9H14N2O2				0.0504	0.0693	1.7691	0.0088	1.3749
4-(Dimethylamino)phenylalanine	2.7500	209.1285	POS	C11H16N2O2				0.0229	0.0371	1.4664	0.0041	1.6168
Ile-Asn	2.7500	246.1448	POS	C10H19N3O4				0.0809	0.0601	2.1510	0.0296	0.7427

Pro-Ile	2.7500	227.1401	NEG	C11H20N2O3	HMDB0304810			1.2341	0.7840	2.5978	0.0155	0.6353
Ile-Pro	2.7500	229.1547	POS	C11H20N2O3	HMDB0011174	37462-92-3		0.3804	0.2453	2.5852	0.0039	0.6451
8-Methylquinolin-2(1H)-one	2.7500	160.0757	POS	C10H9NO				0.0104	0.0315	1.1511	0.0274	3.0181
Mepirizole	2.7500	235.1190	POS	C11H14N4O2	HMDB0254454			0.1675	0.2335	1.3045	0.0345	1.3935
2,3-Dihydroxy-3-(2-hydroxypropan-2-yl)-8a-methyl-1,2,3a,4,5,8-hexahydroazulene-6-carboxylic acid	2.7500	283.1552	NEG	C15H24O5				0.9969	2.0705	1.0593	0.0149	2.0770
7-Nitro-2,3,4,5-tetrahydro-1H-1-benzazepin-2-one	2.7500	207.0764	POS	C10H10N2O3				0.0093	0.0152	1.7022	0.0076	1.6345
Brucine	2.7500	395.1965	POS	C23H26N2O4	HMDB0249406			0.0073	0.0574	1.8947	0.0021	7.8724
L-Tyrosine, O-(1,1-dimethylethyl)-	2.7500	236.1292	NEG	C13H19NO3				0.2382	1.2366	1.4444	0.0125	5.1904
1-Aminocyclopentanecarboxylic acid ethyl ester	2.7500	158.1176	POS	C8H15NO2				0.0584	0.1759	1.4845	0.0385	3.0119
Harmane	2.7500	183.0917	POS	C12H10N2	HMDB0035196	486-84-0	C09209	0.1785	0.3244	1.5069	0.0371	1.8174
PC(36:5)	2.7500	780.5539	POS	C44H78NO8P	HMDB0007890			0.0208	0.0101	1.6652	0.0018	0.4842
L-Isoserine	2.7400	104.0354	NEG	C3H7NO3				0.6980	0.5414	2.3434	0.0253	0.7756
L-trans-5-Hydroxy-2-piperidinecarboxylic_acid	2.7400	146.0812	POS	C6H11NO3	HMDB0029426	50439-45-7		0.5045	0.2166	1.5395	0.0285	0.4293
Ser-Val	2.7400	203.1037	NEG	C8H16N2O4				2.7145	1.6395	2.4456	0.0319	0.6040
Tyr-Pro	2.7400	279.1340	POS	C14H18N2O4				0.0425	0.0269	2.0777	0.0092	0.6342
Val-Pro	2.7400	215.1390	POS	C10H18N2O3	HMDB0029135	20488-27-1		0.0928	0.0691	2.4276	0.0169	0.7443
1-Isopropyl-1H-benzimidazole-5-carboxylic acid	2.7400	203.0826	NEG	C11H12N2O2				3.9958	1.4497	2.3744	0.0479	0.3628
2-Hydroxy-6-methylquinoline-3-carbaldehyde	2.7400	188.0706	POS	C11H9NO2				0.5271	0.2056	2.1942	0.0371	0.3900
1-(4-Hydroxyphenyl)-2-methylaminoethanone	2.7400	164.0718	NEG	C9H11NO2				14.6351	7.4369	2.5772	0.0285	0.5082
4-Methylcatechol	2.7400	123.0452	NEG	C7H8O2	HMDB0000873	452-86-8	C06730	0.7496	0.2180	1.7237	0.0061	0.2909
Thr-Val-Leu	2.7400	332.2182	POS	C15H29N3O5				0.1751	0.1253	2.3526	0.0162	0.7154
4,5-Dimethyl-1,3-benzenediol	2.7400	137.0609	NEG	C8H10O2				0.5562	0.2816	1.4828	0.0052	0.5062
Ile-Gly-Ile	2.7400	300.1929	NEG	C14H27N3O4				1.3097	0.8012	2.3160	0.0234	0.6118
Isocytosine	2.7400	112.0505	POS	C4H5N3O				0.3413	0.2464	2.0586	0.0446	0.7220
2-Hydroxybenzaldehyde [(E)-(2-hydroxyphenyl)methylidene]hydrazone	2.7400	241.0971	POS	C14H12N2O2				0.0949	0.1251	1.5565	0.0285	1.3187

8-Oxoadenine	2.7400	152.0568	POS	C5H5N5O	HMDB0000542	21149-26-8		0.1917	0.1198	1.3258	0.0319	0.6248
1,1-Dimethyl-4-phenylpiperazin-1-ium cation	2.7400	191.1542	POS	C12H19N2				0.0450	0.0772	1.1436	0.0285	1.7141
4-[(Propan-2-yl)carbamoyl]benzoic acid	2.7400	206.0824	NEG	C11H13NO3	HMDB0060608			0.3702	1.5087	1.2249	0.0149	4.0755
Normorphine	2.7400	272.1280	POS	C16H17NO3	HMDB0041959	466-97-7	C11785	0.0496	0.4833	2.4971	0.0000	9.7476
Propachlor ESA	2.7400	256.0648	NEG	C11H15NO4S				0.3909	0.8691	2.3890	0.0002	2.2236
Citbrasine	2.7400	332.1131	POS	C17H17NO6	HMDB0037797	86680-34-4		0.0252	0.1450	1.7853	0.0058	5.7548
5-Methylisoxazol-3-amine	2.7400	99.0552	POS	C4H6N2O				0.0922	0.1144	1.3136	0.0125	1.2404
Bufexamac	2.7400	222.1136	NEG	C12H17NO3	HMDB0341139			0.7992	4.3654	1.5564	0.0446	5.4622
Dodemorph	2.7400	282.2792	POS	C18H35NO				7.0992	3.1161	1.9856	0.0199	0.4389
4-Chloro-7H-pyrrolo[2,3-d]pyrimidine-5-carbaldehyde	2.7400	179.9971	NEG	C7H4ClN3O				1.4626	9.2710	1.4718	0.0037	6.3385
7-Hydroxychromanone	2.7400	163.0402	NEG	C9H8O3				3.7900	1.3715	2.8002	0.0000	0.3619
RICININE	2.7400	182.0924	POS	C8H8N2O2	HMDB0042006	524-40-3	C01526	0.1189	0.1493	1.3604	0.0430	1.2557
E-(gamma-Glutamyl)-lysine	2.7400	274.1409	NEG	C11H21N3O5	HMDB0003869	17105-15-6		0.8292	0.4464	2.4752	0.0169	0.5384
2-HYDROXY-4-(METHYLTHIO)BUTYRIC ACID	2.7400	149.0279	NEG	C5H10O3S	HMDB0037115	120-91-2		9.8602	2.9723	1.9867	0.0020	0.3014
butabarbital	2.7400	213.1234	POS	C10H16N2O3	HMDB0014382	125-40-6	C07827	0.0626	0.1317	2.0075	0.0063	2.1033
9-HPODE	2.7400	311.2229	NEG	C18H32O4	HMDB0062434	29774-12-7	C14827	2.9651	1.5264	1.9603	0.0332	0.5148
Metaproterenol	2.7400	212.1282	POS	C11H17NO3	HMDB0014954	586-06-1	C07144	0.0951	0.2538	2.5325	0.0001	2.6679
Gabapentin	2.7400	172.1333	POS	C9H17NO2	HMDB0005015	60142-96-3		0.1431	0.1983	2.2002	0.0058	1.3853
Octenoylcarnitine (Car(8:1))	2.7300	286.2013	POS	C15H27NO4	HMDB0240750			0.0161	0.0379	1.6527	0.0012	2.3517
Thr-Glu	2.7300	249.1082	POS	C9H16N2O6				0.0302	0.0239	2.1393	0.0296	0.7909
Arg-Leu	2.7300	288.2030	POS	C12H25N5O3				0.1707	0.0872	2.4418	0.0037	0.5111
His-Leu	2.7300	267.1462	NEG	C12H20N4O3	HMDB0028889	7763-65-7	C05010	0.5357	0.2927	2.2917	0.0307	0.5463
Ile-Gln	2.7300	258.1459	NEG	C11H21N3O4				2.9669	1.5678	2.7959	0.0067	0.5284
Leu-Gly	2.7300	189.1234	POS	C8H16N2O3	HMDB0028929			0.2912	0.1934	2.5504	0.0125	0.6641
5,5-Dimethyl-3-(4-nitrobenzyl)-2,4-imidazolidinedione	2.7300	262.0835	NEG	C12H13N3O4				0.0940	0.1725	1.6783	0.0030	1.8362
Threoninyl-Proline	2.7300	217.1183	POS	C9H16N2O4	HMDB0341432			0.0158	0.0323	1.6975	0.0100	2.0521

Val-Phe	2.7300	263.1402	NEG	C14H20N2O3				2.8442	1.8746	2.1357	0.0274	0.6591
3-(3-Hydroxyphenyl)propionic acid sulfate	2.7300	245.0127	NEG	C9H10O6S	HMDB0094710			14.9121	9.8512	1.5744	0.0176	0.6606
2,3-Dihydroxy-3-methylbutyric acid	2.7300	133.0507	NEG	C5H10O4	HMDB0341247		C04039	83.9494	38.1414	1.4784	0.0120	0.4543
(2E,4E,10Z)-N-(2-Methylpropyl)hexadeca-2,4,10-trienamide	2.7300	306.2789	POS	C20H35NO				0.0389	0.0197	1.2628	0.0142	0.5056
N-Methyl-N-(methylsulfonyl)glycine	2.7300	166.0181	NEG	C4H9NO4S				52.5633	18.4494	1.5634	0.0234	0.3510
Ethyl sulfate	2.7300	124.9915	NEG	C2H6O4S	HMDB0031233	540-82-9		125.2975	14.7518	1.6030	0.0035	0.1177
(12Z)-9,10,11-Trihydroxyoctadec-12-enoic acid	2.7300	329.2335	NEG	C18H34O5				12.8359	7.1772	1.7466	0.0131	0.5592
alpha-Keto-gamma-(methylthio)butyric_acid	2.7300	147.0122	NEG	C5H8O3S	HMDB0001553	583-92-6	C01180	1.8088	0.6446	2.7903	0.0000	0.3563
LPC(22:6)	2.7300	568.3403	POS	C30H50NO7P				0.0030	0.0017	1.5191	0.0243	0.5695
Linoleoylcarnitine (Car(18:2))	2.7300	424.3425	POS	C25H45NO4	HMDB0241541	36816-10-1		0.3264	0.1223	1.9284	0.0045	0.3746
Arg-Pro	2.7200	272.1719	POS	C11H21N5O3	HMDB0028717	2418-69-1		0.2577	0.2057	2.2618	0.0216	0.7982
Gly-Arg	2.7200	232.1406	POS	C8H17N5O3				0.1296	0.0888	2.1233	0.0199	0.6854
Glu-Val	2.7200	245.1143	NEG	C10H18N2O5	HMDB0028832	5879-06-1		2.4348	1.6725	2.3189	0.0371	0.6869
Salicylaldehyde	2.7200	123.0440	POS	C7H6O2	HMDB0034170	90-02-8	C06202	0.0196	0.0121	2.3682	0.0023	0.6162
Hemiphloin	2.7200	433.1141	NEG	C21H22O10				0.6201	0.1834	2.0295	0.0080	0.2957
Atropic acid	2.7200	147.0453	NEG	C9H8O2				0.6291	0.3177	2.5722	0.0307	0.5049
Leu-Gly-Leu	2.7200	300.1930	NEG	C14H27N3O4				1.4659	0.7618	2.8929	0.0037	0.5197
Echinocystic acid 3-glucoside	2.7200	633.4017	NEG	C36H58O9				0.4324	0.1311	2.3082	0.0253	0.3031
1-Hydroxy-N-(3-nitrophenyl)-2-naphthamide	2.7200	307.0728	NEG	C17H12N2O4				0.5676	0.6877	1.1003	0.0479	1.2115
L-Tyrosinamide	2.7200	181.0972	POS	C9H12N2O2				0.0352	0.0743	1.5820	0.0063	2.1115
Azelnidipine	2.7200	583.2550	POS	C33H34N4O6	HMDB0248792			0.0183	0.0628	2.4573	0.0000	3.4266
Aigialomycin D	2.7200	333.1348	NEG	C18H22O6				2.2068	2.5762	1.5765	0.0149	1.1674
5beta-Pregnane-3alpha,17alpha,20alpha-triol	2.7200	337.2738	POS	C21H36O3	HMDB0006070	27178-64-9		0.0524	0.0289	1.9177	0.0023	0.5513
9Z,11E,13E-Octadecatrienoic acid methyl ester	2.7200	293.2476	POS	C19H32O2				0.1987	0.0901	2.1847	0.0115	0.4537
Methocarbamol	2.7200	242.1023	POS	C11H15NO5	HMDB0014567	532-03-6		0.0485	0.0816	1.3662	0.0183	1.6807
Creatine, ethyl ester	2.7100	160.1082	POS	C6H13N3O2	HMDB0250523			0.0214	0.0427	1.5234	0.0076	1.9927

5-(4-Chlorobenzyl)-1,3,4-oxadiazol-2-amine	2.7100	210.0431	POS	C9H8ClN3O				0.0098	0.0141	1.4779	0.0183	1.4328
Asn-Ile	2.7100	244.1302	NEG	C10H19N3O4				2.0128	1.1019	2.6232	0.0183	0.5474
Homoarecoline	2.7100	170.1176	POS	C9H15NO2	HMDB0038321	28125-84-0		0.0163	0.0231	2.0332	0.0025	1.4164
7-Methyladenine	2.7100	150.0770	POS	C6H7N5	HMDB0011614	935-69-3	C02241	0.0730	0.0536	2.0941	0.0496	0.7350
Diethyl 2,4-dimethylpyrrole-3,5-dicarboxylate	2.7100	238.1086	NEG	C12H17NO4				0.5590	0.9560	1.5613	0.0055	1.7101
Tripropylene glycol	2.7100	193.1439	POS	C9H20O4				0.0143	0.0197	1.5727	0.0162	1.3799
4-Acetamidophenol sulfate	2.7100	230.0129	NEG	C8H9NO5S	HMDB0059911	10066-90-7		0.3247	0.1796	1.7851	0.0319	0.5532
Glabrone	2.7100	359.0909	POS	C20H16O5	HMDB0029533	60008-02-8		0.0444	0.0182	1.4470	0.0032	0.4092
Cholic acid/Muricholic acid, isoleucine-conjugated	2.7100	520.3647	NEG	C30H51NO6				0.4426	0.0790	2.2915	0.0385	0.1784
Methanone, [1-(6-fluorohexyl)-1H-indol-3-yl]-1-naphthalenyl-	2.7000	374.1922	POS	C25H24FNO				0.0076	0.0169	1.7567	0.0043	2.2285
Lys-Pro	2.7000	244.1657	POS	C11H21N3O3	HMDB0028959	52766-27-5		0.2922	0.1940	2.2101	0.0207	0.6637
Sulfoacetic acid	2.7000	138.9708	NEG	C2H4O5S	HMDB0258590		C14179	0.3559	0.5139	1.1259	0.0136	1.4440
PS(16:0/16:0)	2.7000	736.5126	POS	C38H74NO10P	HMDB0000614	40290-42-4		0.0364	0.0117	1.4622	0.0371	0.3208
3-(1-Methyl-4-morpholinopyrazolo[3,4-d]pyrimidin-6-yl)phenol	2.7000	312.1445	POS	C16H17N5O2				0.1672	0.0366	1.7130	0.0358	0.2192
N-Benzyl-D-serine	2.7000	194.0823	NEG	C10H13NO3				0.4069	2.3329	1.6861	0.0035	5.7340
3-(2,5-Dioxo-1-pyrrolidinyl)benzoic acid	2.7000	218.0458	NEG	C11H9NO4				1.4909	0.6988	2.5901	0.0001	0.4687
DL-Threonine methyl ester	2.7000	134.0812	POS	C5H11NO3	HMDB0341314			0.0781	0.0355	1.6941	0.0131	0.4547
Isoleucylvaline	2.7000	231.1703	POS	C11H22N2O3	HMDB0028920			6.2449	4.2051	2.1505	0.0285	0.6734
Pesticide3_Fenobucarb_C12H17NO2_Bassa	2.7000	208.1333	POS	C12H17NO2	HMDB0252205		C14425	0.0130	0.0676	2.7522	0.0002	5.2087
1-(beta-D-Ribofuranosyl)-1,4-dihydronicotinamide	2.7000	257.1133	POS	C11H16N2O5	HMDB0011648		C15497	0.1776	0.4668	1.9689	0.0025	2.6281
Oleoylcarnitine (Car(18:1))	2.7000	426.3583	POS	C25H47NO4	HMDB0094687	13962-05-5		0.0952	0.0387	1.4525	0.0243	0.4066
Lys-Ala	2.6900	218.1501	POS	C9H19N3O3	HMDB0028944	17043-71-9		0.2884	0.2111	2.0287	0.0216	0.7320
Lys-Thr	2.6900	248.1607	POS	C10H21N3O4	HMDB0028961	97791-84-9		0.0412	0.0264	2.2175	0.0120	0.6399
3-Dehydroshikimic acid	2.6900	171.0300	NEG	C7H8O5				0.3511	0.1411	2.3089	0.0027	0.4018
N5-(1-Iminoethyl)-L-ornithine	2.6900	174.1237	POS	C7H15N3O2				0.0988	0.0406	1.2988	0.0371	0.4109
5-O-Demethylnobiletin	2.6900	389.1235	POS	C20H20O8	HMDB0037571	2174-59-6		0.3344	0.0623	1.8675	0.0142	0.1863

5-[[17-(5,6-dihydroxy-6-methylheptan-2-yl)-3,12-dihydroxy-4,4,10,13,14 -pentamethyl-2,3,5,6,7,11,12,15,16,17 -decahydro-1H-cyclopenta[a]phenanthren-2-yl]oxy]-3-hydroxy-3-methyl-5-oxopentanoic acid	2.6900	635.4163	NEG	C36H60O9	HMDB0036438	126882-55-1		0.1343	0.0678	2.4967	0.0149	0.5048
DEOXYCORTICOSTERONE ACETATE	2.6900	395.2175	POS	C23H32O4	HMDB0251003			0.0150	0.0951	1.7848	0.0034	6.3273
Methanone, (4-ethyl-1-naphthalenyl)[1-(5-fluoropentyl)-1H-indol-3-yl]-	2.6800	388.2082	POS	C26H26FNO	HMDB0259633			0.0038	0.0062	1.6148	0.0012	1.6433
Methanone, [1-(3-fluoropentyl)-1H-indol-3-yl]-1-naphthalenyl-	2.6800	360.1769	POS	C24H22FNO				0.0197	0.0493	1.5885	0.0253	2.5044
Isoleucyl-Histidine	2.6800	269.1609	POS	C12H20N4O3	HMDB0028909			0.1709	0.1042	2.4385	0.0216	0.6096
2,6-Diamino-4-hydroxy-5-N-methylformamidopyrimidine	2.6800	184.0831	POS	C6H9N5O2	HMDB0011657	77440-13-2	C04744	0.0606	0.0316	1.7080	0.0216	0.5210
Valyl-Proline	2.6800	215.1390	POS	C10H18N2O3				0.0234	0.0345	1.2883	0.0332	1.4748
1H-Imidazole-4,5-dicarbohydrazide	2.6800	183.0642	NEG	C5H8N6O2				0.3319	0.4788	1.3337	0.0155	1.4426
2-Amino-6,7-diphenyl-4(3H)-pteridinone	2.6800	316.1181	POS	C18H13N5O				0.0396	0.2322	1.7785	0.0142	5.8680
N-Cyclopentyl-2-(1-piperazinyl)-4-quinazolinamine	2.6800	298.2014	POS	C17H23N5				0.0886	0.1545	1.0463	0.0191	1.7443
Piperlonguminine	2.6800	274.1439	POS	C16H19NO3	HMDB0030187	5950-12-9		0.5080	0.1798	1.3624	0.0307	0.3539
15-Keto-protaglandin_F2alpha	2.6800	375.2125	POS	C20H32O5	HMDB0004240	35850-13-6	C05960	0.0294	0.0443	1.6472	0.0021	1.5100
Zidovudine	2.6800	266.0895	NEG	C10H13N5O4	HMDB0014638	30516-87-1	C07210	0.6590	0.3621	2.3985	0.0063	0.5494
2-Amino-3,8-dimethylimidazo-[4,5-f]quinoxaline (MeIQx)	2.6800	214.1077	POS	C11H11N5	HMDB0029864	77500-04-0	C19255	0.0619	0.1083	1.7722	0.0358	1.7502
Carbendazim	2.6700	192.0767	POS	C9H9N3O2	HMDB0031769	10605-21-7	C10897	0.0144	0.0281	1.3189	0.0019	1.9521
Eglumegad	2.6700	184.0616	NEG	C8H11NO4				4.0709	6.0627	1.7363	0.0027	1.4893
Methyl 4-(9H-purin-6-ylamino)benzoate	2.6700	270.0973	POS	C13H11N5O2				0.0438	0.1455	1.8148	0.0073	3.3245
Gardenin	2.6700	419.1340	POS	C21H22O9				1.1853	0.1474	2.0284	0.0063	0.1244
7-Hydroxy-1,3-dimethyl-2,4-dioxo-1,2,3,4-tetrahydro-6-pteridinecarbaldehyde	2.6600	235.0460	NEG	C9H8N4O4				0.3268	0.1569	1.7564	0.0253	0.4801
Diisopropyl_sulfide	2.6600	119.0896	POS	C6H14S	HMDB0029579	625-80-9		0.1359	0.0990	2.1507	0.0234	0.7284
2-[(6-Ethoxy-4-methyl-2-quinazolinyl)amino]-5,6-dihydro-4-pyrimidinol	2.6600	300.1443	POS	C15H17N5O2				0.0198	0.0929	2.7982	0.0000	4.6874
2-[(4-Amino-6-anilino-1,3,5-triazin-2-yl)methoxy]benzaldehyde	2.6600	322.1288	POS	C17H15N5O2				0.0304	0.0540	1.7548	0.0183	1.7744
Arachidonic sulfonic acid	2.6600	339.1997	NEG	C19H32O3S				15.7767	23.4454	1.2765	0.0012	1.4861
Isoleucyl-Threonine	2.6600	233.1496	POS	C10H20N2O4	HMDB0028917			0.4209	0.2454	2.3095	0.0199	0.5829

Tenuazonic acid	2.6500	198.1114	POS	C10H15NO3				0.0124	0.0230	1.4657	0.0120	1.8601
Arg-Val	2.6500	274.1874	POS	C11H23N5O3	HMDB0028722	2896-20-0		0.1107	0.0833	1.9557	0.0063	0.7519
Dovitinib	2.6500	393.1811	POS	C21H21FN6O	HMDB0251597			0.0050	0.0215	2.2378	0.0004	4.2733
Difenoxuron	2.6500	287.1391	POS	C16H18N2O3				0.0940	0.1471	1.3110	0.0110	1.5656
2-(4-Nitrophenyl)butyric acid	2.6400	210.0762	POS	C10H11NO4				0.0155	0.0301	1.8365	0.0142	1.9486
3-Cyano-7-hydroxycoumarin	2.6400	188.0354	POS	C10H5NO3				0.0171	0.0089	2.3987	0.0017	0.5221
3-Fluoro-4-(4-thiomorpholinyl)aniline	2.6400	213.0869	POS	C10H13FN2S				0.0235	0.0404	1.8706	0.0076	1.7215
4-(2-Hydroxyethyl)piperazin-2-one	2.6400	145.0973	POS	C6H12N2O2				0.0193	0.0261	1.0155	0.0345	1.3526
.delta.-Octalactone	2.6400	143.1067	POS	C8H14O2	HMDB0038310			0.0251	0.0324	1.3386	0.0216	1.2908
1-O-Hexadecyl-2-O-(2E-butenoyl)-sn-glyceryl-3-phosphocholine	2.6300	550.3863	POS	C28H56NO7P				0.0336	0.0169	1.9703	0.0332	0.5030
Methanone, [1-(5-fluoro-4-hydroxypentyl)-1H-indol-3-yl](4-methyl-1-naphthalenyl)-	2.6200	390.1871	POS	C25H24FNO2				0.0124	0.0211	1.6144	0.0096	1.7020
Pergolide sulfone	2.6200	347.1814	POS	C19H26N2O2S				0.0427	0.0893	1.6922	0.0100	2.0930
Lysine conjugated deoxycholic acid putative	2.6200	521.3949	POS	C30H52N2O5	HMDB0242400			0.0199	0.0077	2.0801	0.0120	0.3889
Isoquinoline, 5-[[(2S)-hexahydro-2-methyl-1H-1,4-diazepin-1-yl]sulfonyl]-4-methyl-	2.6100	320.1454	POS	C16H21N3O2S				0.1287	0.2004	1.2899	0.0307	1.5566
3-(1-Hydroxymethyl-1-propenyl)pentanedioic_acid	2.6100	203.0914	POS	C9H14O5	HMDB0033092			0.0758	0.0406	1.2826	0.0243	0.5357
3-(2-Keto-1-methyl-3H-imidazo[4,5-b]pyridin-6-yl)benzamide	2.6100	267.0907	NEG	C14H12N4O2				4.1370	1.7809	2.2347	0.0332	0.4305
Dulxanthone_C	2.6000	425.1922	POS	C25H28O6	HMDB0031917	4178-45-4		0.0067	0.0112	2.0435	0.0029	1.6823
2-(2,3-Dimethylphenyl)-7-methyl-1,2,4-triazaspiro[4.5]decane-3-thione	2.6000	288.1565	NEG	C16H23N3S				1.9577	0.8754	2.8859	0.0058	0.4472
2-[(2R,3S,7R,8R,8aS)-2,3,4'-Trihydroxy-4,4,7,8a-tetramethyl-6'-oxospiro [2,3,4a,5,6,7-hexahydro-1H-naphthalene-8,2'-3,8-dihydrofuro[2,3-e]isoindole]-7'-yl]-3-methylpentanoic acid	2.6000	514.2847	NEG	C29H41NO7				4.0246	4.1599	1.6251	0.0234	1.0336
17-Acetoxygrindelic acid	2.6000	377.2367	NEG	C22H34O5				1.1016	0.8163	1.7365	0.0084	0.7411
17-Methylene-4-androsten-3-one	2.6000	285.2214	POS	C20H28O				0.0419	0.0666	1.1646	0.0142	1.5904
PGF1alpha	2.6000	379.2490	POS	C20H36O5	HMDB0002685	745-62-0	C06475	0.0635	0.1947	1.4552	0.0120	3.0639
2-Propenethioamide, 3-[3,5-bis(1,1-dimethylethyl)-4-hydroxyphenyl]-2-cyano-, (2E)-	2.5900	317.1710	POS	C18H24N2OS				0.0214	0.0470	1.7803	0.0080	2.1963
Lysyl-Glycine	2.5900	204.1343	POS	C8H17N3O3	HMDB0304784			0.0707	0.0507	2.4872	0.0063	0.7167

Promethazine sulfoxide	2.5900	301.1397	POS	C17H20N2OS	HMDB0247377			0.0448	0.0774	2.0785	0.0105	1.7254
Alprazolam	2.5900	309.0871	POS	C17H13ClN4	HMDB0014548	28981-97-7	C06817	0.0752	0.1146	1.5233	0.0274	1.5244
1-Methoxy-N-(4-methylbenzyl)-2-propanamine	2.5900	194.1540	POS	C12H19NO				0.0071	0.0334	1.9651	0.0004	4.7279
Angelicolide	2.5900	381.2025	POS	C24H28O4	HMDB0029317	90826-58-7		0.0194	0.1012	1.8634	0.0039	5.2258
5-[(Z)-5-Hydroxy-3-methylpent-3-enyl]-1,4a-dimethyl-6-methylidene -3,4,5,7,8,8a-hexahydro-2H-naphthalene-1-carboxylic acid	2.5900	321.2426	POS	C20H32O3				0.1042	0.0752	1.1047	0.0496	0.7215
DG(18:4(6Z,9Z,12Z,15Z)/18:1(11Z)/0:0)	2.5900	615.4982	POS	C39H66O5	HMDB0007333			0.0601	0.0432	1.4027	0.0125	0.7191
N-Benzyl-2-naphthalenesulfonamide	2.5800	296.0780	NEG	C17H15NO2S				0.0695	0.1079	1.1238	0.0385	1.5525
2-(Benzoylamino)-4,5,6,7-tetrahydro-1-benzothiophene-3-carboxylic acid	2.5800	300.0728	NEG	C16H15NO3S				0.0852	0.1697	1.5753	0.0225	1.9911
Mardepodect	2.5800	393.1687	POS	C25H20N4O	HMDB0256365			0.0080	0.0153	2.2222	0.0012	1.9037
(E)-2'-Geranyl-3',4',7-trihydroxyflavanone	2.5800	409.1970	POS	C25H28O5	HMDB0040317	113866-90-3		0.0131	0.0220	2.0081	0.0021	1.6774
N,N-Dimethyl-4-(6-methylbenzo[d]thiazol-2-yl)aniline	2.5800	269.1134	POS	C16H16N2S				0.0605	0.0908	1.6915	0.0084	1.5015
Dehydrogingerdione	2.5800	345.2105	NEG	C21H30O4	HMDB0029476	82206-04-0		1.6106	0.9901	2.0087	0.0084	0.6147
Nervobscurine	2.5700	383.1924	POS	C22H26N2O4				0.0104	0.0236	1.5423	0.0016	2.2604
Mulberrofuran_A	2.5700	393.2019	POS	C25H28O4	HMDB0033656	68978-04-1	C08846	0.0084	0.0199	1.8824	0.0055	2.3727
Licochalcone A	2.5700	337.1409	NEG	C21H22O4				0.1466	0.5819	1.6223	0.0115	3.9705
2,2'-(3-(Trifluoromethyl)phenylazanediyl)diethanol	2.5700	250.1045	POS	C11H14F3NO2				1.1071	0.4224	1.9778	0.0012	0.3816
Triptophenolide	2.5700	311.1687	NEG	C20H24O3				20.4262	34.4241	1.5527	0.0004	1.6853
PC(33:1)	2.5600	728.5670	POS	C41H80NO8P				0.1334	0.0384	1.3413	0.0021	0.2879
Rutamarin	2.5600	357.1659	POS	C21H24O5	HMDB0030666	14882-94-1	C09308	0.0202	0.0322	1.6233	0.0479	1.5993
1-(4-Nitrophenyl)-3-phenyl-1H-pyrazol-5-ylamine	2.5600	281.1064	POS	C15H12N4O2				0.0237	0.0162	1.5087	0.0243	0.6826
Quinoline, 4-[6-[4-(1-piperazinyl)phenyl]pyrazolo[1,5-a]pyrimidin-3-yl]-	2.5500	407.2027	POS	C25H22N6	HMDB0244322			0.0025	0.0042	1.7159	0.0169	1.6959
(6R,12aS,12bS,13R)-8,9-Dimethoxy-5,6-dihydro-3H,4H-6,12b,12a -(propane[1,1,3]triyl)-3a,10b-diazabenzo[7,8]cycloocta[1,2,3-cd]inden-11(12H)-one	2.5500	353.1819	POS	C21H24N2O3				0.0068	0.0108	1.0258	0.0415	1.5994
4-[(2,2-Diphenylacetyl)oxy]-1,1-dimethylpiperidinium cation	2.5500	324.1920	POS	C21H26NO2				0.0307	0.0522	1.1444	0.0199	1.7038
N-(Naphthalen-2-yl)-2-sulfanylacetamide	2.5500	216.0514	NEG	C12H11NOS				0.4615	0.6942	1.5151	0.0263	1.5041

12-Methoxy-8,11,13-abietatrien-20,11-olide	2.5500	329.2072	POS	C21H28O3	HMDB0038391			0.0065	0.0110	1.5683	0.0207	1.6928
Methanone, [1-(2-hydroxypentyl)-1H-indol-3-yl](2,2,3,3-tetramethylcyclopropyl)-	2.5500	328.2232	POS	C21H29NO2				0.0777	0.0463	2.6643	0.0016	0.5963
1-Acetylpiperidine-2-carboxylic acid	2.5500	172.0969	POS	C8H13NO3				0.0340	0.0503	2.0840	0.0061	1.4817
5-Hydroxymethyltolterodine	2.5500	342.2389	POS	C22H31NO2	HMDB0013973			0.0948	0.0456	2.2048	0.0055	0.4806
2,4-Dodecadienamide, N-(p-hydroxyphenethyl)-, (E,E)-	2.5500	314.2088	NEG	C20H29NO2				0.9419	0.4616	2.4495	0.0105	0.4901
N-(.alpha.-Linolenoyl)tyrosine	2.5500	440.2771	NEG	C27H39NO4				0.6524	0.3778	1.4101	0.0073	0.5790
1-(2-Chloro-4-nitrophenyl)-3-methylpiperazine	2.5500	256.0818	POS	C11H14ClN3O2				0.0261	0.1167	1.8668	0.0043	4.4735
1-Monolinolenin	2.5500	353.2688	POS	C21H36O4				0.0741	0.0578	1.5294	0.0371	0.7796
Apixaban	2.5500	460.2025	POS	C25H25N5O4	HMDB0248515			0.0259	0.0137	1.3183	0.0176	0.5289
Nateglinide	2.5500	316.1879	NEG	C19H27NO3	HMDB0014869	105816-04-4	C12508	1.3493	0.7507	2.8035	0.0039	0.5563
3-(1,1-Dimethyl-2-propenyl)-8-(3-methyl-2-butenyl)xanthyletin	2.5400	365.2070	POS	C24H28O3	HMDB0030730	30310-55-5		0.0145	0.0698	1.2151	0.0296	4.8294
.beta.-Alanine, N-[2-(2-pyridinyl)-6-(1,2,4,5-tetrahydro-3H-3-benzazepin-3-yl)-4-pyrimidinyl]-, ethyl ester	2.5300	418.2183	POS	C24H27N5O2	HMDB0252948			0.0017	0.0039	1.5192	0.0191	2.3314
6-Ketoestriol	2.5300	303.1553	POS	C18H22O4	HMDB0000530	7323-86-6		0.0441	0.0671	1.6422	0.0105	1.5212
Galdosol	2.5300	345.1661	POS	C20H24O5				0.0375	0.0366	1.3310	0.0183	0.9754
Cryptotanshinone	2.5300	297.1445	POS	C19H20O3				0.1346	0.8867	1.6224	0.0110	6.5853
Duloxetine	2.5300	298.1287	POS	C18H19NOS	HMDB0014619	136434-34-9		0.2687	1.0155	1.9755	0.0045	3.7797
Paynantheine	2.5300	397.2121	POS	C23H28N2O4				0.1120	1.2407	2.0342	0.0004	11.0783
5-Chloro-2-(4-propionyl-1-piperazinyl)aniline	2.5300	268.1181	POS	C13H18ClN3O				0.0819	0.5046	2.0075	0.0019	6.1633
Nalbuphine	2.5300	358.1966	POS	C21H27NO4	HMDB0014982	20594-83-6	C07251	0.0078	0.0155	1.3511	0.0207	1.9804
Fostamatinib	2.5300	579.1485	NEG	C23H26FN6O9P	HMDB0252469	901119-35-5	D09347	0.0353	0.0149	1.8559	0.0385	0.4213
(3aS,5S,11bR,E)-12-Ethylidene-1,2,3a,4,5,7-hexahydro-3,5-ethanopyrrolo[2,3-d]carbazole-6-carbaldehyde	2.5200	293.1612	POS	C19H20N2O				0.0160	0.0253	1.3370	0.0274	1.5833
(-)-Epigallocatechin	2.5200	305.0702	NEG	C15H14O7	HMDB0038361	970-74-1	C12136	12.3492	3.7730	2.0780	0.0149	0.3055
Midafotel	2.5200	249.0616	NEG	C8H15N2O5P				0.8875	1.5275	1.1595	0.0216	1.7211
vesnarinone	2.5200	396.1970	POS	C22H25N3O4	HMDB0042059	81840-15-5		0.0123	0.0518	1.3913	0.0131	4.2131

DG(20:3(8Z,11Z,14Z)/14:1(9Z)/0:0)	2.5200	589.4821	POS	C37H64O5	HMDB0007473			0.3077	0.0908	2.9251	0.0000	0.2951
Tri(butoxyethyl)phosphate	2.5200	399.2465	POS	C18H39O7P	HMDB0259275		C14446	0.0659	0.3995	1.5574	0.0067	6.0626
N.alpha.-Benzoyl-DL-arginine-4-nitroanilide	2.5100	399.1762	POS	C19H22N6O4	HMDB0249046			0.0073	0.0115	1.5047	0.0358	1.5756
4-Hydroxycyclofenil	2.5100	295.1300	NEG	C19H20O3				0.5792	3.6423	1.6036	0.0058	6.2890
(S)-Edulinine	2.5100	292.1546	POS	C16H21NO4	HMDB0030178	27495-36-9		0.0693	0.0263	1.8553	0.0021	0.3797
Ethyl 3-(piperidin-4-yl)propanoate	2.5100	186.1490	POS	C10H19NO2				0.0120	0.0153	1.9925	0.0496	1.2743
gamma-L-Glutamyl-L-pipecolic_acid	2.5100	259.1290	POS	C11H18N2O5	HMDB0038614			0.1042	0.1673	1.3644	0.0345	1.6055
Beta-Ecdysterone	2.5100	503.2828	POS	C27H44O7	HMDB0030180	5289-74-7		0.0062	0.0053	1.9927	0.0446	0.8507
8-Hydroxycarvedilol	2.5000	423.1975	POS	C24H26N2O5	HMDB0013946			0.0037	0.0090	2.1004	0.0142	2.3994
N-(3-Chloro-4-methylphenyl)-4-nitrobenzamide	2.5000	291.0492	POS	C14H11ClN2O3				0.0381	0.0240	2.2674	0.0096	0.6295
4-[2-(2,6-dimethoxy-4-prop-2-enylphenoxy)-1-hydroxypropyl]-2-methoxyphenol	2.4900	375.1764	POS	C21H26O6	HMDB0039248	52190-21-3		0.0313	0.0530	1.9987	0.0076	1.6920
1,3,5-Tris(4-hydroxyphenyl)benzene	2.4900	355.1291	POS	C24H18O3				0.0161	0.1502	1.7942	0.0030	9.3532
Isopentenyladenine	2.4900	202.1085	NEG	C10H13N5	HMDB0245646		C04083	0.2883	0.1422	1.4347	0.0307	0.4932
Dihydroferuperine	2.4800	290.1710	POS	C17H23NO3	HMDB0040700	77795-17-6		0.0361	0.0270	2.1353	0.0216	0.7495
Piracetam	2.4800	143.0815	POS	C6H10N2O2	HMDB0256585			0.0669	0.0709	1.3903	0.0296	1.0594
Ginkgolic Acid C17-1	2.4800	375.2895	POS	C24H38O3	HMDB0038522	111047-30-4		0.2461	0.1429	1.7661	0.0462	0.5807
PA(22:0/20:5(5Z,8Z,11Z,14Z,17Z))	2.4800	779.5605	POS	C45H79O8P	HMDB0115258			0.0144	0.0075	1.5200	0.0084	0.5246
N-[2-(4-Prenyloxyphenyl)ethyl]tiglamide	2.4700	288.1918	POS	C18H25NO2	HMDB0041142	172837-74-0		0.0677	0.0434	2.6975	0.0030	0.6408
Dicyclomine	2.4700	310.2740	POS	C19H35NO2	HMDB0014942	77-19-0	C06951	0.1018	0.0476	1.8081	0.0253	0.4671
(R)-2-Hydroxysterculic_acid	2.4700	311.2583	POS	C19H34O3	HMDB0031060	14602-38-1		0.0723	0.0369	2.0450	0.0061	0.5110
N-[4-(4-Ethylpiperazin-1-yl)phenyl]benzo[g]quinolin-4-amine	2.4600	383.2286	POS	C25H26N4				0.0115	0.0168	1.1916	0.0274	1.4551
Erianin	2.4600	319.1512	POS	C18H22O5	HMDB0251918			0.1012	0.2117	1.4545	0.0149	2.0922
Pectenotoxin_2	2.4500	859.4966	POS	C47H70O14	HMDB0033491	97564-91-5		0.0067	0.0080	1.1502	0.0155	1.1915
3-Methyl-5-pentyl-2-furannonanoic_acid	2.4500	309.2426	POS	C19H32O3	HMDB0031091	57818-39-0		0.0616	0.0523	1.6206	0.0183	0.8491
Cembratetraene-16,2:19,6-diolide	2.4400	329.1710	POS	C20H24O4				0.0187	0.0332	2.2864	0.0061	1.7770
Cys-Tyr	2.4400	283.0784	NEG	C12H16N2O4S				6.0920	3.7376	1.7346	0.0169	0.6135

N-Pentadecanoyl-L-homoserine lactone	2.4300	326.2690	POS	C19H35NO3				0.0156	0.0080	1.6589	0.0446	0.5124
Arnamiol	2.4100	451.1918	POS	C24H31ClO6	HMDB0035062	102092-23-9		0.0017	0.0025	1.2591	0.0430	1.4658
Kiwiionoside	2.4100	407.2294	POS	C19H34O9	HMDB0038691	141897-12-3		0.0025	0.0043	1.6044	0.0136	1.7521
Queuosine	2.4000	410.1668	POS	C17H23N5O7	HMDB0011596	57072-36-3		0.0053	0.0094	1.4515	0.0017	1.7755
4,5-Dihydro-2-methylthiazole	2.4000	102.0372	POS	C4H7NS	HMDB0029555	2346-00-1		0.0038	0.0020	2.2114	0.0019	0.5332
3-(1-Isobutyl-1H-tetraazol-5-yl)aniline	2.3900	218.1389	POS	C11H15N5				0.0380	0.0402	1.0444	0.0285	1.0586
4-[(3,4,5-Trimethoxybenzoyl)amino]butanoic acid	2.3900	296.1144	NEG	C14H19NO6				2.0902	8.5090	1.6981	0.0100	4.0709
Glycochenodeoxycholic_acid_3-glucuronide	2.3900	626.3549	POS	C32H51NO11	HMDB0002579	79254-98-1	C03033	0.0025	0.0048	1.3429	0.0332	1.9044
Tsugaric_acid_A_21-glucosyl_ester	2.3800	661.4371	POS	C38H60O9	HMDB0033425	205119-13-7		0.0172	0.0092	2.0582	0.0155	0.5380
a-L-Fucopyranosyl-(1->2)-b-D-galactopyranosyl-(1->2)-D-xylose	2.3800	459.1739	POS	C17H30O14	HMDB0041222	130136-27-5		0.0004	0.0012	2.4107	0.0008	2.7324
O-t-Butyl-L-threonine methyl ester	2.3700	190.1438	POS	C9H19NO3				0.7214	0.5190	1.0918	0.0142	0.7194
N-[1-Oxo-3-(4-oxo-1,2,3-benzotriazin-3(4H)-yl)propyl]glycine	2.3700	275.0774	NEG	C12H12N4O4				0.4533	0.7178	1.1509	0.0169	1.5834
Phlorin	2.3700	287.0775	NEG	C12H16O8				1.0901	1.9409	1.3814	0.0142	1.7805
Desmethyleneparoxetine	2.3700	316.1350	NEG	C18H20FNO3				20.9895	13.9435	1.7629	0.0415	0.6643
N-Hexadecanoyl-L-homoserine lactone	2.3700	340.2847	POS	C20H37NO3				0.1422	0.0827	1.6744	0.0088	0.5817
3-(1H-Benzimidazol-2-yl)propanohydrazide	2.3600	203.0925	NEG	C10H12N4O				0.2963	0.1948	1.7869	0.0183	0.6574
2-Oleoyl-1-palmitoyl-sn-glycero-3-phosphoserine	2.3600	762.5335	POS	C40H76NO10P	HMDB0012357			0.0101	0.0038	1.5379	0.0149	0.3722
Thyrotropin-Releasing Hormone	2.3500	363.1764	POS	C16H22N6O4	HMDB0259062			0.1197	0.1936	1.4095	0.0496	1.6172
(R)-4-((1R,3S,5S,7R,8S,9S,10S,12S,13R,14S,17R)-1,3,7,12-tetrahydroxy -10,13-dimethylhexadecahydro-1H-cyclopenta[a]phenanthren-17-yl)pentanoic acid	2.3500	407.2793	POS	C24H40O6	HMDB0000307	80875-94-1		1.4668	0.7688	1.2417	0.0234	0.5241
PC(22:6(4Z,7Z,10Z,13Z,16Z,19Z)/18:2(9Z,12Z))	2.3500	830.5778	POS	C48H80NO8P	HMDB0008730		C00157	1.3351	0.6981	1.2628	0.0446	0.5229
4-((N-(4-((N-Acetylvalylvalyl)amino)-3-hydroxy-6-methylheptanoyl) alanyl)amino)-2,4,5,6-tetradeoxy-6-methylheptonic acid	2.3300	660.4101	POS	C31H57N5O10				0.0059	0.0260	1.3784	0.0100	4.4193
PC(14:1(9Z)/16:1(9Z))	2.3300	702.4997	POS	C38H72NO8P	HMDB0007903			0.0233	0.0413	1.6661	0.0004	1.7730
Kinetin	2.3300	214.0722	NEG	C10H9N5O	HMDB0012245	525-79-1	C08272	0.2538	0.3492	1.6072	0.0105	1.3760
Muzanzagenin	2.3200	443.2728	POS	C27H38O5	HMDB0032601	197080-19-6		0.0785	0.3860	1.2769	0.0149	4.9179

1,25-Dihydroxyvitamin_D3-26,23-lactone	2.3200	445.2885	POS	C27H40O5	HMDB0000969	81203-50-1		0.0350	0.2009	1.8963	0.0007	5.7435
10'-apo-beta-carotenal	2.3100	377.2801	POS	C27H36O	HMDB0059605	640-49-3		0.8881	0.4809	1.1823	0.0345	0.5415
Carnosol	2.3100	353.1710	POS	C20H26O4	HMDB0002121	5957-80-2	C09069	0.0649	0.2462	1.6957	0.0155	3.7915
N6-2-(4-Aminophenyl)ethyladenosine	2.3000	387.1764	POS	C18H22N6O4				0.0112	0.0245	2.1374	0.0018	2.1905
Oleic acid 2,6-diisopropylanilide	2.3000	442.4006	POS	C30H51NO				0.0086	0.0042	1.1932	0.0169	0.4856
2-Oleoyl-1-stearoyl-sn-glycero-3-phosphoserine	2.3000	790.5526	POS	C42H80NO10P	HMDB0010163	124262-93-7	C02737	0.0046	0.0064	2.1329	0.0005	1.3688
15-Ketofluprostenol	2.3000	457.1771	POS	C23H27F3O6				0.0021	0.0028	1.4384	0.0371	1.3201
Kendomycin	2.3000	487.2990	POS	C29H42O6				0.0502	0.2108	1.4196	0.0199	4.2022
Bufotenine_O-glucoside	2.3000	367.1864	POS	C18H26N2O6	HMDB0029564	64656-15-1		0.2408	0.7030	1.5882	0.0136	2.9196
Clobetasol	2.3000	411.1718	POS	C22H28ClFO4	HMDB0015148	25122-46-7		0.0015	0.0025	1.8593	0.0183	1.7439
DG(20:4(5Z,8Z,11Z,14Z)/14:1(9Z)/0:0)	2.3000	587.4673	POS	C37H62O5	HMDB0007502			0.1292	0.0403	2.8598	0.0001	0.3122
Mitragynine	2.2900	399.2280	POS	C23H30N2O4	HMDB0041933	6202-22-8	C09226	0.0524	0.6262	1.6419	0.0020	11.9450
3-Oxocyclobutanecarboxylic acid	2.2800	113.0244	NEG	C5H6O3				0.8740	0.5591	1.1945	0.0332	0.6397
Panaxydiol	2.2800	261.1809	POS	C17H24O2	HMDB0304717			0.0263	0.0659	1.7140	0.0191	2.5112
(22E,_24x)-Ergosta-4,6,8,22-tetraen-3-one	2.2800	393.3116	POS	C28H40O	HMDB0030898	194721-75-0		0.0913	0.0570	1.0698	0.0496	0.6247
L-Thyronine	2.2800	272.0930	NEG	C15H15NO4	HMDB0000667	1596-67-4		1.0700	1.9468	2.0692	0.0012	1.8194
.beta.-Hydroxyphenylalanine	2.2700	180.0667	NEG	C9H11NO3	HMDB0244981			5.3239	2.9547	2.4683	0.0415	0.5550
3-Hydroxymugineic_acid	2.2700	337.1266	POS	C12H20N2O9	HMDB0033927	74235-23-7		0.0095	0.1402	1.6162	0.0029	14.7774
LACTULOSE	2.2700	360.1504	POS	C12H22O11	HMDB0000740	4618-18-2	C07064	0.2087	0.1188	1.4310	0.0131	0.5695
Tacrolimus	2.2700	804.4879	POS	C44H69NO12	HMDB0015002	104987-11-3		0.0670	0.3813	1.1745	0.0319	5.6940
Dimethyl_3-methoxy-4-oxo-5-(8,11,14-pentadecatrienyl)-2-hexenedioate	2.2700	421.2594	POS	C24H36O6	HMDB0032099			0.0944	0.4240	1.4000	0.0149	4.4921
Metalaxyl	2.2600	280.1544	POS	C15H21NO4	HMDB0031802		C10947	0.1628	0.0178	1.4934	0.0142	0.1095
Canthaxanthin	2.2600	564.4005	POS	C40H52O2	HMDB0003154	514-78-3	C08583	0.0361	0.0119	1.6677	0.0225	0.3303
Glycerol_trihexanoate	2.2600	387.2718	POS	C21H38O6	HMDB0031125	621-70-5		0.0432	0.0234	2.2713	0.0131	0.5421
Fenoldopam	2.2600	304.0708	NEG	C16H16ClNO3	HMDB0014938	67227-57-0	C07693	3.1125	1.3447	2.0470	0.0061	0.4320
Flibanserin	2.2500	391.1713	POS	C20H21F3N4O	HMDB0252304			0.0449	0.0798	1.8561	0.0371	1.7779

Hexanamide, N-[(1S,2R,3E)-2-hydroxy-1-(hydroxymethyl)-3-heptadecen	2.2500	576.3761	POS	C30H49N5O6				0.0761	0.0411	2.1802	0.0307	0.5398
-1-yl]-6-[(7-nitro-2,1,3-benzoxadiazol-4-yl)amino]-												
Solacauline	2.2500	824.4851	POS	C43H69NO14	HMDB0029373			0.0235	0.1208	1.1546	0.0274	5.1357
1-Methyl-3-(trifluoromethyl)benzo[c][1,8]naphthyridin-6(5H)-one	2.2500	277.0591	NEG	C14H9F3N2O				0.6163	0.3447	2.1095	0.0415	0.5593
3-(1-Hydroxy-3-methylbutyl)-6-(2-hydroxy-4-methyl-6-(((pentofuranosyl	2.2500	785.2852	NEG	C36H50O19				1.3885	3.7480	1.7465	0.0039	2.6994
-(1->3)pentopyranosyl-(1->4)pentopyranosyl)oxy)methyl)phenoxy)-2-methoxybenzoic acid												
Ethyl 3-(N-butylacetamido)propionate	2.2500	216.1595	POS	C11H21NO3	HMDB0246904		C18830	0.0770	0.2034	1.0410	0.0462	2.6417
N1,N3-Bis(4-methylphenyl)malonamide	2.2400	283.1400	POS	C17H18N2O2				0.0238	0.0415	1.2730	0.0234	1.7453
(1S,3R,4R,5R)-3-(((2E)-3-(2-(3,4-Dihydroxyphenyl)-7-hydroxy-3-(methoxycarbonyl)	2.2400	545.1295	NEG	C26H26O13				0.1188	0.0467	2.7402	0.0020	0.3933
-2,3-dihydro-1-benzofuran-5-yl)prop-2-enoyl)oxy)-1,4,5-trihydroxycyclohexanecarboxylic acid												
Ionomycin	2.2400	709.5149	POS	C41H72O9				0.0300	0.0594	2.0331	0.0006	1.9775
3-{[(1Z)-4-(4-Hydroxyphenyl)phthalazin-1(2H)-ylidene]amino}phenol	2.2300	328.1072	NEG	C20H15N3O2				2.6267	1.1146	2.8171	0.0096	0.4243
1-Phenylicosane-1,3-dione	2.2300	387.3221	POS	C26H42O2	HMDB0032925	58446-52-9		0.0189	0.0146	1.5614	0.0199	0.7709
Carebastine	2.2300	500.2759	POS	C32H37NO4				0.0806	0.0248	2.1816	0.0020	0.3080
Methyl 2-(3,5-dichloro-2-hydroxy-6-methoxy-4-methylbenzoyl)-3,5-dihydroxybenzoate	2.2300	399.0013	NEG	C17H14Cl2O7				2.0233	6.9281	1.8771	0.0005	3.4241
(7'R,8'R)-4,7'-Epoxy-3'-methoxy-4',5,9,9'-lignanetetrol_9'-glucoside	2.2300	509.1991	POS	C25H32O11	HMDB0038710			0.0371	0.0103	2.9781	0.0001	0.2781
(3beta,23E)-3-Hydroxy-27-norcycloart-23-en-25-one	2.2200	427.3615	POS	C29H46O2	HMDB0037382	132943-49-8		0.1443	0.0667	1.7253	0.0155	0.4622
PC(16:1(9Z)/14:1(9Z))	2.2200	702.5008	POS	C38H72NO8P	HMDB0007999			0.0079	0.0122	1.6761	0.0055	1.5358
Vitamin K1 2,3-epoxide	2.2000	467.3483	POS	C31H46O3				0.0839	0.0254	1.7618	0.0496	0.3024
PS(14:1(9Z)/14:1(9Z))	2.2000	676.4117	POS	C34H62NO10P	HMDB0012342			0.0028	0.0128	1.4657	0.0115	4.6322
Ciprofibrate	2.2000	287.0230	NEG	C13H14Cl2O3	HMDB0250268			8.5402	4.2861	1.7643	0.0073	0.5019
Armillatin	2.2000	611.4269	POS	C38H58O6	HMDB0038743	139051-17-5		0.0179	0.0069	2.3076	0.0084	0.3833
5-(10,13-Nonadecadienyl)-1,3-benzenediol	2.2000	373.3063	POS	C25H40O2	HMDB0039867	189562-02-5		0.0732	0.0459	1.3903	0.0400	0.6271
DOCOSANOL	2.2000	365.3165	POS	C22H46O	HMDB0014770	30303-65-2		0.2926	0.1423	1.3266	0.0052	0.4863
Murrayazolinine	2.2000	350.2076	POS	C23H27NO2	HMDB0030183	49620-01-1		0.0553	0.0392	2.1041	0.0496	0.7083
(S)-(-)-Perillyl_alcohol	2.2000	135.1169	POS	C10H16O	HMDB0036087	57717-97-2		0.0145	0.0198	1.3879	0.0479	1.3591

Myrtucommulone B	2.1900	415.2100	POS	C24H30O6				0.0153	0.0290	1.8133	0.0296	1.8975
4-((1E)-3-(2,4-Dihydroxyphenyl)-3-oxoprop-1-en-1-yl)phenyl 6	2.1900	563.1528	NEG	C30H28O11				0.0418	0.0136	2.4134	0.0004	0.3242
-O-((2E)-3-(4-hydroxyphenyl)prop-2-enoyl)-.beta.-D-glucopyranoside												
2-Acetylthiazole	2.1900	128.0178	POS	C5H5NOS	HMDB0032964	24295-03-2		0.0258	0.0144	1.2311	0.0319	0.5600
Neferine	2.1900	625.3230	POS	C38H44N2O6	HMDB0034104	2292-16-2		0.0068	0.0154	1.7659	0.0131	2.2526
1H-Indole-3-carboxamide, 1-(5-fluoropentyl)-N-2-naphthalenyl-	2.1800	375.1916	POS	C24H23FN2O				0.0121	0.0241	1.3839	0.0010	1.9943
4'-Methoxymucidin	2.1800	289.1396	POS	C17H20O4	HMDB0030019	86421-33-2		0.0238	0.0357	1.2490	0.0371	1.5025
(22E,24R)-Stigmasta-4,22-diene-3,6-dione	2.1800	425.3460	POS	C29H44O2	HMDB0038656	50868-51-4		0.0863	0.0372	1.4213	0.0462	0.4308
PS(14:1(9Z)/18:4(6Z,9Z,12Z,15Z))	2.1700	726.4423	POS	C38H64NO10P	HMDB0112309			0.0033	0.0105	1.1721	0.0358	3.1580
LysoPC(22:4(7Z,10Z,13Z,16Z))	2.1700	572.3793	POS	C30H54NO7P	HMDB0010401		C04230	0.0076	0.0055	2.5423	0.0076	0.7241
4-Hydroxyphenyl 3,6-bis-O-((2E)-3-(1-hydroxy-4-oxocyclohexa	2.1600	595.1426	NEG	C30H28O13				0.0438	0.0134	2.0132	0.0002	0.3057
-2,5-dien-1-yl)prop-2-enoyl)-.beta.-D-glucopyranoside												
2,6-Dichloro-N-(3-methoxypropyl)benzamide	2.1600	262.0381	POS	C11H13Cl2NO2				0.0241	0.0151	1.4615	0.0183	0.6275
Hexyl 6-O-pentopyranosylhexopyranoside	2.1600	395.1976	NEG	C17H32O10				0.3208	1.6590	1.6131	0.0025	5.1714
Racemoramide	2.1500	393.2499	POS	C25H32N2O2	HMDB0251099			0.0110	0.0078	2.0170	0.0149	0.7037
(-)-Bilobalide	2.1500	325.0889	NEG	C15H18O8	HMDB0242203			0.5612	0.3590	1.5465	0.0415	0.6396
3,3'-Disulfanediylbis(2-(2,2,2-trifluoroacetamido)propanoic acid)	2.1400	430.9843	NEG	C10H10F6N2O6S2				2.3601	5.1162	1.7000	0.0041	2.1678
Ascofuranone	2.1300	421.1808	POS	C23H29ClO5				0.0034	0.0051	1.3726	0.0496	1.5052
1-(3,4-Dihydroxy-5-methoxyphenyl)-7-(3,4-dihydroxyphenyl)heptan-3-one	2.1300	359.1460	NEG	C20H24O6				2.5315	0.4806	3.1044	0.0000	0.1899
5-O-Desmethyldonepezil	2.1200	366.2025	POS	C23H27NO3	HMDB0013958			0.0560	0.0368	2.1264	0.0385	0.6569
Alnuside A	2.1200	461.1854	NEG	C24H30O9				0.3011	0.4354	1.1934	0.0307	1.4460
Trimebutine	2.1200	388.2081	POS	C22H29NO5	HMDB0259230			0.0109	0.0225	2.0231	0.0035	2.0617
Reserpine	2.1200	609.3279	POS	C33H40N2O9	HMDB0014351	50-55-5	C06539	0.0224	0.0443	1.5083	0.0462	1.9800
N-Butylscopolaminium cation	2.1100	360.2129	POS	C21H30NO4				0.0419	0.0267	2.4944	0.0084	0.6388
Prostaglandin A2-biotin	2.1100	645.3986	POS	C35H56N4O5S				0.0066	0.0317	1.1144	0.0125	4.8077
2,4-Dihydroxy-1-methyl-8-methylidene-13-oxododecahydro-4a,1	2.1100	347.1462	NEG	C19H24O6				0.6846	0.5933	1.4129	0.0479	0.8667

-(epoxymethano)-7,9a-methanobenzo[a]azulene-10-carboxylic acid											
Lactupicrin	2.1100	409.1257	NEG	C23H22O7			0.8512	0.4494	1.7478	0.0479	0.5280
Ramipril	2.1100	417.2462	POS	C23H32N2O5	HMDB0014324	87333-19-5	0.0228	0.0148	1.9922	0.0120	0.6494
Benzo[b]thiophene-2-carboxamide, 3-chloro-N-[trans-4	2.1000	490.1770	POS	C28H28ClN3OS			0.0052	0.0027	1.8833	0.0055	0.5123
-(methylamino)cyclohexyl]-N-[[3-(4-pyridinyl)phenyl]methyl]-											
Vaccinoside	2.1000	535.1519	NEG	C25H28O13			0.4784	0.2486	1.3058	0.0136	0.5196
1-Hexadecyl-2-azelaoyl-sn-glycero-3-phosphocholine	2.1000	652.4614	POS	C33H66NO9P			0.0991	0.0340	2.0664	0.0199	0.3432
N-(1-Amino-3,3-dimethyl-1-oxobutan-2-yl)-1-pentyl-1H-indole-3-carboxamide	2.0900	344.2281	POS	C20H29N3O2			0.0439	0.0698	1.6032	0.0136	1.5912
Syringolin C	2.0800	506.3055	NEG	C25H41N5O6			0.6287	0.1762	1.8629	0.0430	0.2803
Isomucronulatol	2.0800	301.1042	NEG	C17H18O5			3.5585	1.7406	2.6378	0.0034	0.4891
Momordicoside_D	2.0800	783.4803	POS	C42H70O13	HMDB0035011	78887-73-7	0.0068	0.0417	1.2025	0.0055	6.1650
Acetamide, N-[2-[5-(acetyloxy)-3,6-dimethoxy-1-phenanthrenyl]ethyl]-N-methyl-	2.0700	396.1863	POS	C23H25NO5			0.0020	0.0146	1.7088	0.0430	7.2154
Erlotinib	2.0700	394.1717	POS	C22H23N3O4	HMDB0014671	183321-74-6	0.0041	0.0080	1.8549	0.0120	1.9277
1H-Indazole-3-carboxamide, N-[1-(aminocarbonyl)-2,2-dimethylpropyl]-1-pentyl-	2.0600	345.2244	POS	C19H28N4O2			0.0641	0.0399	2.0695	0.0080	0.6218
Glutathione ethyl ester	2.0600	336.1264	POS	C12H21N3O6S	HMDB0252819		0.0089	0.6695	1.8748	0.0183	75.2606
Fellutamide B	2.0500	556.3632	POS	C27H49N5O7			0.0834	0.0195	2.2449	0.0400	0.2341
4-Methoxybenzyl_O-(2-sulfoglucoside)	2.0500	381.0795	POS	C14H20O10S	HMDB0034754		0.1269	0.0858	1.1440	0.0479	0.6764
Piperidolate	2.0400	324.1917	POS	C21H25NO2			0.0228	0.0380	1.1801	0.0319	1.6684
(2-aminoethoxy)[2-[octadec-9-enoyloxy]-3-[octadeca-1.11-dien-1-yloxy]propoxy]phosphinic acid	2.0400	726.5327	NEG	C41H78NO7P	HMDB0011408		0.0469	0.0085	1.7909	0.0115	0.1813
Dye X-5432-26C	2.0300	400.1356	NEG	C23H19N3O4			0.0196	0.0262	1.1703	0.0110	1.3363
2-{[((2Z)-4-Phenyl-1,3-thiazol-2(3H)-ylidene)amino]carbonyl}benzoic acid	2.0100	323.0543	NEG	C17H12N2O3S			2.5667	5.8826	1.9186	0.0041	2.2919
M553T166	1.9100	553.3386	NEG	C30H50O9			0.4020	0.2586	2.5411	0.0191	0.6432
M549T86	1.9100	549.3067	NEG	C30H48O10			0.3291	0.2070	2.1510	0.0496	0.6291
M196T99	1.8600	196.0616	NEG	C9H11NO4			0.8641	1.3722	1.6049	0.0100	1.5881
3-Methyl-L-tyrosine	1.8300	196.0969	POS	C10H13NO3			0.0377	0.0641	1.4588	0.0479	1.7013
M240T97	1.7600	240.0867	POS	C11H10O5			0.2957	0.4065	1.3017	0.0191	1.3747

M147T70	1.7600	147.0664	NEG	C4H8O2				41.4285	24.7816	1.3449	0.0084	0.5982
Ectoine	1.7500	160.1081	POS	C6H10N2O2	HMDB0240650	96702-03-3	C06231	0.0250	0.0405	1.2223	0.0207	1.6199
M611T221	1.7500	611.1373	NEG	C21H30N6O12S				0.0285	0.0103	1.8997	0.0048	0.3623
M464T177	1.7000	464.1081	NEG	C16H21N5O10				0.8353	0.3385	1.6594	0.0183	0.4053
2'''-N-Acetyl-6'''-deamino-6'''-hydroxyparomomycin II	1.6800	676.3269	POS	C25H46N4O16				0.0024	0.0049	1.5130	0.0430	2.0412
5-Acetamidopentanoate	1.6500	180.0668	NEG	C7H13NO3				1.5557	4.3388	1.7637	0.0025	2.7890
M319T152_1	1.6500	319.1149	NEG	C10H16N2O6				1.3839	0.6964	1.9855	0.0400	0.5032
M505T114	1.6100	505.3017	NEG	C26H40NO5				2.5657	0.7438	1.5831	0.0415	0.2899
M215T104	1.6000	215.0563	NEG	C7H14O6				2.0613	2.8096	1.4714	0.0076	1.3630
N5-Hydroxy-L-ornithine	1.5600	131.0816	POS	C5H12N2O3				0.0329	0.0386	1.5610	0.0243	1.1736
M338T153	1.5600	338.1461	NEG	C16H25N2O7				0.4683	0.2011	1.6548	0.0253	0.4294
M245T168	1.5300	245.0433	NEG	C5H11NO7S				2.1622	1.3152	2.2618	0.0253	0.6083
(3S,4S)-3-Hydroxytetradecane-1,3,4-tricarboxylate	1.5000	367.1723	NEG	C17H30O7				3.6210	1.3793	2.6565	0.0115	0.3809
M254T158	1.4900	254.1137	POS	C11H12N2O4				0.0145	0.0238	2.3878	0.0012	1.6367
M611T175	1.4600	611.3633	NEG	C32H54NO10				0.1051	0.0178	2.3420	0.0050	0.1690
M238T203	1.4500	238.0357	NEG	C10H9NO6				0.0345	0.0650	1.8740	0.0055	1.8828
M245T138	1.4500	245.1133	POS	C10H13NO5				0.0943	0.0729	1.4793	0.0022	0.7735
Harmine	1.4400	193.0718	NEG	C13H12N2O	HMDB0030311	442-51-3	C06538	3.4117	1.9590	2.1817	0.0191	0.5742
2-Hydroxy-5-methylquinone	1.4400	154.0511	NEG	C7H6O3				0.0850	0.1494	1.1543	0.0234	1.7574
M173T69	1.4400	173.0457	NEG	C5H6O3				0.6389	0.8686	1.0028	0.0225	1.3596
M214T99	1.4300	214.0722	NEG	C7H9NO3				0.3766	0.7034	1.7651	0.0385	1.8675
6-O-Methylnorlaudanosoline	1.4200	319.1656	POS	C17H19NO4				0.0327	0.0478	1.3004	0.0319	1.4606
M174T163	1.4200	174.0772	NEG	C7H13NO4				0.2863	0.2450	1.4889	0.0479	0.8557
N(alpha)-Acetyl-L-2,4-diaminobutyrate	1.4100	161.0921	POS	C6H12N2O3				0.0518	0.0391	2.0384	0.0225	0.7547
M525T148	1.4100	525.2349	NEG	C26H38O11				0.2051	0.1630	1.3336	0.0285	0.7947
Cyclo(L-leucyl-L-phenylalanyl)	1.3900	297.0982	NEG	C15H20N2O2				0.6506	0.6577	1.2103	0.0430	1.0109

M226T52_2	1.3500	226.0722	NEG	C10H10O5	0.6237	0.7090	1.3130	0.0479	1.1367
M247T137	1.3200	246.9918	NEG	C8H8O7S	0.9391	0.1915	1.4293	0.0142	0.2039
Anhalonidine	1.3100	206.1176	POS	C12H17NO3	0.0101	0.0209	1.4585	0.0080	2.0754
M296T174	1.3000	296.0988	POS	C12H15N4O3S	0.0103	0.0188	1.9050	0.0052	1.8324
M199T57	1.3000	199.0363	NEG	C10H10O3	0.3405	0.2528	1.3067	0.0234	0.7426
M201T119	1.2800	201.0769	NEG	C7H10O3	0.2992	0.4932	1.3521	0.0142	1.6482
M366T177_2	1.2500	366.1087	NEG	C13H18O11	0.2304	0.1676	1.6154	0.0496	0.7273
N-Acetylbialaphos	1.2400	424.1463	NEG	C13H24N3O7P	0.0904	0.2325	1.6870	0.0115	2.5716
M847T132	1.2100	846.5593	NEG	C52H78O6S	0.2641	0.1909	1.4897	0.0446	0.7229
M220T172	1.1700	220.0486	NEG	C7H8O7	0.0220	0.0307	1.1084	0.0025	1.3935
M291T162_1	1.1400	291.0658	NEG	C10H14N4O5	0.1040	0.1459	1.6151	0.0285	1.4028

Table S4. KEGG Enrichment data matrix before and after a 12-week BBr60 intervention

**Pathway**	**Description**	**Compounds.(dem)**	**Percent**	**Rich_factor**	**p_value**	**up_nums**	**down_nums**	**DA_score**
hsa02010	ABC transporters	C00208;C00407;C00079;C00065;C00140;C00245;C00330;C01083;C05512;C00123;C00064;C00526;C05402	20	0.094202899	5.56158E-08	0	13	-1
hsa04978	Mineral absorption	C00407;C00079;C00065;C00078;C00123;C00064	9.230769231	0.206896552	2.75926E-06	0	6	-1
hsa05230	Central carbon metabolism in cancer	C00407;C00079;C00065;C00078;C00123;C00064	9.230769231	0.162162162	1.23274E-05	0	6	-1
hsa01230	Biosynthesis of amino acids	C00407;C00079;C00065;C00078;C00109;C00123;C00233;C00141;C00064;C01179	15.38461538	0.078125	1.24249E-05	0	10	-1
hsa00290	Valine, leucine and isoleucine biosynthesis	C00407;C00109;C00123;C00233;C00141	7.692307692	0.217391304	1.54134E-05	0	5	-1
hsa01232	Nucleotide metabolism	C00178;C00330;C00106;C05512;C00214;C00064;C00526	10.76923077	0.120689655	1.67553E-05	0	7	-1
hsa04974	Protein digestion and absorption	C00407;C00079;C00065;C00078;C00123;C00064	9.230769231	0.127659574	5.08081E-05	0	6	-1
hsa00260	Glycine, serine and threonine metabolism	C00065;C00078;C00109;C00546;C02737;C06231	9.230769231	0.125	5.74091E-05	2	4	-0.333333333

hsa00970	Aminoacyl-tRNA biosynthesis	C00407;C00079;C00065;C00078;C00123;C00064	9.230769231	0.115384615	9.10031E-05	0	6	-1
hsa01210	2-Oxocarboxylic acid metabolism	C00407;C00079;C00078;C00109;C00123;C00233;C00141;C01179;C01180	13.84615385	0.0625	0.000202027	0	9	-1
hsa00591	Linoleic acid metabolism	C14762;C01595;C14827;C00157	6.153846154	0.142857143	0.000645363	0	4	-1
hsa00240	Pyrimidine metabolism	C00178;C00106;C00214;C00064;C00526	7.692307692	0.078125	0.002205339	0	5	-1
hsa00270	Cysteine and methionine metabolism	C02989;C00065;C00109;C03145;C01180	7.692307692	0.074626866	0.002701369	0	5	-1
hsa00280	Valine, leucine and isoleucine degradation	C00407;C00123;C00233;C00141	6.153846154	0.095238095	0.003028502	0	4	-1
hsa00564	Glycerophospholipid metabolism	C00065;C04230;C00157;C02737	6.153846154	0.071428571	0.008536965	1	3	-0.5
hsa05231	Choline metabolism in cancer	C04230;C00157	3.076923077	0.181818182	0.010555488	0	2	-1
hsa00400	Phenylalanine, tyrosine and tryptophan biosynthesis	C00079;C00078;C01179	4.615384615	0.085714286	0.013846445	0	3	-1
hsa05131	Shigellosis	C00407;C00123	3.076923077	0.142857143	0.016980892	0	2	-1
hsa00470	D-Amino acid metabolism	C00079;C00065;C00064;C01180	6.153846154	0.057971014	0.01746161	0	4	-1
hsa04071	Sphingolipid signaling pathway	C00065;C00319	3.076923077	0.133333333	0.019411164	0	2	-1
hsa00120	Primary bile acid biosynthesis	C00695;C00245;C01921	4.615384615	0.063829787	0.030331059	0	3	-1
hsa04148	Efferocytosis	C04230;C02737	3.076923077	0.095238095	0.036714093	1	1	0
hsa05322	Systemic lupus erythematosus	C02737	1.538461538	0.333333333	0.043068836	1	0	1
hsa00430	Taurine and hypotaurine metabolism	C00245;C14179	3.076923077	0.083333333	0.046930803	1	1	0
hsa00053	Ascorbate and aldarate metabolism	C01620;C01114;C03033	4.615384615	0.052631579	0.049431996	1	2	-0.333333333
hsa04976	Bile secretion	C00695;C01921;C07210;C03033	6.153846154	0.041237113	0.051847637	1	3	-0.5
hsa04150	mTOR signaling pathway	C00123	1.538461538	0.25	0.057015142	0	1	-1
hsa04210	Apoptosis	C00319	1.538461538	0.25	0.057015142	0	1	-1
hsa04973	Carbohydrate digestion and absorption	C00208;C12285	3.076923077	0.074074074	0.058053944	1	1	0
hsa00770	Pantothenate and CoA biosynthesis	C00106;C00141	3.076923077	0.066666667	0.069989068	0	2	-1
hsa03320	PPAR signaling pathway	C14762	1.538461538	0.2	0.070761277	0	1	-1
hsa05140	Leishmaniasis	C02737	1.538461538	0.166666667	0.08431007	1	0	1

hsa00920	Sulfur metabolism	C00065;C00245	3.076923077	0.058823529	0.087014051	0	2	-1
hsa00600	Sphingolipid metabolism	C00065;C00319	3.076923077	0.057142857	0.091448452	0	2	-1
hsa01100	Metabolic pathways	C00695;C00208;C02989;C00407;C00079;C00178;C00065;C00140;C00245;C00078;C00330;C01595;C01083;C01620;C01921;C00106;C12115;C01216;C05512;C00109;C00319;C00123;C00233;C00645;C00214;C01114;C00141;C00064;C01909;C03150;C00954;C00526;C05402;C00884;C01494;C00844;C01179;C01542;C01744;C00546;C05582;C00643;C06730;C14827;C01180;C06202;C00157;C03033;C02737;C06231	76.92307692	0.016265452	0.098699196	8	42	-0.68
hsa00500	Starch and sucrose metabolism	C00208;C01083	3.076923077	0.054054054	0.100510907	0	2	-1
hsa04724	Glutamatergic synapse	C00064	1.538461538	0.125	0.110826746	0	1	-1
hsa05143	African trypanosomiasis	C00078	1.538461538	0.125	0.110826746	0	1	-1
hsa00640	Propanoate metabolism	C00109;C00546	3.076923077	0.048780488	0.119334958	0	2	-1
hsa00380	Tryptophan metabolism	C00078;C00954;C00643	4.615384615	0.036144578	0.119494562	1	2	-0.333333333
hsa04727	GABAergic synapse	C00064	1.538461538	0.111111111	0.123800093	0	1	-1
hsa04726	Serotonergic synapse	C00078;C00643	3.076923077	0.047619048	0.124171629	0	2	-1
hsa00785	Lipoic acid metabolism	C00233;C00141	3.076923077	0.045454545	0.133984712	0	2	-1
hsa04217	Necroptosis	C00319	1.538461538	0.1	0.136587025	0	1	-1
hsa04979	Cholesterol metabolism	C01921	1.538461538	0.1	0.136587025	0	1	-1
hsa00052	Galactose metabolism	C01216;C05402	3.076923077	0.043478261	0.143968744	0	2	-1
hsa05215	Prostate cancer	C07653	1.538461538	0.090909091	0.149190179	0	1	-1
hsa04728	Dopaminergic synapse	C05582	1.538461538	0.083333333	0.161612155	0	1	-1
hsa05146	Amoebiasis	C02737	1.538461538	0.076923077	0.173855518	1	0	1
hsa04080	Neuroactive ligand-receptor interaction	C00245;C16512	3.076923077	0.037735849	0.180011454	0	2	-1
hsa00230	Purine metabolism	C00330;C05512;C00064	4.615384615	0.02970297	0.181358166	0	3	-1
hsa01240	Biosynthesis of cofactors	C00065;C00078;C00141;C00064;C01909;C01179;C03033	10.76923077	0.021341463	0.197980862	1	6	-0.714285714

hsa04964	Proximal tubule bicarbonate reclamation	C00064	1.538461538	0.058823529	0.221092889	0	1	-1
hsa00630	Glyoxylate and dicarboxylate metabolism	C00065;C00064	3.076923077	0.03125	0.238867034	0	2	-1
hsa04723	Retrograde endocannabinoid signaling	C00157	1.538461538	0.052631579	0.243703991	0	1	-1
hsa00910	Nitrogen metabolism	C00064	1.538461538	0.05	0.254765926	0	1	-1
hsa00220	Arginine biosynthesis	C00064	1.538461538	0.043478261	0.287004679	0	1	-1
hsa01040	Biosynthesis of unsaturated fatty acids	C01595;C16513	3.076923077	0.027027027	0.293208343	0	2	-1
hsa00350	Tyrosine metabolism	C01179;C05582	3.076923077	0.025641026	0.314877805	0	2	-1
hsa00590	Arachidonic acid metabolism	C05960;C00157	3.076923077	0.025316456	0.320276322	1	1	0
hsa00250	Alanine, aspartate and glutamate metabolism	C00064	1.538461538	0.035714286	0.337710951	0	1	-1
hsa00750	Vitamin B6 metabolism	C00064	1.538461538	0.034482759	0.347417555	0	1	-1
hsa00780	Biotin metabolism	C01909	1.538461538	0.034482759	0.347417555	0	1	-1
hsa00982	Drug metabolism - cytochrome P450	C16561;C11785	3.076923077	0.022988506	0.363058137	2	0	1
hsa05200	Pathways in cancer	C07653	1.538461538	0.032258065	0.366412441	0	1	-1
hsa00620	Pyruvate metabolism	C00546	1.538461538	0.03125	0.375704677	0	1	-1
hsa00410	beta-Alanine metabolism	C00106	1.538461538	0.03125	0.375704677	0	1	-1
hsa04742	Taste transduction	C00208	1.538461538	0.03125	0.375704677	0	1	-1
hsa05415	Diabetic cardiomyopathy	C00546	1.538461538	0.025641026	0.43709737	0	1	-1
hsa00592	alpha-Linolenic acid metabolism	C00157	1.538461538	0.022727273	0.477270051	0	1	-1
hsa00360	Phenylalanine metabolism	C00079	1.538461538	0.020408163	0.514616715	0	1	-1
hsa00520	Amino sugar and nucleotide sugar metabolism	C00140;C00645	3.076923077	0.016949153	0.517056851	0	2	-1
hsa00071	Fatty acid degradation	C02990	1.538461538	0.02	0.521764407	0	1	-1
hsa00760	Nicotinate and nicotinamide metabolism	C03150	1.538461538	0.018181818	0.555977259	1	0	1
hsa00040	Pentose and glucuronate interconversions	C03033	1.538461538	0.016949153	0.581602255	1	0	1
hsa00330	Arginine and proline metabolism	C00884	1.538461538	0.014492754	0.639466056	1	0	1
hsa00130	Ubiquinone and other terpenoid-quinone biosynthesis	C01179	1.538461538	0.014084507	0.650055041	0	1	-1

hsa05204	Chemical carcinogenesis - DNA adducts	C19255	1.538461538	0.012820513	0.684760621	1	0	1
hsa00140	Steroid hormone biosynthesis	C05490	1.538461538	0.00990099	0.776597737	1	0	1
hsa01250	Biosynthesis of nucleotide sugars	C00140;C00645	3.076923077	0.01	0.796522812	0	2	-1
hsa01200	Carbon metabolism	C00065	1.538461538	0.008928571	0.810643065	0	1	-1
hsa01212	Fatty acid metabolism	C02990	1.538461538	0.008196721	0.837128974	0	1	-1

DA score, Differential Abundance Score

Table S5. Composition of intestinal microbiota in the Bbr60-after and Bbr60-before groups at phylum level.

**Phylum**	**Bbr60-after (%)**	**Bbr60-before (%)**
p__Firmicutes	59.32	62.44
p__Actinobacteriota	22.18	29.11
p__Proteobacteria	12.49	3.99
p__Bacteroidota	5.23	4.00
p__Desulfobacterota	0.51	0.12
p__Fusobacteriota	0.08	0.20
p__Verrucomicrobiota	0.09	0.02
p__Cyanobacteria	0.03	0.06
p__Patescibacteria	0.04	0.04
p__Campylobacterota	0.01	0.01
p__Synergistota	0.02	0.00
p__Planctomycetota	0.00	0.01
p__Gemmatimonadota	0.00	0.00
p__Spirochaetota	0.00	0.00
p__Deinococcota	0.00	0.00
p__Acidobacteriota	0.00	0.00

p__Chloroflexi	0.00	0.00
p__Fibrobacterota	0.00	0.00
p__Methylomirabilota	0.00	0.00
p__Myxococcota	0.00	0.00
p__Armatimonadota	0.00	0.00
p__Candidatus_Saccharibacteria	0.00	0.00
p__Hydrogenedentes	0.00	0.00
p__Deferribacterota	0.00	0.00
p__Nitrospirota	0.00	0.00
p__MBNT15	0.00	0.00
Others	0.00	0.00

Table S6. Composition of intestinal microbiota in the Bbr60-after and Bbr60-before groups at genus level.

**Genus**	**Bbr60-after (%)**	**Bbr60-before (%)**
g__*Bifidobacterium*	18.86	24.45
g__*Streptococcus*	4.71	8.96
g__*Agathobacter*	6.09	5.60
g__*Escherichia-Shigella*	8.63	1.79
g__*Faecalibacterium*	5.31	4.28
g__*Megamonas*	3.47	4.10
g__*Erysipelotrichaceae_UCG-003*	1.01	4.71
g__*Dialister*	4.33	1.27
g__*Romboutsia*	1.24	3.86
g__*Collinsella*	2.04	2.84
g__*Bacteroides*	2.74	1.62
g__*Clostridium_sensu_stricto_1*	2.87	1.39

g__Fusicatenibacter	1.84	2.01
g__Subdoligranulum	1.50	1.90
g__Ruminococcus]_gnavus_group	0.96	2.39
g__Phascolarctobacterium	2.58	0.48
g__Ruminococcus]_torques_group	1.13	1.78
g__Dorea	0.97	1.80
g__Catenibacterium	0.98	1.68
g__Lachnoclostridium	2.17	0.36
g__Blautia	0.97	1.47
g__Klebsiella	1.96	0.40
g__Prevotella_9	0.85	1.42
g__Monoglobus	1.41	0.83
g__Megasphaera	1.20	0.85
g__Ruminococcus	0.73	0.88
g__Veillonella	1.15	0.44
g__UCG-002	0.88	0.58
g__Roseburia	0.71	0.62
g__Coprococcus	0.69	0.57
Others	16.04	14.66
